# Role of the Dihydrodipicolinate Synthase DapA1 on Iron Homeostasis During Cyanide Assimilation by the Alkaliphilic Bacterium *Pseudomonas pseudoalcaligenes* CECT5344

**DOI:** 10.3389/fmicb.2020.00028

**Published:** 2020-01-23

**Authors:** Alfonso Olaya-Abril, María Dolores Pérez, Purificación Cabello, Diego Martignetti, Lara Paloma Sáez, Víctor Manuel Luque-Almagro, Conrado Moreno-Vivián, María Dolores Roldán

**Affiliations:** ^1^Departamento de Bioquímica y Biología Molecular, Universidad de Córdoba, Córdoba, Spain; ^2^Departamento de Botánica, Ecología y Fisiología Vegetal, Edificio Celestino Mutis, Campus de Rabanales, Universidad de Córdoba, Córdoba, Spain

**Keywords:** cyanide, dihydrodipicolinate synthase, dipicolinate, ferric uptake regulator, LC-MS/MS, lysine, proteomic analysis, *Pseudomonas*

## Abstract

Cyanide is a toxic compound widely used in mining and jewelry industries, as well as in the synthesis of many different chemicals. Cyanide toxicity derives from its high affinity for metals, which causes inhibition of relevant metalloenzymes. However, some cyanide-degrading microorganisms like the alkaliphilic bacterium *Pseudomonas pseudoalcaligenes* CECT5344 may detoxify hazardous industrial wastewaters that contain elevated cyanide and metal concentrations. Considering that iron availability is strongly reduced in the presence of cyanide, mechanisms for iron homeostasis should be required for cyanide biodegradation. Previous omic studies revealed that in the presence of a cyanide-containing jewelry residue the strain CECT5344 overproduced the dihydrodipicolinate synthase DapA1, a protein involved in lysine metabolism that also participates in the synthesis of dipicolinates, which are excellent metal chelators. In this work, a *dapA1*^–^ mutant of *P. pseudoalcaligenes* CECT5344 has been generated and characterized. This mutant showed reduced growth and cyanide consumption in media with the cyanide-containing wastewater. Intracellular levels of metals like iron, copper and zinc were increased in the *dapA1*^–^ mutant, especially in cells grown with the jewelry residue. In addition, a differential quantitative proteomic analysis by LC-MS/MS was carried out between the wild-type and the *dapA1*^–^ mutant strains in media with jewelry residue. The mutation in the *dapA1* gene altered the expression of several proteins related to urea cycle and metabolism of arginine and other amino acids. Additionally, the *dapA1*^–^ mutant showed increased levels of the global nitrogen regulator PII and the glutamine synthetase. This proteomic study has also highlighted that the DapA1 protein is relevant for cyanide resistance, oxidative stress and iron homeostasis response, which is mediated by the ferric uptake regulator Fur. DapA1 is required to produce dipicolinates that could act as iron chelators, conferring protection against oxidative stress and allowing the regeneration of Fe-S centers to reactivate cyanide-damaged metalloproteins.

## Introduction

Cyanide, a compound that contains the cyano group (–C≡N), is considered one of the most toxic chemicals, but paradoxically, it was a key molecule in the origin of life (Matthews and Minard, 2006; Sutherland, 2016). In aqueous solution, at alkaline pH cyanide is found as free CN^–^ ion, but at acidic or neutral pH predominates as the volatile cyanhydric acid form (HCN), which presents a pKa 9.2 (Luque-Almagro et al., 2005b; Jaszczak et al., 2017).

Cyanide toxicity derives from its ability to bind metals with high affinity, causing the inhibition of key metalloenzymes (Solomonson, 1981). Thus, the iron/copper-containing terminal oxidase of the respiratory chain (cytochrome *c* oxidase) is inactivated by cyanide, avoiding ATP production. In addition, sublethal doses of cyanide produce a strong inhibition of the tricarboxylic acid (TCA) cycle (Xu et al., 2010). Other factors that contribute to the toxicity of this compound in animals are the inhibition of the oxygen-carrier hemoglobin, the alteration of the dopaminergic and serotonergic systems and the induction of apoptosis (Cassel and Persson, 1992). Although cyanide may have a natural origin, industrial activities produce large scale of heterogeneous wastewaters that contain high concentrations of free cyanide, metals and metal-cyanide complexes (Luque-Almagro et al., 2016; Ibáñez et al., 2017). Cyanide-containing wastes are produced in the synthesis of organic nitriles, nylon, acrylic plastics, paints, dyes, drugs, and chelating agents, but the largest amounts of liquid wastes containing cyanide are generated in gold mines and jewelry and metal processing industries, where cyanide is used for gold extraction and recovery (Eisler and Wiemeyer, 2004; Dash et al., 2009; Xu et al., 2010). The jewelry industry in the city of Córdoba (Spain) produces approximately 10 tons/year of an alkaline wastewater (pH 13) containing approximately 40 g/L of cyanide (about 1.5 M), as both free cyanide and metal-cyanide complexes (Luque-Almagro et al., 2005b; Ibáñez et al., 2017). The main producers of gold are China, Australia, Russia, United States of America, India and several countries of Africa. In Europe, mining activities are also becoming very popular because the demand of gold is increasing (Luque-Almagro et al., 2016). Residues containing cyanide are often discharged to the environment, causing wildlife destruction (Boening and Chew, 1999; Dash et al., 2009).

Chemical treatments to remove cyanide from industrial wastewaters are very expensive and generate products that are also toxic. Therefore, biological treatments to remove cyanide from cyanide-containing wastewaters have been applied successfully (Dubey and Holmes, 1995; Dash et al., 2009; Cabello et al., 2018; Luque-Almagro et al., 2018). Considering that cyanide inhibits aerobic respiration, microorganisms that grow with cyanide contain a cytochrome *bd*-type cyanide-insensitive alternative oxidase (Jünemann, 1997). These “cyanotrophic” microorganisms also require a cyanide degradation route. In this sense, several pathways for cyanide degradation that include hydrolytic, oxidative or substitution/addition reactions have been described (Cabello et al., 2018; Luque-Almagro et al., 2018). However, most of these degradative pathways operate at neutral pH, while an alkaline pH is required to avoid the volatilization of cyanhydric acid. Finally, bacterial cyanide assimilation also requires defense mechanisms against the iron deprivation and the oxidative damage induced by cyanide (Luque-Almagro et al., 2007).

*Pseudomonas pseudoalcaligenes* CECT5344 is an alkaliphilic cyanotrophic bacterium that makes possible the bioremoval of cyanide at a very high rate. This strain was isolated from the Guadalquivir River (Córdoba, Spain) by enrichment cultivation with sodium cyanide, but it can also assimilate other cyano-derivative compounds, such as 3-cyanoalanine, cyanoacetamide, nitroferricyanide, cyanate and several metal-cyanide complexes (Luque-Almagro et al., 2005a, b, 2008, 2011a; Cabello et al., 2018; Sáez et al., 2019). The sequence of the whole genome of *P. pseudoalcaligenes* CECT5344 has been elucidated (Luque-Almagro et al., 2013; Wibberg et al., 2014, 2016), becoming a useful tool to develop transcriptomic and proteomic approaches. Thus, a DNA microarray analysis and a quantitative proteomic study by Liquid-Chromatography-Mass Spectrometry/Mass Spectrometry (LC-MS/MS) have been performed to analyze the global response of the strain CECT5344 to the cyanide and metals present in a residue generated by the jewelry industry (Luque-Almagro et al., 2015; Ibáñez et al., 2017). In this bacterium, the cyanide-insensitive cytochrome *bd*-type oxidase CioAB is required for respiration in the presence of cyanide (Quesada et al., 2007). Additionally, the cyanide assimilation pathway in the strain CECT5344 involves the production of oxaloacetate, which reacts chemically with cyanide to produce a 2-hydroxynitrile that is converted into ammonium through the nitrilase NitC (Luque-Almagro et al., 2011b; Estepa et al., 2012).

The response to cyanide in *P. pseudoalcaligenes* CECT5344 also includes the overproduction of proteins related to oxidative stress defense and iron homeostasis like the alkyl hydroperoxide reductase AhpC1 and the dihydrodipicolinate synthase DapA1, among other proteins (Luque-Almagro et al., 2007, 2015; Ibáñez et al., 2017). Under iron deficiency, some bacteria produce siderophores that allow iron binding and transport inside the cells. However, genome analysis reveals that *P. pseudoalcaligenes* CECT5344 is not able to produce siderophores, and therefore, other iron homeostasis mechanisms like the synthesis of chelators that facilitate the recycling of ferrous iron released from damaged iron-sulfur centers may be present this strain. In this regard, the DapA1 protein catalyzes the condensation of L-aspartate-4-semialdehyde with pyruvate to form 4-hydroxy-2,3,4,5-tetrahydrodipicolinate, an intermediate that is either converted into 2,3-dihydrodipicolinate in the lysine biosynthesis pathway or used to synthesize dipicolinic acid (McClintock et al., 2018; Gordon et al., 2019), a multi-functional compound that has been described as a highly effective metal chelator (Fernández-Pol, 1978; Maringanti and Imlay, 1999; Djaman et al., 2004; Pearce et al., 2017).

In this work, we have investigated the relationship between cyanide assimilation and iron homeostasis in the alkaliphilic bacterium *P. pseudoalcaligenes* CECT5344. For this purpose, a dihydrodipicolinate synthase defective *dapA1*^–^ mutant strain has been generated, physiologically characterized and subjected to a differential quantitative proteomic analysis by LC-MS/MS compared to the wild-type strain in media with the cyanide-containing wastewater from the jewelry industry.

## Results and Discussion

### Phylogenetic Analysis of DapA1 Protein

The first committed step of the diaminopimelate pathway involved in the biosynthesis of lysine in bacteria, archaea and plants is catalyzed by the enzyme dihydrodipicolinate synthase (Pearce et al., 2017). *P. pseudoalcaligenes* CECT5344 possesses a putative dihydrodipicolinate synthase (4-hydroxy-tetrahydrodipicolinate synthase) encoded by the *dapA1* gene (BN5_1907). This gene is located in the *cioAB-dapA1-nit4 locus* (BN5_1902-BN5_1912), which also code for the alternative cyanide-insensitive oxidase CioAB (W6R254 and W6QVH5) and the nitrilase Nit4 (W6R265). The induction of the *P. pseudoalcaligenes* CECT5344 *dapA1* gene in response to the cyanide-containing jewelry residue detected by DNA microarrays (Luque-Almagro et al., 2015), and the overproduction of the DapA1 protein (W6R260) in cells grown with this residue identified by a quantitative proteomic analysis (Ibáñez et al., 2017), have highlighted a possible role of DapA1 in cyanide assimilation and/or resistance. This function could be related with iron homeostasis considering that dipicolinates have been described as excellent metal chelators that bind ferrous iron with greater avidity than citrate, glutathione and other potential iron-binding cellular compounds (Maringanti and Imlay, 1999).

The strain CECT5344 presents a homologous DapA3 protein that shares 53% identity with DapA1. However, neither the *dapA3* gene (BN5_2718) nor its encoded product (W6R4K8) were found induced or overproduced by the cyanide-containing jewelry residue in the transcriptomic and proteomic studies previously carried out in *P. pseudoalcaligenes* CECT5344 (Luque-Almagro et al., 2015; Ibáñez et al., 2017). A phylogenetic analysis revealed that the concurrence of the DapA1 protein, the terminal cyanide-insensitive oxidase CioAB and the nitrilase NitC essential for cyanide assimilation is infrequent within bacterial strains. Thus, only 20 positive results were obtained from 1000 entries for each protein in a Protein Blast analysis, considering only those displaying identities higher than 60%. Most of the bacterial strains that contain DapA1, CioAB and NitC belonged to γ-proteobacteria, mainly from the *Pseudomonadaceae* family, but there were also some α-proteobacteria like *Sphingobium*, and several β-proteobacteria like *Collimonas* and members of the *Burkholderiaceae* family (Supplementary Figure S1).

### Physiological Characterization of the *dapA1*^–^ Mutant of *P. pseudoalcaligenes* CECT5344

**FIGURE 1:**
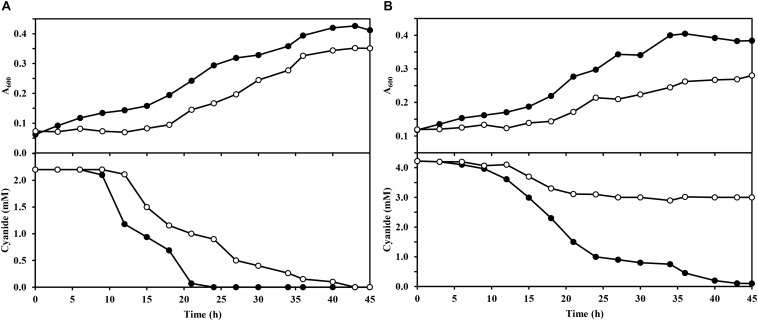
Growth of wild-type and *dapA1*^–^ strains of *P. pseudoalcaligenes* CECT5344 with the jewelry wastewater. Cultures of the wild-type strain (filled circles) and the *dapA1*^–^ mutant (open circles) were carried out in M9 media with 50 mM sodium acetate as carbon source and the cyanide-containing jewelry residue, either with 2 mM **(A)** or 4 mM **(B)** free cyanide, as the sole nitrogen source. Bacterial growth was determined by following the absorbance at 600 nm (A_600_) and cyanide consumption was determined colorimetrically as described in the Experimental Procedures section. Data correspond to a representative experiment of three independent replicates yielding similar results with standard deviations lower than 10%.

In this work, a *dapA1*^–^ mutant of *P. pseudoalcaligenes* CET5344 has been constructed by insertion of a gentamicin resistance cassette into the *dapA1* gene. Initially, the wild-type and *dapA1*^–^ strains were grown in M9 minimal medium with 50 mM acetate as carbon source and either ammonium chloride (2 mM) or the cyanide present in the jewelry residue (2 mM or 4 mM free cyanide) as the sole nitrogen source. Significant differences were not observed in the bacterial growth when compared the wild-type and *dapA1*^–^ strains in media with 2 mM ammonium (maximal A_600_ in the wild-type strain was 0.428 and in the *dapA1*^–^ mutant was 0.435). However, when the cyanide-containing jewelry residue was used as nitrogen source, differences in growth and cyanide consumption were observed between these strains (Figure 1). Thus, in the residue with 2 mM free cyanide, the *dapA1*^–^ mutant showed a delayed growth compared to the wild-type strain, and the cyanide uptake rate was slower than in the wild-type strain, although at the stationary growth phase both strains reached similar growth, and cyanide was totally consumed (Figure 1A). Nevertheless, when the jewelry residue was used at a free cyanide concentration of 4 mM, the *dapA1*^–^ mutant strain was severely affected in growth and cyanide uptake, being unable to consume completely the cyanide present in the media, in contrast to the wild-type strain (Figure 1B). These results indicate that DapA1 is not essential but required for an optimal biodegradation of the cyanide-containing jewelry residue in *P. pseudoalcaligenes* CECT5344, especially at a high concentration of cyanide and metals. It is worth noting that most bacteria contain a unique *dapA* gene that results essential for growth. However, the strain CECT5344 harbors an additional *dapA3* gene that may be involved in the synthesis of lysine, but it does not fulfill completely the function of the *dapA1* gene during cyanide assimilation. Other organisms also contain several *dapA* genes, like plants that usually have two *dapA* genes or the bacterium *Agrobacterium tumefaciens*, which contains up to 10 copies of this gene (Desbois et al., 2018).

### Intracellular Levels of Iron, Copper and Zinc in the Wild-Type and *dapA1*^–^ Mutant of *P. pseudoalcaligenes* CECT5344

Iron, copper and zinc are essential micronutrients for organisms. These metals are present as cofactors in many enzymes because their redox properties are excellent. However, organisms have to maintain a strict control of the intracellular metal levels to cover the requirements of the cells avoiding toxicity. Thus, under iron-deficient conditions, the ferric uptake regulator (Fur) induces the expression of different systems involved in iron uptake, storage and metabolism (Bereswill et al., 2000; Ollinger et al., 2006). However, the ferrous iron needs to be trafficked through the cell as a chelate, bound to a metabolite or a protein, to minimize oxidative stress and adverse reactions (Djaman et al., 2004). In this regard, dipicolinates are dicarboxylic compounds that show an excellent ability to chelate metals, both *in vivo* and *in vitro* (Fernández-Pol, 1978; Maringanti and Imlay, 1999; Pearce et al., 2017; Gordon et al., 2019). Additionally, crosstalk between metals has been described in bacteria. For example, in *Enterococcus faecalis* a deficiency or excess of iron provokes activation of different regulators, such as LexA and CopY, which are also stimulated by copper and zinc treatments (Latorre et al., 2018).

The jewelry industry wastewaters contain high amounts of iron, copper, and zinc, in addition to free cyanide and metal-cyanide complexes (Ibáñez et al., 2017). To elucidate a possible role of the DapA1 protein in metal homeostasis during assimilation of cyanide from this industrial residue, the intracellular content of iron, copper and zinc was determined by Inductively Coupled Plasma-Mass Spectrometry (ICP-MS) in cytoplasmic fractions from the *P. pseudoalcaligenes* CECT5344 wild-type strain and the *dapA1*^–^ mutant grown either with ammonium or the jewelry wastewater as the sole nitrogen source. The wild-type strain of *P. pseudoalcaligenes* CECT5344 showed the highest intracellular metal concentrations in cells grown with the jewelry residue, when compared to cells grown with ammonium. The metal content was also higher in the *dapA1*^–^ mutant grown with the jewelry residue than in cells grown with ammonium (Table 1). Even more interestingly, the intracellular iron concentration was higher in the *dapA1*^–^ mutant than in the wild-type strain, independently of the nitrogen source, and this increase was especially relevant in the cells grown with the jewelry wastewater. Similar results were also observed for the metals copper and zinc (Table 1).

**TABLE 1:** Determination of intracellular concentration of iron, copper and zinc.

**Strain/Nitrogen source**	Metal concentration (μg/mg CDW*)		
	
	**Fe**	**Cu**	**Zn**
Wild-type/Jewelry residue	9.23 ± 0.76	0.29 ± 0.03	19.55 ± 0.28
*dapA1*^–^/Jewelry residue	16.71 ± 1.18	0.72 ± 0.08	35.09 ± 1.25
Wild-type/Ammonium	4.11 ± 0.27	0.06 ± 0.02	0.48 ± 0.00
*dapA1*^–^/Ammonium	5.80 ± 0.72	0.16 ± 0.02	0.18 ± 0.00

(*) CDW, cell dry weight.

A high concentration of iron can be toxic to organisms because it contributes to the generation of oxidative stress. In the presence of iron, reactive oxygen species (ROS) like hydroxyl radical (^⋅^OH), anion superoxide radical (O_2_^⋅–^) and hydrogen peroxide (H_2_O_2_) are produced through the Fenton and Haber-Weiss reactions. These ROS can oxidize lipids, proteins and DNA, and therefore, cells must eliminate them to avoid significant damages. Oxidative stress derived from the elevated levels of intracellular iron in the *dapA1*^–^ mutant of *P. pseudoalcaligenes* CECT5344 could explain the growth inhibition observed in media with the cyanide-containing jewelry residue (Figure 1). In addition, cyanide may dissemble the Fe-S clusters of metalloproteins, and iron released from these damaged centers must be chelated to avoid toxicity and ROS formation. Dipicolinates efficiently bind iron in the ferrous form, and maintain this metal in its reduced state, protecting against oxidative stress and contributing to Fe-S cluster recycling for enzyme repairing processes (Maringanti and Imlay, 1999; Djaman et al., 2004). Therefore, the *dapA1*^–^ mutant could be affected in iron recycling, and this may explain the inhibition of cyanide assimilation observed in this mutant strain (Figure 1).

### Differential Quantitative Proteomic Analysis of the Wild-Type and *dapA1*^–^ Mutant of *P. pseudoalcaligenes* CECT5344

DNA microarray and quantitative proteomic analyses have been previously performed to characterize the global response of *P. pseudoalcaligenes* CECT5344 to the jewelry residue (Luque-Almagro et al., 2015; Ibáñez et al., 2017). In this work, to analyze the *dapA1*^–^ mutant at the proteomic level, this mutant and the wild-type strain were grown per triplicate (three biological replicates of each strain) with ammonium chloride (2 mM) or the jewelry residue (2 mM free cyanide) as the sole nitrogen source, and the samples were subjected to LC-MS/MS analysis.

In the proteomic study carried out in this work, a total of 1691 proteins were identified in the wild-type strain of *P. pseudoalcaligenes* CECT5344 cultured with ammonium, whereas 1570 proteins were identified in the presence of the jewelry residue. The differential analysis between these two nitrogen sources in the wild-type strain yielded similar results to those previously published (Ibáñez et al., 2017). In the study with the *dapA1*^–^ mutant of *P. pseudoalcaligenes* CECT5344, 2022 proteins were identified in the cells grown with ammonium, and 2035 proteins were detected in the cells cultured with the jewelry residue.

Significant differences were observed when the analysis was performed comparing the proteomic profiles of the wild-type and *dapA1*^–^ mutant cells cultured with the jewelry residue. In this differential proteomic analysis the wild-type was used as the reference strain, and therefore proteins over-represented in the wild-type strain (down-represented in the *dapA1*^–^ mutant) showed a positive fold change, while proteins over-represented in the mutant strain (down-represented in the wild-type strain) had a negative fold change. Those proteins displaying a fold change higher than 40 were considered “exclusive” of the wild-type strain, while proteins with a fold change lower than −146 were defined as “exclusive” of the *dapA1*^–^ mutant. With these criteria, a total of 124 proteins were found over-represented in the wild-type strain (or down-represented in the *dapA1*^–^ mutant), whereas 165 proteins were down-represented in the wild-type strain (or over-represented in the *dapA1*^–^ mutant). Also, a total of 26 proteins were exclusive of the wild-type strain, whereas 399 proteins were exclusive of the *dapA1*^–^ mutant (Supplementary Table S1).

Breaking down the proteome into gene ontology (GO) groups reveals insights into functional categories of proteins that are either enriched (over-represented) or suppressed (down-represented) under cyanotrophic conditions (Supplementary Figure S2). Highly represented GO groups in the wild-type strain included “arginine catabolic process to succinate” and “protein repair,” among others (Supplementary Figure S2A). The differential study of the wild-type strain *versus* the *dapA1*^–^ mutant in the cyanide-containing jewelry residue showed that several GO groups resulted over-represented as consequence of the mutation in the *dapA1* gene, such as “arginine biosynthetic process,” “tricarboxylic acid cycle” “DNA recombination” and “DNA repair,” among others (Supplementary Figure S2B).

**FIGURE 2:**
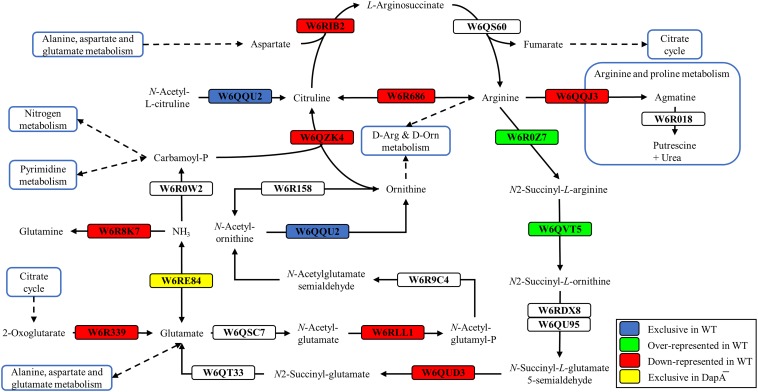
Arginine and ornithine biosynthetic pathways of *P. pseudoalcaligenes* CECT5344. Significative changes related to arginine and ornithine metabolism in the differential proteomic analysis of the wild-type and *dapA1*^–^ strains of *P. pseudoalcaligenes* CECT5344 cultured with the jewelry residue as sole nitrogen source are shown. Pathways were draw according to KEGG mapper (ppse00220) and SBGN (Le Novère et al., 2009). Color code: blue, proteins exclusive of the wild-type strain; green, proteins over-represented in the wild-type strain; red, proteins down-represented in the wild-type strain; yellow, proteins exclusive of the *dapA1*^–^ mutant.

**FIGURE 3:**
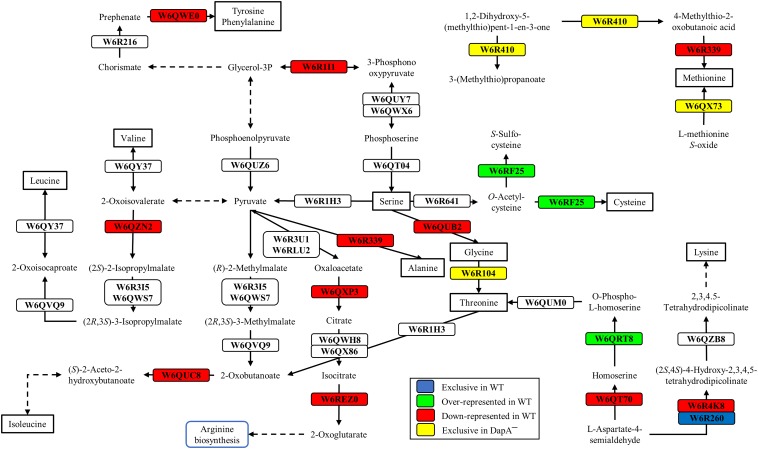
Biosynthesis of several amino acids in *P. pseudoalcaligenes* CECT5344. Significative changes related to the biosynthesis of different amino acids in the differential proteomic analysis of the wild-type and *dapA1*^–^ strains of *P. pseudoalcaligenes* CECT5344 cultured with the jewelry residue as sole nitrogen source are shown. Pathways were draw according to KEGG mapper (ppse01230 ppse00270, ppse00330 and ppse00220) and SBGN (Le Novère et al., 2009). Color code: blue, proteins exclusive of the wild-type strain; green, proteins over-represented in the wild-type strain; red, proteins down-represented in the wild-type strain; yellow, proteins exclusive of the *dapA1*^–^ mutant.

Proteins exclusive of the wild-type strain were the acetylornithine deacetylase (W6QQU2), the allophanate hydrolase (W6QRS4), a hydrogen peroxide-inducible activator (W6R3L3), and a LuxR family transcriptional regulator (W6R7G4), among others (Figure 2 and Supplementary Table S1). Another protein exclusive of the wild-type strain of *P. pseudoalcaligenes* CECT5344 was the dihydrodipicolinate synthase DapA1 (W6R260) (Figure 3), which is the expected consequence of the disruption of the *dapA1* gene by mutagenesis. However, a small portion of the 5′-end of the *dapA1* gene resulted unaffected by the mutagenesis and some peptides corresponding to the N-terminal end of the DapA1 protein could be identified in the mutant (Supplementary Table S1). In any case, this small *dapA1* gene fragment was not large enough to code for a functional protein, and therefore the complete DapA1 protein was not detected in the *dapA1*^–^ mutant.

**FIGURE 4:**
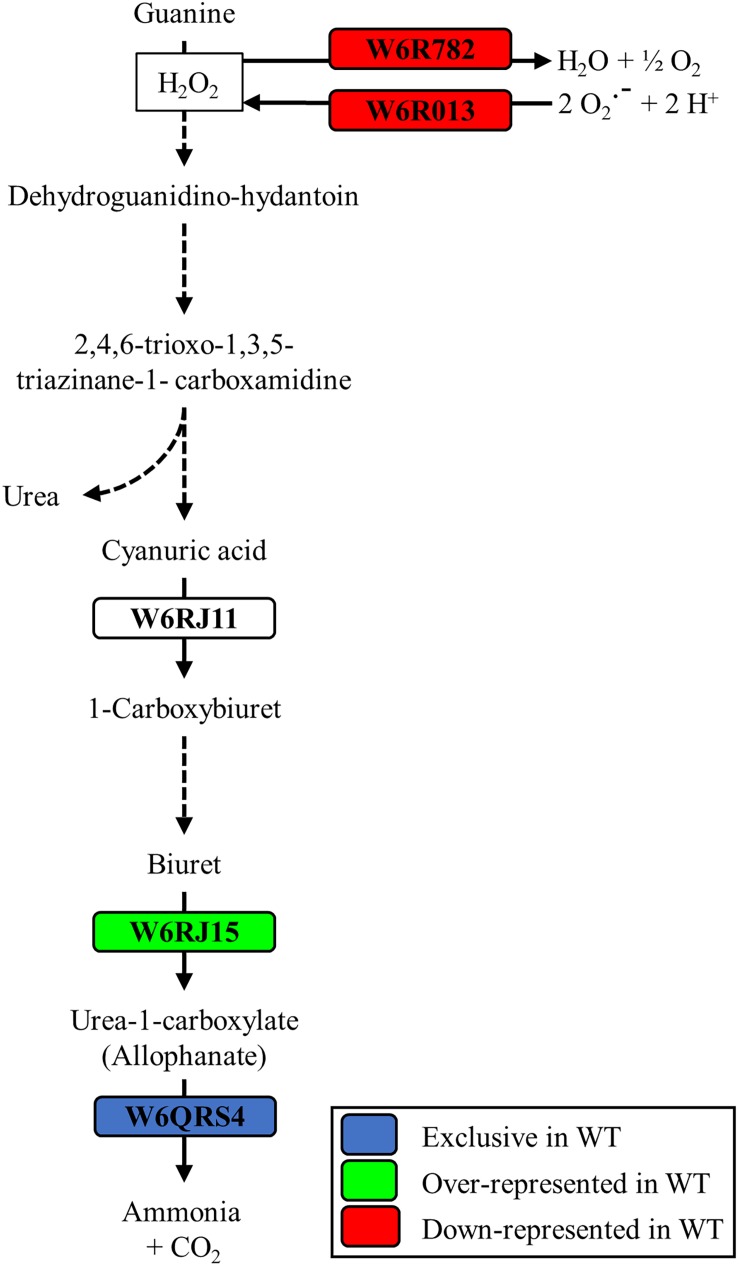
Putative role of the allophanate hydrolase of *P. pseudoalcaligenes* CECT5344 in cyanuric acid degradation. The proteomic analysis of the wild-type and *dapA1*^–^ strains was carried out as described in the Experimental Procedures section. The formation of cyanuric acid during chemical oxidation of guanine by hydrogen peroxide, and further degradation of cyanuric acid to ammonium through allophanate hydrolase is shown. Color code: blue, proteins exclusive of the wild-type strain; green, proteins over-represented in the wild-type strain; red, proteins down-represented in the wild-type strain.

Highly represented in the wild-type strain was the carbon storage regulator Csr (W6RDY8, fold change 13.56). This regulatory protein has counterparts in many microorganisms, such as *E. coli*, where it exerts a nutrient specific control over central metabolism (Revelles et al., 2013). Concerning to nitrogen metabolism, over-represented proteins in the *P. pseudoalcaligenes* CECT5344 wild-type strain were the assimilatory nitrate reductase (W6R2U2, fold change 3.48), the catalytic subunit and the small subunit of the assimilatory nitrite reductase (W6RFM9 and W6QUV5, with fold changes 3.10 and 2.77, respectively), the urease accessory protein UreG (W6RBC9, fold change 2.26) and the cyanase CynS (W6QY14, fold change 2.17) (Supplementary Table S1). This result highlights that the *dapA1*^–^ mutant of *P. pseudoalcaligenes* CECT5344 is probably less efficient in using alternative nitrogen sources than the wild-type strain. As mentioned, cyanide damages the iron-sulfur centers, which are present in the enzymes involved in nitrate/nitrite assimilation, and as consequence of the *dapA1* mutation iron is not efficiently used for rebuilding the iron-sulfur clusters of these metalloproteins. Other over-represented proteins in the wild-type strain were the arginine *N*-succinyltransferase (W6R0Z7, fold change 2.33) and the *N*-succinylarginine dihydrolase (W6QVT5, fold change 2.01). These two enzymes participate in the urea cycle and arginine metabolism, as well as the acetylornithine deacetylase (W6QQU2, Figure 2) and the allophanate hydrolase (W6QRS4, Figure 4), which were exclusive of the wild-type strain. Allophanate hydrolase is conserved in many organisms and converts allophanate to ammonium and carbon dioxide, allowing the utilization of urea as nitrogen source. It has been also proposed that allophanate hydrolase participates in the degradation pathway of the herbicide *S*-triazine by soil bacteria (Fan et al., 2013). *P. pseudoalcaligenes* CECT5344 does not contain the enzymes that degrade atrazine producing cyanuric acid, but it presents the metabolic pathway to degrade cyanuric acid to ammonium and carbon dioxide (Figure 4). In fact, the cyanuric acid amidohydrolase (W6RJ11) was found exclusive of the cells grown in the jewelry wastewater in a previous proteomic study carried out in the wild-type strain of *P. pseudoalcaligenes* CECT5344 (Ibáñez et al., 2017). It has been described that cyanuric acid is also produced from guanine after DNA lesions caused by oxidative stress. In particular, hydrogen peroxide may oxidize guanine forming a carboxamidine derivative, which is a precursor of cyanuric acid (Irvoas et al., 2014).

Several enzymes related with amino acid biosynthesis were also enriched in the wild-type strain, such as the homoserine kinase (W6QRT8) that produces *o-*phospho-L-homoserine for threonine biosynthesis and the cysteine synthase CysM3 (W6RF25) that converts *o*-acetylcysteine into cysteine (Figure 3). The *cysM3* gene (BN5_1910) clusters together with the *cioAB* genes (BN5_1902 and BN5_1903, respectively). In addition, two proteins of unknown function encoded by the *nit1C* gene cluster, the *S*-adenosyl methionine domain-containing protein NitD (H9N5E3, fold change 2.29) and the FAD-dependent oxidoreductase NitH (H9N5D8, fold change 2.76), were also over-represented in the wild-type strain (Supplementary Table S1).

On the other hand, an exclusive protein of the *dapA1*^–^ mutant was NfuA (W6RFE4, Supplementary Information, Supplementary Table S1). This protein has been described to be involved in iron-sulfur cluster biogenesis under severe conditions, such as iron starvation or oxidative stress. NfuA binds a [4Fe-4S] cluster and transfers this center to apoproteins, thereby participating in the maturation of Fe/S-containing proteins. Also, it has been postulated that NfuA could act as a scaffold/chaperone for damaged iron-sulfur proteins (Angelini et al., 2008). In the case of the *dapA1*^–^ mutant, the deficiency in the synthesis of dipicolinates that may act as iron chelators, together with the presence of cyanide that causes damage of the iron-sulfur clusters, may explain the increased levels of iron inside the cells (Table 1) and the over-production of the NfuA protein to facilitate recycling of damaged Fe-S clusters in this mutant.

Another relevant over-represented protein in the *dapA1*^–^ mutant was the ferric uptake regulator Fur (W6QZB3, fold change −3.37). This result also supports that the *dapA* gene disruption affects the general iron homeostasis in the cells, resulting in the induction of the Fur regulatory protein and a high intracellular iron concentration. In bacteria, iron metabolism is controlled in response to iron availability through the Fur protein, which regulates the iron-dependent expression of many genes (Andrews et al., 2003). In addition, over-represented in the *dapA1*^–^ mutant was the copper-resistance protein CopA (W6QVZ2, fold change −2.54), which could be also regulated by Fur (Supplementary Table S1). Induction of the CopA protein may be also related to the increased intracellular levels of copper in the *dapA1*^–^ mutant (Table 1). Additional proteins exclusive of the *dapA1*^–^ strain were CRISPR-associated Cse3 family proteins (W6QS91, W6QS95, W6QTT9, W6QR33, and W6QZ01), the helicase/nuclease RecB protein (W6QZA1) involved in DNA repair, the multidrug resistance protein MdtC (W6R972), the outer-membrane TonB-dependent siderophore receptor FhuA (W6R3L4) and the high-affinity zinc uptake protein ZnuA (W6R3V4), which could lead to the increased levels of iron and zinc observed in the *dapA1*^–^ mutant (Table 1). It has been described that Fur could regulate multidrug resistance proteins and DNA recombination events (Grifantini et al., 2003). Siderophores are low molecular weight compounds that specifically chelate the ferric ion, making this metal available to the cell (Neilands, 1995). Fe^3+^-siderophores are incorporated into the cells by ABC-type ATP-dependent transporters or by TonB-dependent systems (Crosa and Walsh, 2002). Considering that the presence of cyanide may cause iron deficiency due to its capability to bind this metal, the induction of a siderophore transport system by cyanide may be expected. However, efforts to identify siderophore molecules in the strain CECT5344 in response to cyanide have been unsuccessful up to date. Genome analysis also revealed that this strain lacks the capability to produce siderophores, and therefore, other mechanisms like the synthesis of dipicolinates as iron chelators may be involved in iron homeostasis during cyanide assimilation.

**FIGURE 5:**
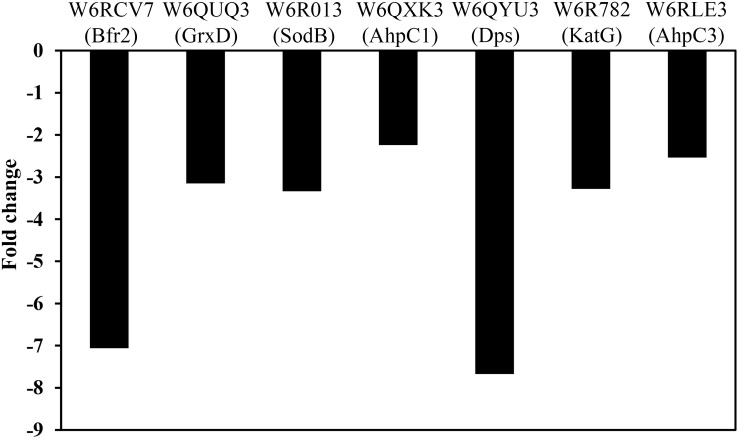
Effect of the *dapA1* gene mutation on the normalized protein intensity of some proteins related to oxidative-stress and iron homeostasis in *P. pseudoalcaligenes* CECT5344. The proteomic analysis of the wild-type and *dapA1*^–^ cells was carried out as described in the Experimental Procedures section. The wild-type strain has been used as reference, and therefore over-represented proteins in the *dapA1*^–^ mutant showed negative fold changes. Represented proteins: bacterioferritin Bfr2 (W6RCV7), glutaredoxin GrxD (W6QUQ3), superoxide dismutase SodB (W6R013), alkyl hydroperoxide reductase AhpC1 (W6QXK3), ferritin Dps family protein (W6QYU3), catalase-peroxidase KatG (W6R782) and alkyl hydroperoxide reductase AhpC3 (W6RLE3).

In *E. coli*, it has been demonstrated that Fur regulates the expression of genes involved in the response against ROS, and that accumulation of dipicolinates also triggers derepression of the Fur regulon increasing the intracellular pool of iron. This metal is chelated and stabilized by dipicolinates, protecting against superoxide dismutase deficiency (Maringanti and Imlay, 1999; Grifantini et al., 2003; Djaman et al., 2004). In this context, in the *P. pseudoalcaligenes dapA1*^–^ mutant were over-represented different proteins related to the ROS response caused by the high levels of iron, including the superoxide dismutase SodB (W6R013, fold change −3.34), the catalase-peroxidase KatG (W6R782, fold change −3.31), the alkyl hydroperoxide reductases AhpC1 and AhpC3 (W6QXK3 and W6RLE3, fold changes −2.26 and −2.60, respectively), the glutaredoxin GrxD (W6QUQ3, fold change −3.15), the ferritin Dps family protein (W6QYU3, fold change −7.84) and the bacterioferritin Bfr2 (W6RCV7, fold change −7.97) (Figure 5). This proteomic response of the *dapA1*^–^ mutant could alleviate the negative effects of dipicolinate deficiency on oxidative stress and iron recycling during cyanide assimilation.

Several enzymes that were over-represented in the *dapA1*^–^ mutant are involved in arginine metabolism and the urea cycle, such as arginine deiminase (W6R686, fold change −6.26), arginine decarboxylase (W6QQJ3, fold change −2.21), ornithine carbamoyltransferase (W6QZK4, fold change −2.24) and arginosuccinate synthase (W6RIB2, fold change −2.35), and also in the biosynthesis of other amino acids like methionine and threonine (Figure 3). Curiously, the ribonucleoside-diphosphate reductase that transforms ribonucleoside-5′-diphosphate into deoxyribonucleoside 5′-diphosphate was also over-represented in the *dapA1*^–^ mutant (W6R0E8, fold change −8.81) (Supplementary Table S1). This enzyme contains a diferric iron center and catalyzes a reductive reaction that is initiated with the generation of a free radical. The enzyme requires electrons donated by the dithiol groups of the protein thioredoxin, and regeneration of thioredoxin occurs when NADPH provides two hydrogen atoms that are used to reduce the disulfide group of thioredoxin.

As mentioned above, the *dapA1*^–^ mutant of *P. pseudoalcaligenes* was unable to assimilate cyanide at a similar rate to the wild-type (Figure 1). This is also consistent with the induction in the *dapA1*^–^ mutant of the nitrogen regulatory protein PII (W6QQL3, fold change −4.55) that responds to nitrogen starvation conditions, and the enzyme glutamine synthetase (W6R8K7, fold change −5.91), which is under the control of the PII protein (Figure 2). Interestingly, the dihydrodipicolinate synthase DapA3 (W6R4K8), a homologous protein to DapA1, was also over-represented (fold change −3.25) in the *P. pseudoalcaligenes dapA1*^–^ mutant (Supplementary Table S1). This is probably a mechanism to compensate the mutation in the *dapA1* gene, but this compensation was not completely effective according to the phenotype of the *dapA1*^–^ mutant.

Finally, a differential study was performed comparing the proteomic profiles of the wild-type strain and the *dapA1*^–^ mutant cultured with ammonium as sole nitrogen source. In this proteomic analysis, 10 proteins were found exclusive of the wild-type strain, whereas 255 proteins were exclusive of the *dapA1*^–^ mutant. In addition, 33 proteins were over-represented in the wild-type strain and 93 proteins were over-represented in the *dapA1*^–^ mutant (Supplementary Table S2). When cultured with ammonium, the *dapA1*^–^ mutant also displayed increased levels of intracellular iron compared to the wild-type strain, although the highest levels of intracellular iron in the *dapA1*^–^ mutant were observed in the presence of the cyanide-containing jewelry residue (Table 1). Under this condition (ammonium as nitrogen source), CRISPR/Cas proteins (W6QS95, W6QTT9, W6QR33), the glutathione *S*-transferase (W6QT22), a citrate transporter (W6RB28), and the aliphatic nitrilase Nit1 (W6RF39) were exclusively found in the *dapA1*^–^ mutant. Additionally, over-represented in the *dapA1*^–^ mutant cultured with ammonium were the Fur regulator (W6QZB3, fold change −2.69), the ferritin Dps family protein (W6QYU3, fold change −4.26), the catalase-peroxidase KatG (W6R782, fold change −2.23), the superoxide dismutase SodB (W6R013, fold change −2.39), the nitrilase Nit4 (W6R265, fold change −10.40) and the malic enzyme (W6QUA1, fold change −23.40). These results indicated that many proteins involved in the response mediated by Fur are also over-expressed in the *dapA1*^–^ mutant of *P. pseudoalcaligenes* CECT5344 when the cells were grown with ammonium as the sole nitrogen source. Curiously, several proteins over-represented in the *dapA1*^–^ mutant grown in ammonium were also over-represented in the wild-type cells grown with the cyanide-containing jewelry wastewater (Ibáñez et al., 2017), probably because both strains cultured with these different nitrogen sources accumulated similar intracellular levels of iron (Table 1). Among these proteins were included a citrate transporter (W6RB28) and the nitrilases Nit1 (W6RF39) and Nit4 (W6R265), which are encoded by genes located close to the *dapA1* gene. Up to date, the nitrilases Nit1 and Nit4 have no known function in the metabolism of cyanide in the strain CECT5344, but the citrate transporter could participate in the entrance of iron to the cell, because citrate has been described as a siderophore (Guerinot et al., 1990). Furthermore, the aliphatic nitrilase Nit1 and the citrate transporter have been predicted as putative targets of small RNAs (sRNA601 and sRNA649, respectively) in the wild-type strain grown with the jewelry residue (Olaya-Abril et al., 2019).

**FIGURE 6:**
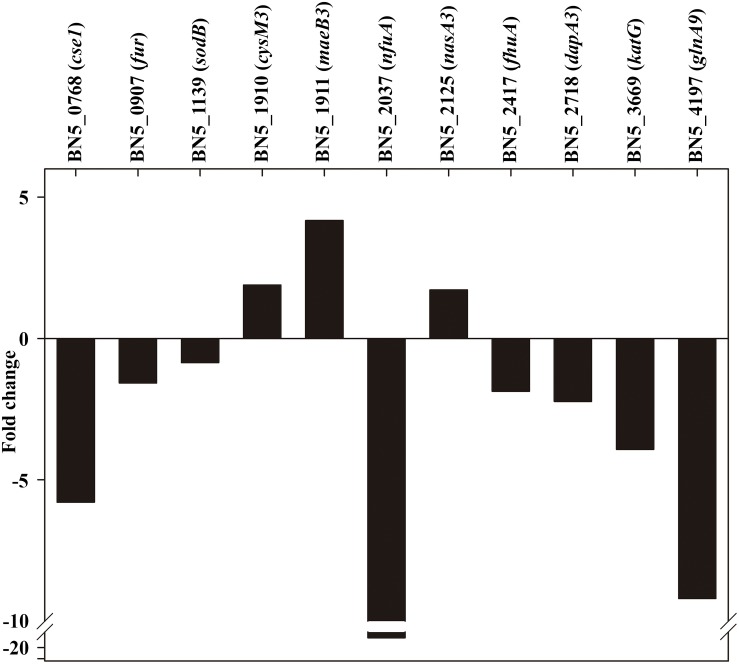
qRT-PCR analysis of several genes affected by the *P. pseudoalcaligenes* CECT5344 *dapA1* gene mutation. Transcriptional analysis by qRT-PCR of relevant genes differentially expressed in the wild-type strain and the *dapA1*^–^ mutant cells grown with the jewelry residue was carried out as described in the Experimental Procedures section. The wild-type strain has been used as reference, and therefore genes over-expressed in the *dapA1*^–^ mutant showed negative fold changes while genes over-expressed in the wild-tipe strain displayed positive fold changes. Represented genes: CRISPR-associated *cse1* family gene (BN5_0768), ferric uptake regulator *fur* (BN5_0907), superoxide dismutase *sodB* (BN5_1139), cysteine synthase *cysM3* gene (BN5_1910), NADP-dependent malic enzyme-encoding *maeB3* gene (BN5_1911), Fe-S biogenesis *nfuA* gene (BN5_2037), nitrate reductase *nasA3* gene (BN5_2125), TonB-dependent siderophore receptor *fhuA* gene (BN5_2417), dihydrodipicolinate synthase *dapA3* gene (BN5_2718), catalase-peroxidase *katG* gene (BN5_3669), and glutamine synthetase *glnA9* gene (BN5_4197).

To validate the quantitative proteomic studies presented in this work, a qRT-PCR analysis has been performed. For this purpose, RNA was isolated from the wild-type strain and the *dapA1*^–^ mutant grown with ammonium or the cyanide-containing jewelry residue, and relevant genes encoding proteins found differentially expressed in the LC-MS/MS analysis were amplified using specific primers. For all these selected genes, the transcriptomic and protein patterns correlated well. As shown in Figure 6, the expression of genes like *cysM3*, *maeB3*, and *nasA3* that code for proteins over-repressented in the wild-type strain decreased in the *dapA1*^–^ mutant, as indicated by their positive fold changes. Conversely, an increased expression of genes coding for proteins over-represented in this mutant strain (indicated by negative fold-changes) was also observed (Figure 6).

## Conclusion

This study has highlighted that the dihydrodipicolinate synthase DapA1 of *P. pseudoalcaligenes* CECT5344 is relevant for the tolerance to cyanide and metals during degradation of jewelry industry wastewaters. Proteomic analysis and phenotypic characterization of a *dapA1*^–^ mutant have revealed the importance of iron homeostasis and oxidative stress during assimilation of the cyanide present in the jewelry residue. The *dapA1*^–^ mutant accumulated higher concentrations of iron and other metals than the wild-type strain and over-produced different proteins related to amino acids biosynthesis, oxidative stress defense and iron storage and homeostasis, including the ferric-uptake regulator Fur. The DapA1 protein is involved in the production of dipicolinates, which bind and stabilize ferrous iron protecting against oxidative stress and allowing rebuilding of damaged Fe-S clusters, as previously reported in *E. coli*. This could explain the negative effect of the *dapA1* gene disruption on the assimilation of the cyanide present in the jewelry wastewater.

## Experimental Procedures

### Chemicals

The cyanide-containing residue from the jewelry industry was kindly supplied by GEMASUR (Córdoba, Spain). This jewelry wastewater displays a pH 13 and contains a total cyanide concentration 1.5 M (about 0.5 M cyanide bound to metals as stable complexes and 1 M as free cyanide). The most abundant metals in the jewelry residue were copper, zinc and iron (Ibáñez et al., 2017). When used as nitrogen source, the residue was diluted in the culture medium to give the desired concentration of free cyanide (2 or 4 mM). All other chemicals and reagents were of high purity and purchased from Sigma-Aldrich (St. Louis, MO, United States).

### Bacterial Strains, Media and Growth Conditions

The bacterial strains and plasmids used in this work, and their most relevant characteristics, are shown in Supplementary Table S3.

The wild type and *dapA1*^–^ mutant strains of *P. pseudoalcaligenes* CECT5344 were grown in M9 minimal medium (Sambrook et al., 1989), adjusted to pH 9.5, under aerobic conditions at 30°C and 220 rpm into an orbital shaker (Luque-Almagro et al., 2005b). Sodium acetate (50 mM) was used as carbon source and the nitrogen source was ammonium chloride or jewelry wastewater, at the concentrations indicated for each experiment.

*Escherichia coli* DH5α and S17.1 were grown in Luria-Bertani medium (Sambrook et al., 1989), which contained 10 g bactotryptone, 5 g yeast extract, 5 g NaCl and 1 L of distilled water. *E. coli* cells were grown under aerobic conditions at 37°C at 220 rpm into an orbital shaker.

When solid agar plates were required, 1.5% bacteriological agar was added to the liquid medium before sterilization. When indicated, nalidixic acid and gentamicin were added at 10 and 25 μg/mL, respectively.

### Determination of Bacterial Growth

Cell growth was determined by following the absorbance of the bacterial cultures at 600 nm (A_600_) in a spectrophotometer (Thermo Fisher Scientific, Waltham, MA, United States).

### Analytical Determinations

The concentration of ammonium was measured using the Nessler reagents (Morrison, 1971). Cyanide was determined colorimetrically by the Asmus and Garschagen (1953) method, as previously described (Luque-Almagro et al., 2005b). Intracellular content of iron, copper and zinc was determined by ICP-MS. Wild-type and *dapA1*^–^ mutant of *P. pseudoalcaligenes* CECT5344 were grown (100 mL cultures) with ammonium or the cyanide-containing jewelry residue (2 mM concentration) as the sole nitrogen source. When the cultures reached an absorbance at 600 nm of approximately 0.3 (about 60% of the nitrogen source consumed), cells were harvested and washed in a buffer solution with 20 mM Tris–HCl (pH 8) and 4 mM EDTA. The resulting pellets were dried at 80°C for 96 h and the cell dry weight was determined. Then, dry pellets were subjected to a digestion with high-purity nitric acid. Metal measurements were carried out in an ICP-MS equipment (PerkinElmer model Nexion 350X) located at the Central Service for Research Support (SCAI) of the University of Córdoba. Three biological samples were analyzed for each bacterial strain and condition (ammonium or residue). Statistical significance was analyzed by a two-tailed *t*-test analysis.

### Construction of the *dapA1*^–^ Mutant Strain of *P. pseudoalcaligenes* CECT5344

Total genomic DNA was isolated from *P. pseudoalcaligenes* CECT5433 wild-type strain using a commercial kit from Promega, following the instructions of the manufacturer. For the extraction of plasmid DNA, FavorPrep Plasmid Mini Extraction Kit (FAPDE300, FAVORGEN) was used according to the protocol supplied by the manufacturer. Digestions with restriction enzymes were carried out using the New England Biolabs reagents, and DNA fragments were run onto 1% agarose gel in TAE buffer (40 mM Tris–HCl, 20 mM acetic acid and 1 mM EDTA). When necessary, DNA fragments were cleaned by using the isolated II PCR and gel Kit (BIO-520559, Bioline). Ligation mixtures were carried out at 37°C, with 1 μL of T4 DNA Ligase (Promega).

To construct a *dapA1*^–^ mutant of *P. pseudoalcaligenes* CECT5344, the *dapA1* gene (BN5_1907) was amplified by PCR from genomic DNA using the primer pairs DapA1-F and DapA1-R (Supplementary Table S4) to generate a DNA fragment of 1812 bp that includes the *dapA1* gene. The PCR reaction contained, in addition to DNA and the indicated primers, 0.5 μL of DNA polymerase (Bioline), a mixture of deoxyribonucleotides and the buffer specified by the manufacturer. The PCR program included an initial step of denaturation at 96°C for 30 s, followed by 30 cycles of denaturation at 96°C for 30 s each, annealing at 65°C for 30 s each, and elongation at 72°C for 15 s each. The program was completed with a last step of 2 min for final elongation at 72°C. Restriction map was analyzed by using the Webcutter 2.0 software^1^. The amplified DNA fragment was ligated to the vector pGEM-T to generate the pDapA1-1 plasmid (Supplementary Table S1). The T7 forward primer (T7-F) was used to define the orientation of the cloned fragments. By using two *Eco*RI sites located in a central region of the *dapA1* gene, a gentamicin resistance cassette was inserted to generate the pDapA1-2 plasmid, resulting in a disrupted *dapA1* gene. The defective *dapA1* gene with the inserted antibiotic resistance cassette was extracted from pDapA1-2 plasmid using the restriction enzymes *Bam*HI and *Hind*III, and cloned into the suicide vector pK18mob to generate pDapA1-3 plasmid, which was introduced into *E. coli* S17.1. This strain was used as the donor strain and *P. pseudoalcaligenes* CECT5344 was used as the receptor strain in conjugational mattings to generate the *dapA1*^–^ mutant of *P. pseudoalcaligenes* CECT5344. Transconjugants were selected in solid LB medium with nalidixic acid and gentamicin.

### Quantitative Proteomic Analysis by Liquid Chromatography Coupled to Mass Spectrometry (LC-MS/MS)

The proteomic analysis was carried out with the wild-type strain and the *dapA1*^–^ mutant of *P. pseudoalcaligenes* CECT5344. Cells were cultured in M9 minimal medium with 50 mM acetate as carbon source and 2 mM ammonium or the jewelry residue (2 mM free cyanide) as the sole nitrogen source. When approximately 60% of the nitrogen source was consumed, cells were harvested by centrifugation, resuspended in 300 μL of lysis buffer containing 8 M urea, Tris–HCl (50 mM, pH 7.5) and 4% CHAPS (w/v), and disrupted by sonication (8 pulses of 20 s at 90 W). Samples were centrifuged at 13500 × *g* for 30 min at 4°C to remove cell debris and unbroken cells and the supernatants were precipitated using the commercial kit 2D-Clean Up (GE Healthcare) following the protocol provided by the manufacturer. Finally, the quantification of the protein concentration in the samples was carried out by the method of Bradford (1976). The samples were analyzed in the Central Service for Research Support (SCAI) of the University of Córdoba, as previously described (Olaya-Abril et al., 2018). Following an analysis by using MaxQuant (Cox and Mann, 2008), which uses the free available software Perseus (version 1.5.6.0)^2^, a differential analysis was carried out. Proteins identified from only one peptide and/or in only one replicate were discarded. Proteins identified in at least two replicates per each condition were used for differential pairwise comparison analysis if they were positive after considering a two-way Student-test (Olaya-Abril et al., 2018). Proteins were considered differentially expressed when the fold change was ≥2 with a *p* value <0.05. The mass spectrometry data has been deposited in the ProteomeXchange Consortium via the PRIDE partner repository prior to publication (Vizcaino et al., 2013) with the dataset identifier PXD014027. GO analysis was performed using the web application Comparative GO (Fruzangohar et al., 2015). For that, only those changes with a *p* value <0.05 after a hyper-geometric distribution [E(GO)] test of the third level of GO biological function were shown. The whole genome of *P. pseudoalcaligenes* CECT5344 was used as reference and the parameter [E(GOi)], which is used to determine the GO enrichment, is calculated by using the formula: [E(GOi)] = sample size/genome size x GOi. Integration of final proteomic data were performed by using the tool KEGG Mapper.

### Evolutionary Taxa Relationships

The evolutionary relationships were inferred using the UPGMA method (Sneath and Sokal, 1973). The evolutionary distances were computed using the Poisson correction method (Zuckerkandl and Pauling, 1965). The analysis involved 20 amino acid sequences for DapA, aligned by using ClustalW with the homolog proteins of species that also possess in their genomes putative genes coding for the alternative cyanide-insensitive oxidase CioAB and the nitrilase NitC. All positions containing gaps and missing data were eliminated. There was a total of 323 positions in the final dataset. Evolutionary analyses were conducted in MEGA7 (Kumar et al., 2016).

### RNA Quantitation by qRT-PCR

RNA isolation, cDNA synthesis and cDNA quantitation was carried out from the wild-type and *dapA1*^–^ cells grown with the cyanide-containing jewelry residue (2 mM free cyanide) as nitrogen source, as previously described (Olaya-Abril et al., 2018). Target cDNAs were amplified in three independent PCR reactions using specific primers (Supplementary Table S4). For relative quantitation of the fluorescence values, a calibration curve was performed using dilution series from 80 to 0.008 ng of *P. pseudoalcaligenes* CECT5344 genomic DNA sample. Data were normalized by using the *dnaN* gene as housekeeping and a *t*-test was applied. Samples with a positive *t*-test were shown as fold change using the wild-type strain as reference.

### Bioinformatics Analysis and Statistic

Primers were designed by using the Oligo 7 software^3^. Perseus version 1.5.6.0^2^ was used to obtain the differentially expressed proteins analysis. The web servers KEGG Mapper^4^ and Comparative GO^5^ were used to elucidate the biological processes affected. Growth curves and analytical determinations were carried out three times from independent experiments.

## Data Availability Statement

The datasets generated for this study can be found in the ProteomeXchange Consortium via the PRIDE partner repository with the dataset identifier PXD014027.

## Author Contributions

AO-A, VL-A, and MR performed the proteomic analysis. MP and DM carried out the bacterial growth curves. LS and PC performed the analytical determinations. AO-A performed the qRTPCR analysis. MR and CM-V conceptualized, administrated, and funded the project. MR and CM-V wrote the manuscript.

## Conflict of Interest

The authors declare that the research was conducted in the absence of any commercial or financial relationships that could be construed as a potential conflict of interest.

## Footnotes

1 http://heimanlab.com/cut2.html

2 http://www.coxdocs.org/doku.php?id=perseus:start

3 http://www.oligo.net/

4 https://www.genome.jp/kegg/tool/map_pathway2.html

5 http://www.comparativego.com/

## Supplementary Material

FIGURE S1 Evolutionary relationships of DapA taxa in bacterial strains containing in their genome genes coding for DapA, CioAB and NitC proteins MEGA7 was used to perform this analysis. The optimal tree resulted with a branch length of 2.51202227 is shown. The tree is drawn to scale, with branch lengths in the same units as those of the evolutionary distances used to infer the phylogenetic tree. Bootstrap values indicate the percentage in which a certain bipartition of the taxon set is present in 1000 bootstrap trees.

FIGURE S2 Gene ontology (GO) category analysis of proteins differentially affected in the wild-type strain and the *dapA1*^–^ mutant of *P. pseudoalcaligenes* CECT5344. **(A)** Significative changes in GO groups among proteins over-represented in the wild-type strain of *P. pseudoalcaligenes* CECT5344 grown with the jewelry residue (green). The genome of the wild-type strain was considered as reference (red). **(B)** Significative changes in GO groups among proteins down-represented in the wild-type strain of *P. pseudoalcaligenes* CECT5344 cultured with the jewelry residue (yellow). The genome of the wild-type strain was considered as reference (red).

TABLE S1 Quantitative proteomic analysis of the wild-type (reference) and DapA1^–^ mutant strains of *P. pseudoalcaligenes* in jewelry residue.

TABLE S2 Quantitative proteomic analysis of the wild-type (reference) and DapA1^–^ mutant strains of *P. pseudoalcaligenes* in ammonium.

TABLE S3 Bacterial strains and plasmids used in this work.

TABLE S4 Primers used in this work.
