# Supplementary figures and images for: Role of the Dihydrodipicolinate Synthase DapA1 on Iron Homeostasis During Cyanide Assimilation by the Alkaliphilic Bacterium Pseudomonas pseudoalcaligenes CECT5344
Source: Front Microbiol. 2020 Jan 23;11:28. doi: 10.3389/fmicb.2020.00028 (PMC6989483; doi:10.3389/fmicb.2020.00028)

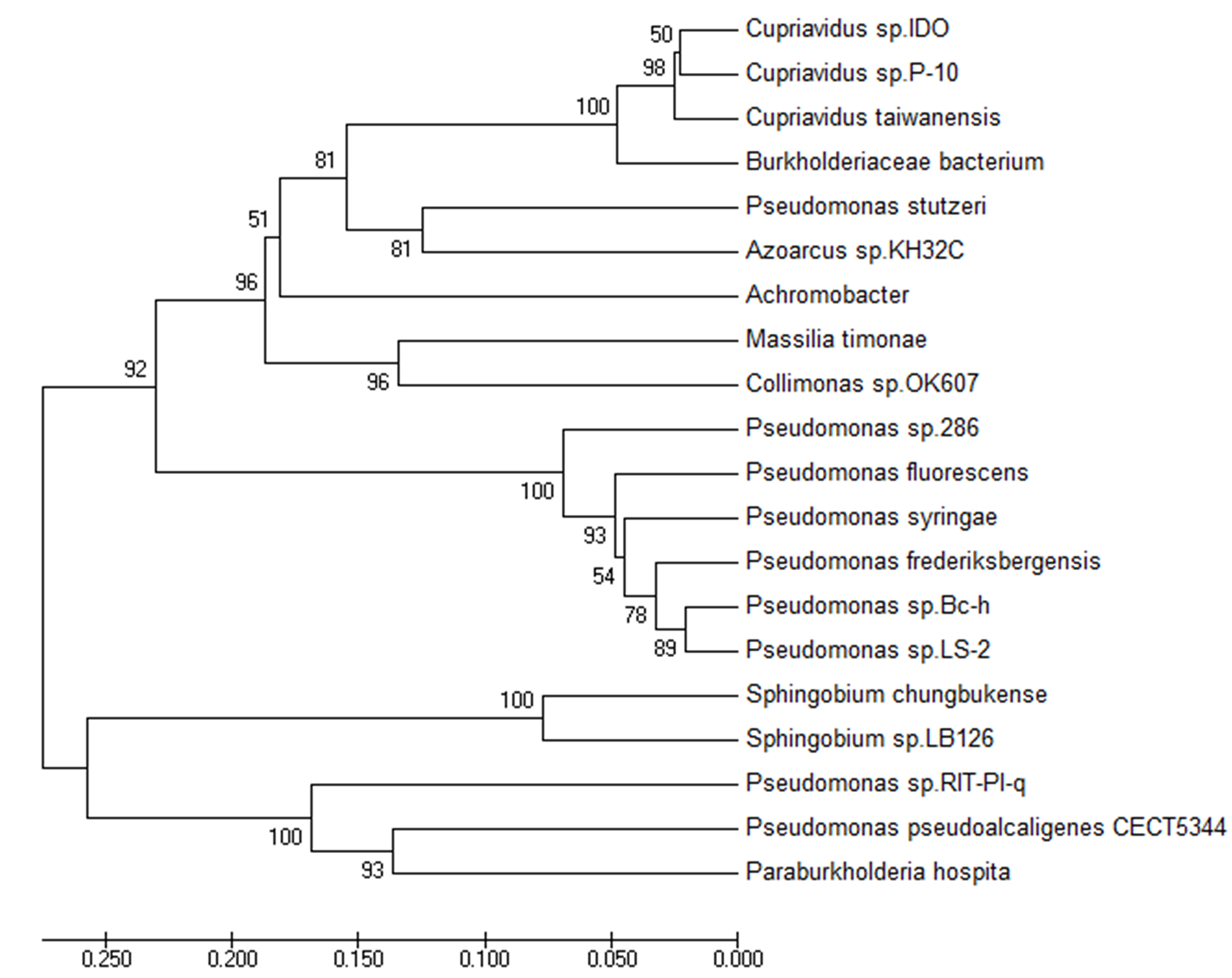

Supplement: FIGURE S1 — Evolutionary relationships of DapA taxa in bacterial strains containing in their genome genes coding for DapA, CioAB and NitC proteins MEGA7 was used to perform this analysis. The optimal tree resulted with a branch length of 2.51202227 is shown. The tree is drawn to scale, with branch lengths in the same units as those of the evolutionary distances used to infer the phylogenetic tree. Bootstrap values indicate the percentage in which a certain bipartition of the taxon set is present in 1000 bootstrap trees. [file Image_1.TIF]

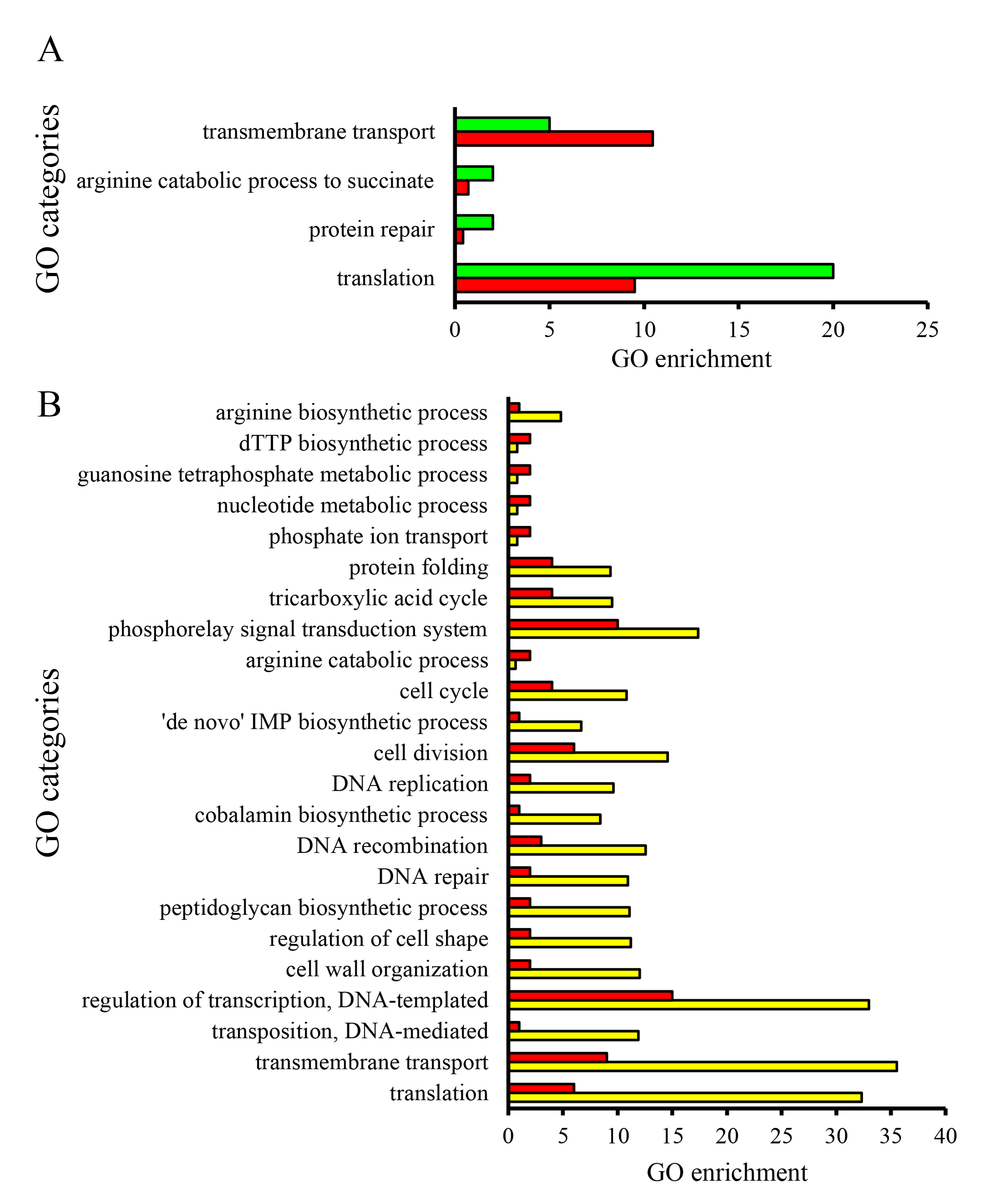

Supplement: FIGURE S2 — Gene ontology (GO) category analysis of proteins differentially affected in the wild-type strain and the dapA1– mutant of P. pseudoalcaligenes CECT5344. (A) Significative changes in GO groups among proteins over-represented in the wild-type strain of P. pseudoalcaligenes CECT5344 grown with the jewelry residue (green). The genome of the wild-type strain was considered as reference (red). (B) Significative changes in GO groups among proteins down-represented in the wild-type strain of P. pseudoalcaligenes CECT5344 cultured with the jewelry residue (yellow). The genome of the wild-type strain was considered as reference (red). [file Image_2.TIF]
