# Supplementary material for: Role of the Dihydrodipicolinate Synthase DapA1 on Iron Homeostasis During Cyanide Assimilation by the Alkaliphilic Bacterium Pseudomonas pseudoalcaligenes CECT5344
Source: Front Microbiol. 2020 Jan 23;11:28. doi: 10.3389/fmicb.2020.00028 (PMC6989483; doi:10.3389/fmicb.2020.00028)
Supplement: TABLE S1 — Quantitative proteomic analysis of the wild-type (reference) and DapA1– mutant strains of P. pseudoalcaligenes in jewelry residue. [file Table_1.DOCX]

**Table S1**. Quantitative proteomic analysis of the wild-type (reference) and DapA1ˉ mutant strains of *P. pseudoalcaligenes* in jewelry residue.

| **Protein ID^1^** | **Gene ID^3^** | **Gene ID^2^** | **Protein name** | **Location^4^** | **FC^5^** |
| --- | --- | --- | --- | --- | --- |
| W6R260 | PPSAL_1884 | BN5_1907 | 4-hydroxy-tetrahydrodipicolinate synthase (HTPA synthase) (EC 4.3.3.7) | Cytoplasmic | 39.41 |
| W6R2X6 | PPSAL_4449 | BN5_4513 | 50S ribosomal protein L34 | Cytoplasmic | 17.23 |
| W6RDY8 | PPSAL_1495 | BN5_1500 | Carbon storage regulator homolog | Unknown | 13.56 |
| W6RJI2 | PPSAL_3382 | BN5_3432 | 30S ribosomal protein S20 | Cytoplasmic | 10.46 |
| W6QW61 | PPSAL_2531 | BN5_2565 | Chromosome partition protein Smc | Cytoplasmic | 7.71 |
| W6RCF5 | PPSAL_0959 | BN5_0967 | Tetratricopeptide TPR_4 | Extracellular | 6.97 |
| W6RE40 | PPSAL_1551 | BN5_1562 | Uncharacterized protein | Cytoplasmic | 6.81 |
| W6R3S3 | PPSAL_2442 | BN5_2472 | YciI like Protein | Unknown | 6.48 |
| W6RDQ8 | PPSAL_1415 | BN5_1420 | Uncharacterized protein yggL | Cytoplasmic | 6.37 |
| W6QZA0 | PPSAL_2734 | BN5_2773 | Sensor protein PhoQ (EC 2.7.3.-) | Membrane | 5.88 |
| W6R0V6 | PPSAL_3727 | BN5_3784 | 30S ribosomal protein S21 | Cytoplasmic | 5.53 |
| W6R9C0 | PPSAL_4388 | BN5_4452 | Alkyl hydroperoxide reductase AhpD (EC 1.11.1.15) | Unknown | 5.48 |
| W6RGP7 | PPSAL_2480 | BN5_2510 | RND family efflux transporter MFP subunit | Membrane | 5.23 |
| W6R1Y8 | PPSAL_3649 | BN5_3701 | 30S ribosomal protein S12 | Cytoplasmic | 5.15 |
| W6RJ15 | PPSAL_3158 | BN5_3203 | Isochorismatase hydrolase (EC 3.3.2.1) | Cytoplasmic | 4.99 |
| W6QVW7 | PPSAL_2421 | BN5_2451 | Type cbb3 cytochrome oxidase biogenesis protein CcoI (EC 3.6.3.4) | Membrane | 4.87 |
| W6R218 | PPSAL_4145 | BN5_4208 | Deoxyuridine 5'-triphosphate nucleotidohydrolase (dUTPase) (EC 3.6.1.23) (dUTP pyrophosphatase) | Cytoplasmic | 4.49 |
| W6QS66 | PPSAL_0207 | BN5_0209 | Alginate regulatory protein AlgP | Unknown | 4.14 |
| W6QTP6 | PPSAL_0713 | BN5_0719 | Extracellular solute-binding protein | Periplasmic | 4.11 |
| W6RDZ3 | PPSAL_1500 | BN5_1509 | Arc | Cytoplasmic | 4.08 |
| W6RBM7 | PPSAL_0622 | BN5_0627 | Cytochrome d ubiquinol oxidase, subunit I (EC 1.10.3.-) | Membrane | 4.01 |
| W6R0R6 | PPSAL_1412 | BN5_1417 | Ribosome modulation factor (RMF) | Unknown | 3.92 |
| W6QU54 | PPSAL_0888 | BN5_0894 | Peptidyl-prolyl cis-trans isomerase (EC 5.2.1.8) | Cytoplasmic | 3.81 |
| W6QYY0 | PPSAL_3458 | BN5_3508 | Lipopolysaccharide export system protein LptA | Unknown | 3.61 |
| W6QYF0 | PPSAL_3248 | BN5_3295 | Nuclease SbcCD subunit C | Cytoplasmic | 3.59 |
| W6QUH3 | PPSAL_1968 | BN5_1991 | Arsenate reductase, putative (EC 1.20.4.1) | Cytoplasmic | 3.57 |
| W6R2U2 | PPSAL_2099 | BN5_2125 | Nitrate reductase (EC 1.7.99.4) | Cytoplasmic | 3.48 |
| W6QWB1 | PPSAL_1661 | BN5_1672 | 5-methylthioadenosine/S-adenosylhomocysteine deaminase (MTA/SAH deaminase) (EC 3.5.4.28) (EC 3.5.4.31) | Cytoplasmic | 3.32 |
| W6QZL4 | PPSAL_3641 | BN5_3693 | 50S ribosomal protein L2 | Cytoplasmic | 3.30 |
| W6REX8 | PPSAL_1827 | BN5_1848 | NADH dehydrogenase I chain F (EC 1.6.5.3) | Cytoplasmic | 3.25 |
| W6QQB9 | PPSAL_0533 | BN5_0538 | 50S ribosomal protein L9 | Cytoplasmic | 3.20 |
| W6QSL3 | PPSAL_1319 | BN5_1324 | Diguanylate phosphodiesterase | Membrane | 3.17 |
| W6QY01 | PPSAL_2748 | BN5_2787 | NAD(P)H dehydrogenase (quinone) (EC 1.6.5.2) (NAD(P)H:quinone oxidoreductase) (NQO) | Unknown | 3.16 |
| W6QV43 | PPSAL_1252 | BN5_1257 | Probable transcriptional regulatory protein BN5_1257 | Cytoplasmic | 3.13 |
| W6RFM9 | PPSAL_2097 | BN5_2123 | Assimilatory nitrite reductase (Subunit) (EC 1.7.1.4) | Cytoplasmic | 3.10 |
| W6QRW6 | PPSAL_1069 | BN5_1073 | 30S ribosomal protein S16 | Cytoplasmic | 2.99 |
| W6R172 | PPSAL_4197 | BN5_4261 | Sulfatase-modifying factor 1 (EC 1.8.99.-) | OuterMembrane | 2.94 |
| W6RIM9 | PPSAL_3045 | BN5_3088 | Probable transcriptional regulatory protein BN5_3088 | Cytoplasmic | 2.94 |
| W6QXM0 | PPSAL_3016 | BN5_3058 | FMN-dependent NADH-azoreductase (EC 1.7.-.-) (Azo-dye reductase) (FMN-dependent NADH-azo compound oxidoreductase) | Cytoplasmic | 2.91 |
| W6QQS7 | PPSAL_0655 | BN5_0661 | Dephospho-CoA kinase (EC 2.7.1.24) (Dephosphocoenzyme A kinase) | Cytoplasmic | 2.88 |
| W6QU57 | PPSAL_1413 | BN5_1418 | Ribosomal RNA large subunit methyltransferase K/L [Includes: 23S rRNA m2G2445 methyltransferase (EC 2.1.1.173) (rRNA (guanine-N(2)-)-methyltransferase RlmL); 23S rRNA m7G2069 methyltransferase (EC 2.1.1.264) (rRNA (guanine-N(7)-)-methyltransferase RlmK)] | Cytoplasmic | 2.84 |
| W6QXU6 | PPSAL_2688 | BN5_2724 | Putative beta-barrel assembly-enhancing protease (EC 3.4.-.-) | Unknown | 2.83 |
| W6QUV5 | PPSAL_2098 | BN5_2124 | Nitrite reductase (NAD(P)H) small subunit (EC 1.7.1.4) | Unknown | 2.77 |
| H9N5D8 | PPSAL_1625 | BN5_1637 | FAD dependent oxidoreductase (FAD-dependent oxidoreductase) | Cytoplasmic | 2.76 |
| W6QZF3 | PPSAL_3621 | BN5_3673 | 30S ribosomal protein S11 | Cytoplasmic | 2.74 |
| W6R0X9 | PPSAL_3757 | BN5_3815 | Uncharacterized protein | Cytoplasmic | 2.73 |
| W6R0L4 | PPSAL_3162 | BN5_3207 | Basic membrane protein A2 Immunodominant antigen P39 | Unknown | 2.69 |
| W6RAS2 | PPSAL_0332 | BN5_0335 | Cytochrome c | Unknown | 2.67 |
| W6R036 | PPSAL_1150 | BN5_1154 | Uncharacterized protein | Unknown | 2.66 |
| W6R674 | PPSAL_3269 | BN5_3316 | Uncharacterized protein | Unknown | 2.63 |
| W6RHD6 | PPSAL_2670 | BN5_2705 | Arsenical pump-driving ATPase (EC 3.6.3.16) | Cytoplasmic | 2.62 |
| W6QQ36 | PPSAL_0438 | BN5_0441 | ABC transporter/ATPase component protein | Membrane | 2.62 |
| W6QXC5 | PPSAL_0150 | BN5_0151 | Flavin monoamine oxidase-related protein (EC 1.4.3.4) | Unknown | 2.60 |
| W6R1J6 | PPSAL_3549 | BN5_3601 | Inorganic pyrophosphatase (EC 3.6.1.1) (Pyrophosphate phospho-hydrolase) (PPase) | Cytoplasmic | 2.59 |
| W6QP07 | PPSAL_0064 | BN5_0064 | N5-carboxyaminoimidazole ribonucleotide synthase (N5-CAIR synthase) (EC 6.3.4.18) (5-(carboxyamino)imidazole ribonucleotide synthetase) | Membrane | 2.57 |
| W6RM98 | PPSAL_4446 | BN5_4510 | Membrane protein insertase YidC (Foldase YidC) (Membrane integrase YidC) (Membrane protein YidC) | Membrane | 2.56 |
| W6QU88 | PPSAL_1448 | BN5_1453 | 50S ribosomal protein L32 | Cytoplasmic | 2.56 |
| W6QY86 | PPSAL_2843 | BN5_2882 | Cytosine deaminase (EC 3.5.4.1) | Cytoplasmic | 2.55 |
| W6RC44 | PPSAL_0824 | BN5_0830 | Basic membrane lipoprotein | Unknown | 2.54 |
| W6QS51 | PPSAL_0182 | BN5_0184 | HAD-superfamily hydrolase (EC 3.1.3.77) | Cytoplasmic | 2.53 |
| W6RI57 | PPSAL_2885 | BN5_2924 | FAD dependent oxidoreductase | Cytoplasmic | 2.53 |
| W6QY24 | PPSAL_0449 | BN5_0452 | Potassium efflux system protein | Membrane | 2.44 |
| W6R1G7 | PPSAL_4302 | BN5_4366 | Transcriptional regulatory protein zraR | Cytoplasmic | 2.42 |
| W6R622 | PPSAL_3190 | BN5_3235 | Periplasmic oligopeptide-binding protein | Periplasmic | 2.41 |
| W6QRT8 | PPSAL_0102 | BN5_0102 | Homoserine kinase (HK) (HSK) (EC 2.7.1.39) | Cytoplasmic | 2.41 |
| W6RLY4 | PPSAL_4301 | BN5_4365 | 31 kDa immunogenic protein | Unknown | 2.41 |
| W6RKF5 | PPSAL_3645 | BN5_3697 | 30S ribosomal protein S10 | Cytoplasmic | 2.41 |
| W6R1N7 | PPSAL_4387 | BN5_4451 | Putative HTH-type transcriptional regulator ykgD | Unknown | 2.40 |
| W6QRE7 | PPSAL_0465 | BN5_0468 | ADP-heptose-LPS heptosyltransferase II (EC 2.4.-.-) | Cytoplasmic | 2.40 |
| W6QPP2 | PPSAL_0298 | BN5_0301 | Beta-ketoacyl synthase (EC 2.3.1.41) | Cytoplasmic | 2.40 |
| W6QU31 | PPSAL_1808 | BN5_1829 | Exodeoxyribonuclease III (EC 3.1.11.2) | Cytoplasmic | 2.37 |
| W6QXJ6 | PPSAL_0254 | BN5_0257 | Agmatine deiminase (EC 3.5.3.12) (Agmatine iminohydrolase) | Cytoplasmic | 2.36 |
| W6QSF0 | PPSAL_0817 | BN5_0823 | Uncharacterized protein | Unknown | 2.35 |
| W6REZ4 | PPSAL_1847 | BN5_1868 | Translation initiation factor IF-1 | Cytoplasmic | 2.34 |
| W6QZK7 | PPSAL_1011 | BN5_1014 | Glutathione S-transferase like protein (EC 2.5.1.18) | Unknown | 2.34 |
| W6QQ64 | PPSAL_0001 | BN5_0001 | Chromosomal replication initiator protein DnaA | Cytoplasmic | 2.33 |
| W6R0Z7 | PPSAL_1487 | BN5_1492 | Arginine N-succinyltransferase (EC 2.3.1.109) | Cytoplasmic | 2.33 |
| W6QT34 | PPSAL_0531 | BN5_0536 | 30S ribosomal protein S18 | Cytoplasmic | 2.32 |
| W6R087 | PPSAL_3909 | BN5_3968 | UPF0001 protein | Cytoplasmic | 2.32 |
| W6QZG6 | PPSAL_3631 | BN5_3683 | 30S ribosomal protein S14 | Cytoplasmic | 2.32 |
| W6RCM8 | PPSAL_1038 | BN5_1042 | Lipoprotein nlpD/lppB homolog | Unknown | 2.31 |
| W6RJS0 | PPSAL_3462 | BN5_3512 | PTS IIA-like nitrogen-regulatory protein PtsN (EC 2.7.1.69) | Cytoplasmic | 2.31 |
| W6QUS5 | PPSAL_2068 | BN5_2093 | Cytochrome c family protein | Unknown | 2.30 |
| W6QW39 | PPSAL_2125 | BN5_2151 | 2-dehydropantoate 2-reductase (EC 1.1.1.169) (Ketopantoate reductase) | Cytoplasmic | 2.30 |
| H9N5E3 | PPSAL_1621 | BN5_1633 | Radical SAM domain-containing protein (Radical SAM domain-containing proteinBiotin synthase-related enzymeRibosomal RNA large subunit methyltransferase N) (EC 2.1.1.-) | Unknown | 2.29 |
| W6RC71 | PPSAL_0864 | BN5_0870 | Peptidase M48, Ste24p (EC 3.4.24.-) | Unknown | 2.29 |
| W6QR76 | PPSAL_0400 | BN5_0403 | Phosphoribosyl-ATP pyrophosphatase (PRA-PH) (EC 3.6.1.31) | Cytoplasmic | 2.28 |
| W6R0I3 | PPSAL_3618 | BN5_3670 | 50S ribosomal protein L17 | Cytoplasmic | 2.28 |
| W6RBC9 | PPSAL_0547 | BN5_0552 | Urease accessory protein UreG | Cytoplasmic | 2.26 |
| W6RFX0 | PPSAL_2151 | BN5_2178 | Succinate dehydrogenase, iron-sulfur protein (EC 1.3.99.1) | Membrane | 2.25 |
| W6QXI3 | PPSAL_2111 | BN5_2137 | Aconitate hydratase (EC 4.2.1.3) | Cytoplasmic | 2.22 |
| W6QZC4 | PPSAL_0911 | BN5_0917 | Transcription elongation factor GreA (Transcript cleavage factor GreA) | Cytoplasmic | 2.22 |
| W6R0J9 | PPSAL_3633 | BN5_3685 | 50S ribosomal protein L24 | Cytoplasmic | 2.22 |
| W6R1Z8 | PPSAL_3659 | BN5_3715 | Sporulation domain-containing protein | Unknown | 2.20 |
| W6R793 | PPSAL_3627 | BN5_3679 | 30S ribosomal protein S5 | Cytoplasmic | 2.18 |
| W6QV68 | PPSAL_2167 | BN5_2194 | Peptide methionine sulfoxide reductase MsrB (EC 1.8.4.12) (Peptide-methionine (R)-S-oxide reductase) | Cytoplasmic | 2.17 |
| W6QY14 | PPSAL_0439 | BN5_0442 | Cyanate hydratase (Cyanase) (EC 4.2.1.104) (Cyanate hydrolase) (Cyanate lyase) | Cytoplasmic | 2.17 |
| W6RB14 | PPSAL_0432 | BN5_0435 | Sporulation domain-containing protein | Unknown | 2.16 |
| W6QUK4 | PPSAL_1062 | BN5_1066 | Carnitine operon protein caiE | Cytoplasmic | 2.16 |
| W6QWW4 | PPSAL_0030 | BN5_0030 | Threonylcarbamoyl-AMP synthase (TC-AMP synthase) (EC 2.7.7.87) (L-threonylcarbamoyladenylate synthase) (t(6)A37 threonylcarbamoyladenosine biosynthesis protein TsaC) (tRNA threonylcarbamoyladenosine biosynthesis protein TsaC) | Cytoplasmic | 2.15 |
| W6RF25 | PPSAL_1887 | BN5_1910 | Cysteine synthase (EC 2.5.1.47) | Cytoplasmic | 2.15 |
| W6QXT4 | PPSAL_3080 | BN5_3124 | Cell division topological specificity factor | Cytoplasmic | 2.15 |
| W6R0J3 | PPSAL_3628 | BN5_3680 | 50S ribosomal protein L18 | Cytoplasmic | 2.15 |
| W6QVN8 | PPSAL_2335 | BN5_2366 | Lipoprotein, putative | Unknown | 2.13 |
| W6QTY1 | PPSAL_0808 | BN5_0814 | Hydrophobe/amphiphile efflux-1 (HAE1) family protein | Membrane | 2.13 |
| W6QRJ1 | PPSAL_0505 | BN5_0508 | Sulfurtransferase | Cytoplasmic | 2.12 |
| W6R2N9 | PPSAL_4369 | BN5_4433 | Peptide methionine sulfoxide reductase MsrB (EC 1.8.4.12) (Peptide-methionine (R)-S-oxide reductase) | Cytoplasmic | 2.12 |
| W6QYC2 | PPSAL_2878 | BN5_2917 | Outer membrane porin (EC 3.4.21.-) | OuterMembrane | 2.11 |
| W6RAK9 | PPSAL_0257 | BN5_0260 | Putrescine-binding periplasmic protein | Periplasmic | 2.11 |
| W6QSS9 | PPSAL_0431 | BN5_0434 | 3-dehydroquinate synthase (EC 4.2.3.4) | Cytoplasmic | 2.10 |
| W6RAY6 | PPSAL_0407 | BN5_0410 | Poly(Hydroxyalkanoate) granule-associated protein | Cytoplasmic | 2.09 |
| W6RIM2 | PPSAL_3035 | BN5_3078 | TPR repeat-containing protein | Unknown | 2.09 |
| W6RDU5 | PPSAL_1455 | BN5_1460 | Uncharacterized protein | Unknown | 2.07 |
| W6RB46 | PPSAL_0467 | BN5_0470 | UDP-glucose:(Heptosyl)LPS alpha-1,3-glucosyltransferase (EC 2.4.1.-) | Cytoplasmic | 2.07 |
| W6QTQ8 | PPSAL_0728 | BN5_0734 | RNAse G (EC 3.1.4.-) | Cytoplasmic | 2.04 |
| W6QWX7 | PPSAL_2766 | BN5_2805 | 5'-nucleotidase SurE (EC 3.1.3.5) (Nucleoside 5'-monophosphate phosphohydrolase) | Unknown | 2.04 |
| W6RDT9 | PPSAL_1450 | BN5_1455 | Malonyl CoA-acyl carrier protein transacylase (EC 2.3.1.39) | Cytoplasmic | 2.04 |
| W6R0F5 | PPSAL_3593 | BN5_3645 | Riboflavin synthase, alpha subunit (EC 2.5.1.9) | Cytoplasmic | 2.03 |
| W6QQ80 | PPSAL_0026 | BN5_0026 | Methionyl-tRNA formyltransferase (EC 2.1.2.9) | Cytoplasmic | 2.03 |
| W6R7D8 | PPSAL_3671 | BN5_3728 | Anhydro-N-acetylmuramic acid kinase (EC 2.7.1.170) (AnhMurNAc kinase) | Cytoplasmic | 2.03 |
| W6QVK9 | PPSAL_1419 | BN5_1424 | Deoxyguanosinetriphosphate triphosphohydrolase (EC 3.1.5.1) | Cytoplasmic | 2.02 |
| W6QZJ6 | PPSAL_3636 | BN5_3688 | 50S ribosomal protein L29 | Cytoplasmic | 2.02 |
| W6R8N2 | PPSAL_4139 | BN5_4202 | 50S ribosomal protein L33 | Cytoplasmic | 2.01 |
| W6QVT5 | PPSAL_1489 | BN5_1494 | N-succinylarginine dihydrolase (EC 3.5.3.23) | Cytoplasmic | 2.01 |
| W6QYL1 | PPSAL_3323 | BN5_3372 | 50S ribosomal protein L25 (General stress protein CTC) | Cytoplasmic | -2.01 |
| W6QZI6 | PPSAL_2819 | BN5_2858 | 3-oxoacyl-(Acyl-carrier-protein) synthase III, putative (EC 2.3.1.41) | Cytoplasmic | -2.01 |
| W6QSH0 | PPSAL_0837 | BN5_0843 | Methylmalonate-semialdehyde dehydrogenase (EC 1.2.1.27) | Cytoplasmic | -2.01 |
| W6QYN0 | PPSAL_3348 | BN5_3398 | UPF0246 protein BN5_3398 | Cytoplasmic | -2.01 |
| W6RA33 | PPSAL_0108 | BN5_0108 | Thiol:disulfide interchange protein | Periplasmic | -2.03 |
| W6R1N1 | PPSAL_1674 | BN5_1685 | UDP-N-acetylglucosamine 2-epimerase (EC 5.1.3.14) | Cytoplasmic | -2.03 |
| W6QZD9 | PPSAL_0926 | BN5_0934 | Polyribonucleotide nucleotidyltransferase (EC 2.7.7.8) (Polynucleotide phosphorylase) (PNPase) | Cytoplasmic | -2.04 |
| W6R2K7 | PPSAL_3872 | BN5_3931 | Na+/solute symporter | Membrane | -2.04 |
| W6R420 | PPSAL_4320 | BN5_4384 | Putative oxidoreductase (EC 1.1.1.-) | Cytoplasmic | -2.05 |
| W6QW92 | PPSAL_2576 | BN5_2610 | Cytochrome c-type biogenesis protein cycH | Membrane | -2.05 |
| W6RCN8 | PPSAL_1048 | BN5_1052 | GMP synthase [glutamine-hydrolyzing] (EC 6.3.5.2) (GMP synthetase) (Glutamine amidotransferase) | Cytoplasmic | -2.05 |
| W6RDI9 | PPSAL_1323 | BN5_1328 | Multidrug resistance protein mdtF | Membrane | -2.05 |
| W6QUB2 | PPSAL_1898 | BN5_1921 | Serine hydroxymethyltransferase (SHMT) (Serine methylase) (EC 2.1.2.1) | Cytoplasmic | -2.06 |
| W6RHN5 | PPSAL_2740 | BN5_2779 | Nitrate reductase (EC 1.7.99.4) | Periplasmic | -2.08 |
| W6QXL2 | PPSAL_3006 | BN5_3048 | 6-phosphogluconate dehydratase (EC 4.2.1.12) | Cytoplasmic | -2.08 |
| W6RL26 | PPSAL_3978 | BN5_4037 | Putative membrane transport ATPase (EC 3.6.3.3) | Membrane | -2.08 |
| W6QZJ0 | PPSAL_2824 | BN5_2863 | Histidine triad (HIT) protein (EC 3.6.1.17) | Cytoplasmic | -2.09 |
| W6QZ75 | PPSAL_3556 | BN5_3608 | 1,6-dihydroxycyclohexa-2, 4-diene-1-carboxylatedehydrogenase (EC 1.3.1.25) | Cytoplasmic | -2.09 |
| W6R1W7 | PPSAL_1784 | BN5_1805 | Uncharacterized protein | Unknown | -2.09 |
| W6R042 | PPSAL_3864 | BN5_3923 | Amino acid-binding ACT domain-containing protein (EC 2.7.7.59) | Cytoplasmic | -2.10 |
| W6QWV3 | PPSAL_2741 | BN5_2780 | Periplasmic nitrate reductase, electron transfer subunit (Diheme cytochrome c NapB) | Periplasmic | -2.10 |
| W6QQ73 | PPSAL_0016 | BN5_0016 | Glycine--tRNA ligase alpha subunit (EC 6.1.1.14) (Glycyl-tRNA synthetase alpha subunit) (GlyRS) | Cytoplasmic | -2.11 |
| W6QSD2 | PPSAL_0276 | BN5_0279 | Osmolarity response regulator | Cytoplasmic | -2.11 |
| W6QZM8 | PPSAL_1031 | BN5_1034 | Zinc-containing alcohol dehydrogenase superfamily (EC 1.1.1.1) | Membrane | -2.12 |
| W6QZ06 | PPSAL_2644 | BN5_2678 | Chemotaxis protein methyltransferase (EC 2.1.1.80) | Cytoplasmic | -2.12 |
| W6RIM5 | PPSAL_3040 | BN5_3083 | MotA/TolQ/ExbB proton channel | Membrane | -2.16 |
| W6R1I1 | PPSAL_3986 | BN5_4045 | D-3-phosphoglycerate dehydrogenase (EC 1.1.1.95) | Cytoplasmic | -2.17 |
| W6REQ8 | PPSAL_1747 | BN5_1768 | Uncharacterized protein | Periplasmic | -2.17 |
| W6QY06 | PPSAL_2753 | BN5_2792 | Phosphoribosylformylglycinamidine cyclo-ligase (EC 6.3.3.1) (AIR synthase) (AIRS) (Phosphoribosyl-aminoimidazole synthetase) | Cytoplasmic | -2.18 |
| W6QTR5 | PPSAL_1683 | BN5_1694 | 4-hydroxy-2-oxovalerate aldolase (EC 4.1.3.39) | Unknown | -2.19 |
| W6QSK8 | PPSAL_0872 | BN5_0878 | Type V secretory pathway, adhesin AidA | OuterMembrane | -2.20 |
| W6RFZ1 | PPSAL_2175 | BN5_2203 | Putative protease SohB (EC 3.4.21.-) | Membrane | -2.20 |
| W6QQJ3 | PPSAL_0608 | BN5_0613 | Biosynthetic arginine decarboxylase (ADC) (EC 4.1.1.19) | Unknown | -2.21 |
| W6QXP8 | PPSAL_3046 | BN5_3089 | Aspartyl-tRNA synthetase (EC 6.1.1.12) | Cytoplasmic | -2.21 |
| W6QT70 | PPSAL_1076 | BN5_1080 | Homoserine dehydrogenase (EC 1.1.1.3) | Cytoplasmic | -2.21 |
| W6R0L7 | PPSAL_1365 | BN5_1370 | Pirin-like protein CC_1473 | Cytoplasmic | -2.22 |
| W6QP53 | PPSAL_0119 | BN5_0119 | Phosphate-specific transport system accessory protein PhoU | Cytoplasmic | -2.22 |
| W6QZK4 | PPSAL_3285 | BN5_3332 | Ornithine carbamoyltransferase (OTCase) (EC 2.1.3.3) | Cytoplasmic | -2.24 |
| W6QZG2 | PPSAL_2794 | BN5_2833 | Elongation factor Ts (EF-Ts) | Cytoplasmic | -2.25 |
| W6R7V2 | PPSAL_3880 | BN5_3939 | CBS domain protein | Cytoplasmic | -2.25 |
| W6R850 | PPSAL_3985 | BN5_4044 | D-2-hydroxyglutarate dehydrogenase, mitochondrial (EC 1.1.99.-) | Cytoplasmic | -2.25 |
| W6R1M2 | PPSAL_1664 | BN5_1675 | DNA gyrase subunit A (EC 5.99.1.3) | Cytoplasmic | -2.26 |
| W6QXP3 | PPSAL_2155 | BN5_2182 | Citrate synthase | Cytoplasmic | -2.26 |
| W6QXK3 | PPSAL_2996 | BN5_3036 | Alkyl hydroperoxide reductase subunit C (EC 1.11.1.15) | Cytoplasmic | -2.26 |
| W6QXP0 | PPSAL_0314 | BN5_0317 | Amino-acid carrier protein | Membrane | -2.26 |
| W6RE45 | PPSAL_1556 | BN5_1567 | Bacterial luciferase family protein (EC 1.14.-.-) | Cytoplasmic | -2.28 |
| W6RLL1 | PPSAL_4147 | BN5_4210 | Acetylglutamate kinase (EC 2.7.2.8) (N-acetyl-L-glutamate 5-phosphotransferase) (NAG kinase) (AGK) | Cytoplasmic | -2.28 |
| W6R0A0 | PPSAL_1225 | BN5_1229 | ABC transporter, binding protein | Unknown | -2.28 |
| W6QY82 | PPSAL_0504 | BN5_0507 | Phosphatidylserine decarboxylase proenzyme (EC 4.1.1.65) [Cleaved into: Phosphatidylserine decarboxylase beta chain; Phosphatidylserine decarboxylase alpha chain] | Membrane | -2.30 |
| W6QXE9 | PPSAL_2071 | BN5_2096 | Uncharacterized protein | Unknown | -2.30 |
| W6R0Q9 | PPSAL_3226 | BN5_3272 | Inositol-1-monophosphatase (EC 3.1.3.25) | Cytoplasmic | -2.30 |
| W6QXM7 | PPSAL_0294 | BN5_0297 | LysM domain/BON superfamily protein | Unknown | -2.31 |
| W6QQR4 | PPSAL_0638 | BN5_0643 | Cyclic diguanosine monophosphate-binding protein (c-di-GMP-binding protein) (Pilz domain-containing protein) | Cytoplasmic | -2.32 |
| W6QXH7 | PPSAL_2593 | BN5_2627 | Chemotaxis response regulator protein-glutamate methylesterase (EC 3.1.1.61) | Cytoplasmic | -2.33 |
| W6R1X9 | PPSAL_1799 | BN5_1820 | Methyl-accepting chemotaxis protein mcpB | Membrane | -2.33 |
| W6R0Q1 | PPSAL_1395 | BN5_1400 | FAD:protein FMN transferase (EC 2.7.1.180) | Cytoplasmic | -2.34 |
| W6QP19 | PPSAL_0079 | BN5_0079 | Aspartate ammonia-lyase (EC 4.3.1.1) | Cytoplasmic | -2.34 |
| W6RIB2 | PPSAL_2940 | BN5_2979 | Argininosuccinate synthase (EC 6.3.4.5) (Citrulline--aspartate ligase) | Cytoplasmic | -2.35 |
| W6R0K4 | PPSAL_1348 | BN5_1355 | Uncharacterized protein | Cytoplasmic | -2.35 |
| W6QYJ4 | PPSAL_2948 | BN5_2987 | Branched-chain amino acid transport system substrate-binding protein | Periplasmic | -2.35 |
| W6R3T0 | PPSAL_4235 | BN5_4299 | Oligopeptidase A (EC 3.4.24.70) | Cytoplasmic | -2.35 |
| W6QSU2 | PPSAL_1394 | BN5_1399 | Na(+)-translocating NADH-quinone reductase subunit F (Na(+)-NQR subunit F) (Na(+)-translocating NQR subunit F) (EC 1.6.5.8) (NQR complex subunit F) (NQR-1 subunit F) | Unknown | -2.36 |
| W6R339 | PPSAL_2168 | BN5_2195 | Putative aminotransferase (EC 2.6.1.1) | Cytoplasmic | -2.37 |
| W6QQ99 | PPSAL_0056 | BN5_0056 | TRAP dicarboxylate transporter-DctP subunit | Unknown | -2.39 |
| W6QVV2 | PPSAL_2020 | BN5_2045 | Methyl-accepting chemotaxis sensory transducer | Membrane | -2.39 |
| W6QSD9 | PPSAL_0286 | BN5_0289 | Uncharacterized protein | Unknown | -2.40 |
| W6R5F7 | PPSAL_3012 | BN5_3054 | DEAD/DEAH box helicase domain-containing protein | Cytoplasmic | -2.40 |
| W6QXR3 | PPSAL_2179 | BN5_2207 | Oxidoreductase, short-chain dehydrogenase/reductase family (EC 1.1.1.-) | Cytoplasmic | -2.41 |
| W6RAM8 | PPSAL_0282 | BN5_0285 | Phosphoesterase (EC 3.1.3.4) | Membrane | -2.41 |
| W6RH00 | PPSAL_2595 | BN5_2629 | Protein phosphatase CheZ (EC 3.1.3.-) (Chemotaxis protein CheZ) | Cytoplasmic | -2.42 |
| W6QXS8 | PPSAL_2194 | BN5_2222 | Peptide ABC transporter, periplasmic peptide-binding protein | Periplasmic | -2.43 |
| W6QS37 | PPSAL_0702 | BN5_0708 | Uncharacterized protein | Unknown | -2.45 |
| W6QU89 | PPSAL_0918 | BN5_0924 | Triosephosphate isomerase (TIM) (TPI) (EC 5.3.1.1) (Triose-phosphate isomerase) | Cytoplasmic | -2.46 |
| W6QUD3 | PPSAL_1488 | BN5_1493 | N-succinylglutamate 5-semialdehyde dehydrogenase (EC 1.2.1.71) (Succinylglutamic semialdehyde dehydrogenase) (SGSD) | Cytoplasmic | -2.47 |
| W6QSI1 | PPSAL_0331 | BN5_0334 | Methyl-accepting chemotaxis protein I | Membrane | -2.47 |
| W6QWE0 | PPSAL_1696 | BN5_1708 | Aminotransferase (EC 2.6.1.-) | Cytoplasmic | -2.48 |
| W6R3L0 | PPSAL_2382 | BN5_2412 | Glutathione S-transferase family protein (EC 2.5.1.18) | Cytoplasmic | -2.49 |
| W6QS62 | PPSAL_0202 | BN5_0204 | Uroporphyrinogen-III synthetase (EC 4.2.1.75) | Membrane | -2.49 |
| W6QY39 | PPSAL_2318 | BN5_2349 | Pentapeptide repeat-containing protein | Extracellular | -2.51 |
| W6QVA8 | PPSAL_2206 | BN5_2234 | Amidotransferase | Cytoplasmic | -2.52 |
| W6QVU2 | PPSAL_1494 | BN5_1499 | Aspartokinase (EC 2.7.2.4) | Cytoplasmic | -2.52 |
| W6QVZ2 | PPSAL_2065 | BN5_2090 | Copper-resistance protein CopA | Periplasmic | -2.54 |
| W6QSI2 | PPSAL_0847 | BN5_0853 | 3-ketoacyl-(Acyl-carrier-protein) reductase (EC 1.1.1.100) | Cytoplasmic | -2.54 |
| W6R0N7 | PPSAL_3643 | BN5_3695 | 50S ribosomal protein L4 | Cytoplasmic | -2.55 |
| W6R3Q1 | PPSAL_2422 | BN5_2452 | Cytochrome oxidase maturation protein, cbb3-type, CcoS | Unknown | -2.55 |
| W6R374 | PPSAL_2217 | BN5_2245 | Uncharacterized protein | Cytoplasmic | -2.56 |
| W6RLE3 | PPSAL_4102 | BN5_4165 | Antioxidant, AhpC/Tsa family (EC 1.11.1.15) | Cytoplasmic | -2.60 |
| W6QU17 | PPSAL_0848 | BN5_0854 | Polyprenyl synthetase (EC 2.5.1.-) | Cytoplasmic | -2.60 |
| W6QTV8 | PPSAL_1728 | BN5_1749 | ATP-dependent Clp protease proteolytic subunit (EC 3.4.21.92) (Endopeptidase Clp) | Cytoplasmic | -2.62 |
| W6QVC9 | PPSAL_2226 | BN5_2254 | Transaldolase (EC 2.2.1.2) | Unknown | -2.63 |
| W6QUT3 | PPSAL_1634 | BN5_1646 | Small heat shock protein | Cytoplasmic | -2.63 |
| W6QXI1 | PPSAL_2598 | BN5_2632 | Site-determining protein | Membrane | -2.67 |
| W6QY40 | PPSAL_2793 | BN5_2832 | Uridylate kinase (UK) (EC 2.7.4.22) (Uridine monophosphate kinase) (UMP kinase) (UMPK) | Cytoplasmic | -2.69 |
| W6QNV1 | PPSAL_0004 | BN5_0004 | DNA gyrase subunit B (EC 5.99.1.3) | Cytoplasmic | -2.71 |
| W6RDI4 | PPSAL_1318 | BN5_1323 | Acetyl-coenzyme A synthetase (AcCoA synthetase) (Acs) (EC 6.2.1.1) (Acetate--CoA ligase) (Acyl-activating enzyme) | Cytoplasmic | -2.76 |
| W6QYC1 | PPSAL_2414 | BN5_2444 | Cbb3-type cytochrome c oxidase subunit | Unknown | -2.81 |
| W6QZ51 | PPSAL_0836 | BN5_0842 | Beta-alanine-pyruvate transaminase (EC 2.6.1.18) | Cytoplasmic | -2.83 |
| W6QUC8 | PPSAL_0963 | BN5_0971 | Acetolactate synthase 3 regulatory subunit (EC 2.2.1.6) | Cytoplasmic | -2.84 |
| W6R2A2 | PPSAL_4229 | BN5_4293 | Uncharacterized protein | Unknown | -2.85 |
| W6R3K8 | PPSAL_4156 | BN5_4219 | Guanosine-3',5'-bis(Diphosphate) 3'-pyrophosphohydrolase (EC 3.1.7.2) | Cytoplasmic | -2.87 |
| W6R1E1 | PPSAL_3941 | BN5_4000 | Phosphopantetheine adenylyltransferase (EC 2.7.7.3) (Dephospho-CoA pyrophosphorylase) (Pantetheine-phosphate adenylyltransferase) (PPAT) | Cytoplasmic | -2.91 |
| W6R8X4 | PPSAL_4228 | BN5_4292 | Uncharacterized protein | Cytoplasmic | -2.91 |
| W6QRL9 | PPSAL_0530 | BN5_0535 | 30S ribosomal protein S6 | Cytoplasmic | -3.07 |
| W6QY80 | PPSAL_2838 | BN5_2877 | Acyl-CoA synthetase (EC 6.2.1.3) | Cytoplasmic | -3.09 |
| W6QSB7 | PPSAL_1229 | BN5_1233 | Uncharacterized protein | Cytoplasmic | -3.09 |
| W6QW84 | PPSAL_2149 | BN5_2176 | Dihydrolipoyllysine-residue succinyltransferase component of 2-oxoglutarate dehydrogenase complex (EC 2.3.1.61) (2-oxoglutarate dehydrogenase complex component E2) | Cytoplasmic | -3.10 |
| W6QUQ3 | PPSAL_1117 | BN5_1121 | Glutaredoxin | Unknown | -3.15 |
| W6R1S4 | PPSAL_3624 | BN5_3676 | Protein translocase subunit SecY | Membrane | -3.21 |
| W6R4K8 | PPSAL_2682 | BN5_2718 | 4-hydroxy-tetrahydrodipicolinate synthase (HTPA synthase) (EC 4.3.3.7) | Cytoplasmic | -3.25 |
| W6QTC7 | PPSAL_1126 | BN5_1130 | Osmoregulated proline transporter Sodium/proline symporter | Membrane | -3.28 |
| W6REZ7 | PPSAL_1852 | BN5_1873 | Outer-membrane lipoprotein carrier protein | Periplasmic | -3.29 |
| W6R782 | PPSAL_3617 | BN5_3669 | Catalase-peroxidase (CP) (EC 1.11.1.21) (Peroxidase/catalase) | Cytoplasmic | -3.31 |
| W6R020 | PPSAL_3004 | BN5_3044 | Peptidyl-prolyl cis-trans isomerase (EC 5.2.1.8) | Cytoplasmic | -3.32 |
| W6QZ12 | PPSAL_0781 | BN5_0787 | AsnC family transcriptional regulator | Cytoplasmic | -3.32 |
| W6RAC3 | PPSAL_0148 | BN5_0149 | Putative endoribonuclease L-PSP | Unknown | -3.33 |
| W6R013 | PPSAL_1135 | BN5_1139 | Superoxide dismutase (EC 1.15.1.1) | Periplasmic | -3.34 |
| W6R432 | PPSAL_2537 | BN5_2571 | Fatty acid metabolism regulator protein | Cytoplasmic | -3.52 |
| W6QZT0 | PPSAL_2914 | BN5_2953 | Outer membrane porin F | OuterMembrane | -3.54 |
| W6RET1 | PPSAL_1772 | BN5_1793 | 3-hydroxydecanoyl-[acyl-carrier-protein] dehydratase (EC 4.2.1.59) (3-hydroxyacyl-[acyl-carrier-protein] dehydratase FabA) (Beta-hydroxydecanoyl thioester dehydrase) (Trans-2-decenoyl-[acyl-carrier-protein] isomerase) (EC 5.3.3.14) | Cytoplasmic | -3.55 |
| W6R799 | PPSAL_3632 | BN5_3684 | 50S ribosomal protein L5 | Cytoplasmic | -3.57 |
| W6R0R8 | PPSAL_4083 | BN5_4146 | Uncharacterized protein | Unknown | -3.58 |
| W6R8V0 | PPSAL_4203 | BN5_4267 | Putative virulence effector protein | Unknown | -3.64 |
| W6QWH2 | PPSAL_1731 | BN5_1752 | DNA-binding protein HU | Cytoplasmic | -3.66 |
| W6QYX1 | PPSAL_3448 | BN5_3498 | UDP-N-acetylglucosamine 1-carboxyvinyltransferase (EC 2.5.1.7) (Enoylpyruvate transferase) (UDP-N-acetylglucosamine enolpyruvyl transferase) (EPT) | Cytoplasmic | -3.70 |
| W6QZB3 | PPSAL_0901 | BN5_0907 | Ferric uptake regulation protein | Cytoplasmic | -3.71 |
| W6R0J6 | PPSAL_4018 | BN5_4079 | Membrane-bound lytic murein transglycosylase F | Periplasmic | -3.78 |
| W6R0Q0 | PPSAL_4068 | BN5_4131 | Putative capsule polysaccharide export protein | Unknown | -3.85 |
| W6QQK7 | PPSAL_0166 | BN5_0168 | Cytochrome c5-like protein | Unknown | -3.89 |
| W6R3J7 | PPSAL_4146 | BN5_4209 | Phosphomannomutase (EC 5.4.2.8) | Unknown | -3.90 |
| W6QVX1 | PPSAL_2426 | BN5_2456 | CRP/FNR family transcriptional regulator | Cytoplasmic | -3.93 |
| W6R2R0 | PPSAL_2069 | BN5_2094 | Uncharacterized protein | Unknown | -3.95 |
| W6QYI9 | PPSAL_2484 | BN5_2514 | Acyl-CoA dehydrogenase (EC 1.3.99.-) | Membrane | -4.08 |
| W6QPR3 | PPSAL_0318 | BN5_0321 | UPF0312 protein BN5_0321 | Unknown | -4.12 |
| W6RC60 | PPSAL_0849 | BN5_0855 | 50S ribosomal protein L21 | Cytoplasmic | -4.47 |
| W6QZN2 | PPSAL_1035 | BN5_1039 | 2-isopropylmalate synthase (EC 2.3.3.13) (Alpha-IPM synthase) (Alpha-isopropylmalate synthase) | Cytoplasmic | -4.49 |
| W6QU60 | PPSAL_1843 | BN5_1864 | Isocitrate dehydrogenase [NADP] (EC 1.1.1.42) | Cytoplasmic | -4.52 |
| W6QYD8 | PPSAL_2893 | BN5_2932 | Outer membrane lipoprotein SlyB | OuterMembrane | -4.54 |
| W6QQL3 | PPSAL_0176 | BN5_0178 | Nitrogen regulatory protein P-II | Membrane | -4.55 |
| W6QUJ2 | PPSAL_1549 | BN5_1560 | GTP pyrophosphokinase (EC 2.7.6.5) | Cytoplasmic | -4.72 |
| W6QXH2 | PPSAL_0219 | BN5_0222 | Delta-aminolevulinic acid dehydratase (EC 4.2.1.24) | Cytoplasmic | -4.75 |
| W6RM88 | PPSAL_4436 | BN5_4500 | ATP synthase subunit delta (ATP synthase F(1) sector subunit delta) (F-type ATPase subunit delta) (F-ATPase subunit delta) | Unknown | -4.78 |
| W6R1X7 | PPSAL_4135 | BN5_4198 | Laminin subunit gamma-1 | Unknown | -4.81 |
| W6R254 | PPSAL_1879 | BN5_1902 | Terminal oxidase subunit I (EC 1.10.3.-) | Membrane | -4.88 |
| W6QZF9 | PPSAL_3230 | BN5_3276 | Preprotein translocase | Membrane | -5.07 |
| W6R3F4 | PPSAL_2316 | BN5_2347 | Cytochrome c550 | Unknown | -5.08 |
| W6R3S5 | PPSAL_4230 | BN5_4294 | Uncharacterized protein | Unknown | -5.09 |
| W6QXI2 | PPSAL_0234 | BN5_0237 | Membrane-fusion protein | Membrane | -5.16 |
| W6RLB1 | PPSAL_4067 | BN5_4130 | Putative tyrosine-protein kinase epsB (EC 2.7.10.-) | Membrane | -5.28 |
| W6R176 | PPSAL_4202 | BN5_4266 | Putative virulence factor | OuterMembrane | -5.40 |
| W6QZB6 | PPSAL_3591 | BN5_3643 | 6,7-dimethyl-8-ribityllumazine synthase (DMRL synthase) (LS) (Lumazine synthase) (EC 2.5.1.78) | Cytoplasmic | -5.57 |
| W6QQA6 | PPSAL_0518 | BN5_0521 | RNA-binding protein Hfq | Cytoplasmic | -5.61 |
| W6R8K7 | PPSAL_4134 | BN5_4197 | Putative glutamine synthetase (EC 6.3.1.2) | Cytoplasmic | -5.91 |
| W6R686 | PPSAL_3284 | BN5_3331 | Arginine deiminase (ADI) (EC 3.5.3.6) (Arginine dihydrolase) (AD) | Cytoplasmic | -6.26 |
| W6QSQ4 | PPSAL_0406 | BN5_0409 | Poly(Hydroxyalkanoate) granule-associated protein (Phasin) | Cytoplasmic | -6.54 |
| W6QUH0 | PPSAL_1528 | BN5_1537 | Laminin subunit alphmethyl-accepting chemotaxis sensory transducer | Membrane | -7.33 |
| W6QZZ6 | PPSAL_3430 | BN5_3480 | Cytochrome b | Membrane | -7.42 |
| W6R9H1 | PPSAL_4438 | BN5_4502 | ATP synthase subunit c | Membrane | -7.45 |
| W6QYU3 | PPSAL_3048 | BN5_3091 | Ferritin, Dps family protein | Cytoplasmic | -7.84 |
| W6RCV7 | PPSAL_1108 | BN5_1112 | Bacterioferritin (EC 1.16.3.1) | Cytoplasmic | -7.97 |
| W6QYL6 | PPSAL_3328 | BN5_3377 | Uncharacterized protein | Unknown | -8.25 |
| W6R0E8 | PPSAL_1285 | BN5_1290 | Ribonucleoside-diphosphate reductase subunit beta (EC 1.17.4.1) | Cytoplasmic | -8.61 |
| W6QTC3 | PPSAL_0611 | BN5_0616 | Alpha-2-macroglobulin domain-containing protein | Unknown | -12.89 |
| W6R0W1 | PPSAL_1452 | BN5_1457 | Acyl carrier protein (ACP) | Cytoplasmic | -49.99 |
| W6R0A3 | PPSAL_3538 | BN5_3590 | Fatty-acyl-CoA synthase (EC 2.3.1.86) | Cytoplasmic | -68.48 |
| W6RK11 | PPSAL_3540 | BN5_3592 | Acyl-CoA dehydrogenase family protein | Cytoplasmic | -145.71 |
| W6R0I9 | PPSAL_3623 | BN5_3675 | 50S ribosomal protein L36 | Cytoplasmic | WT |
| W6R775 | PPSAL_3612 | BN5_3664 | dTDP-4-dehydrorhamnose reductase (EC 1.1.1.133) | Cytoplasmic | WT |
| W6R8T6 | PPSAL_4183 | BN5_4247 | Formyl-coenzyme A transferase (EC 2.8.3.16) | Cytoplasmic | WT |
| W6QYN9 | PPSAL_3363 | BN5_3413 | Uncharacterized protein | Membrane | WT |
| W6R445 | PPSAL_2547 | BN5_2581 | Peptidylprolyl isomerase (EC 5.2.1.8) | Cytoplasmic | WT |
| W6QWC1 | PPSAL_2188 | BN5_2216 | Ribonuclease H (RNase H) (EC 3.1.26.4) | Unknown | WT |
| W6R1J2 | PPSAL_3544 | BN5_3596 | Flavin prenyltransferase UbiX (EC 2.5.1.129) | Cytoplasmic | WT |
| W6R1D6 | PPSAL_3936 | BN5_3995 | Abhydrolase domain-containing protein 3 | Unknown | WT |
| W6QZH3 | PPSAL_2804 | BN5_2843 | Amino acid ABC transporter periplasmic protein | Unknown | WT |
| W6R2L1 | PPSAL_4344 | BN5_4408 | Uncharacterized protein | Cytoplasmic | WT |
| W6QQU2 | PPSAL_0270 | BN5_0273 | Acetylornithine deacetylase (EC 3.5.1.-) (EC 3.5.1.16) | Unknown | WT |
| W6QVJ2 | PPSAL_2290 | BN5_2319 | Uncharacterized protein | Unknown | WT |
| W6R2C6 | PPSAL_4259 | BN5_4323 | Lipoprotein, putative | Unknown | WT |
| W6QXE4 | PPSAL_2066 | BN5_2091 | Uncharacterized protein | Unknown | WT |
| W6QWM4 | PPSAL_2297 | BN5_2327 | 50S ribosomal protein L35 | Cytoplasmic | WT |
| W6QT19 | PPSAL_0516 | BN5_0519 | DNA mismatch repair protein MutL | Cytoplasmic | WT |
| W6QS64 | PPSAL_0732 | BN5_0738 | UPF0307 protein BN5_0738 | Cytoplasmic | WT |
| W6R0C1 | PPSAL_1255 | BN5_1260 | Uncharacterized protein | Unknown | WT |
| W6R7G4 | PPSAL_3706 | BN5_3763 | Two component LuxR family transcriptional regulator | Cytoplasmic | WT |
| W6QRS4 | PPSAL_0590 | BN5_0595 | Allophanate hydrolase (EC 3.5.1.4) | Cytoplasmic | WT |
| W6R920 | PPSAL_4278 | BN5_4342 | Thioesterase superfamily (EC 3.1.2.-) | Unknown | WT |
| W6R3L3 | PPSAL_4161 | BN5_4224 | Hydrogen peroxide-inducible genes activator | Unknown | WT |
| W6QX12 | PPSAL_2443 | BN5_2473 | Uncharacterized protein | Membrane | WT |
| W6R6F7 | PPSAL_3364 | BN5_3414 | Sensor protein PilS (EC 2.7.13.3) | Membrane | WT |
| W6R3X3 | PPSAL_2487 | BN5_2517 | Uncharacterized protein | Unknown | WT |
| W6QU22 | PPSAL_0853 | BN5_0859 | CreA family protein | Membrane | WT |
| W6RDF9 | PPSAL_1288 | BN5_1293 | Putative transcriptional regulator ycf27 | Cytoplasmic | DapA |
| W6QTK4 | PPSAL_0663 | BN5_0669 | Uncharacterized protein | Unknown | DapA |
| W6RC50 | PPSAL_0834 | BN5_0840 | TetR family transcriptional regulator | Cytoplasmic | DapA |
| W6QVC0 | PPSAL_1820 | BN5_1841 | NADH dehydrogenase I chain M (EC 1.6.5.3) | Membrane | DapA |
| W6R1T7 | PPSAL_4442 | BN5_4506 | ParA family protein | Membrane | DapA |
| W6QU70 | PPSAL_1428 | BN5_1433 | Uncharacterized protein | Unknown | DapA |
| W6R5E4 | PPSAL_3002 | BN5_3042 | XRE family transcriptional regulator | Unknown | DapA |
| W6R189 | PPSAL_4217 | BN5_4281 | Lipoprotein, putative | Unknown | DapA |
| W6QXM2 | PPSAL_2638 | BN5_2672 | Flagellar basal-body rod protein FlgG (Distal rod protein) | Extracellular | DapA |
| W6QXT3 | PPSAL_0364 | BN5_0367 | Extracellular solute-binding protein | Unknown | DapA |
| W6QQN7 | PPSAL_0206 | BN5_0208 | Peptidyl-prolyl cis-trans isomerase (EC 5.2.1.8) | Unknown | DapA |
| W6QUG0 | PPSAL_1518 | BN5_1527 | BolA-like protein 1 | Unknown | DapA |
| W6R1N2 | PPSAL_3584 | BN5_3636 | Uncharacterized protein | Membrane | DapA |
| W6QPZ2 | PPSAL_0393 | BN5_0396 | Uncharacterized protein | Unknown | DapA |
| W6RD68 | PPSAL_1183 | BN5_1187 | Response regulator arlR | Cytoplasmic | DapA |
| W6RLA6 | PPSAL_4062 | BN5_4125 | Membrane protein, putative | Membrane | DapA |
| W6QXX9 | PPSAL_0409 | BN5_0412 | Polyhydroxyalkanoate synthase, class II (EC 2.3.1.-) | Cytoplasmic | DapA |
| W6R0Y8 | PPSAL_3316 | BN5_3363 | Release factor glutamine methyltransferase (RF MTase) (EC 2.1.1.297) (N5-glutamine methyltransferase PrmC) (Protein-(glutamine-N5) MTase PrmC) (Protein-glutamine N-methyltransferase PrmC) | Cytoplasmic | DapA |
| W6R2G5 | PPSAL_1989 | BN5_2014 | LysR family transcriptional regulator | Cytoplasmic | DapA |
| W6RDT1 | PPSAL_1440 | BN5_1445 | Low molecular weight phosphotyrosine protein phosphatase (EC 3.1.3.48) | Cytoplasmic | DapA |
| W6R129 | PPSAL_3371 | BN5_3421 | Type IV pilus assembly protein PilX | Unknown | DapA |
| W6QS06 | PPSAL_0662 | BN5_0668 | Uncharacterized protein | Unknown | DapA |
| W6QTT7 | PPSAL_1291 | BN5_1296 | Acyltransferase (EC 6.2.1.20) | Membrane | DapA |
| W6QPC7 | PPSAL_0174 | BN5_0176 | Mg chelatase, subunit ChlI | Cytoplasmic | DapA |
| W6R110 | PPSAL_1497 | BN5_1506 | Uncharacterized protein | Unknown | DapA |
| W6QV54 | PPSAL_1750 | BN5_1771 | Putative membrane protein | Membrane | DapA |
| W6QVQ6 | PPSAL_1464 | BN5_1469 | Lipoprotein, putative | Unknown | DapA |
| W6QYW4 | PPSAL_2614 | BN5_2648 | Flagellum-specific ATP synthase (EC 3.6.3.14) | Cytoplasmic | DapA |
| W6R0B5 | PPSAL_3553 | BN5_3605 | Aminocarboxymuconate-semialdehyde decarboxylase (EC 4.1.1.45) | Cytoplasmic | DapA |
| W6QWQ3 | PPSAL_1821 | BN5_1842 | NADH dehydrogenase I chain L (EC 1.6.5.3) | Membrane | DapA |
| W6R2Z5 | PPSAL_3987 | BN5_4046 | Uncharacterized protein ybbK | Cytoplasmic | DapA |
| W6QVE0 | PPSAL_2236 | BN5_2264 | MerR family transcriptional regulator | Cytoplasmic | DapA |
| W6QU43 | PPSAL_1396 | BN5_1401 | Uncharacterized protein HI0173 | Unknown | DapA |
| W6QUY8 | PPSAL_2123 | BN5_2149 | Uncharacterized protein | Cytoplasmic | DapA |
| W6R972 | PPSAL_4343 | BN5_4407 | Multidrug resistance protein mdtC | Membrane | DapA |
| W6QW54 | PPSAL_2521 | BN5_2555 | Uncharacterized protein | Cytoplasmic | DapA |
| W6R1U7 | PPSAL_1759 | BN5_1780 | Putative helicase (EC 3.6.1.-) | Cytoplasmic | DapA |
| W6R3A9 | PPSAL_2262 | BN5_2290 | DSBA oxidoreductase | Unknown | DapA |
| W6QV85 | PPSAL_2181 | BN5_2209 | Putative membrane protein | Membrane | DapA |
| W6RGF7 | PPSAL_2375 | BN5_2405 | Blue (Type1) copper domain-containing protein | Unknown | DapA |
| W6RBT3 | PPSAL_0679 | BN5_0685 | PTS system, fructose subfamily, IIC subunit (EC 2.7.1.69) | Membrane | DapA |
| W6QQY1 | PPSAL_0715 | BN5_0721 | Sensor histidine kinase | Membrane | DapA |
| W6R217 | PPSAL_1844 | BN5_1865 | Cold shock-like protein cspG | Cytoplasmic | DapA |
| W6QZ29 | PPSAL_3131 | BN5_3176 | Uncharacterized protein | Cytoplasmic | DapA |
| W6QS91 | PPSAL_0757 | BN5_0763 | CRISPR-associated helicase Cas3 family protein | Unknown | DapA |
| W6QSU8 | PPSAL_1406 | BN5_1411 | Putative competence-damage inducible protein | Unknown | DapA |
| W6R390 | PPSAL_2237 | BN5_2265 | Isovaleryl-CoA dehydrogenase (EC 1.3.8.4) | Cytoplasmic | DapA |
| W6QRS0 | PPSAL_0585 | BN5_0590 | ABC transporter related | Membrane | DapA |
| W6QSC5 | PPSAL_1239 | BN5_1243 | Nuclear receptor binding factor related protein (EC 1.3.1.38) | Cytoplasmic | DapA |
| W6RKZ1 | PPSAL_3948 | BN5_4007 | Uncharacterized protein | Cytoplasmic | DapA |
| W6R067 | PPSAL_3049 | BN5_3092 | Diguanylate cyclase yddV (EC 2.7.7.65) | Cytoplasmic | DapA |
| W6R0B9 | PPSAL_3944 | BN5_4003 | Uncharacterized protein yjbB | Membrane | DapA |
| W6QYQ0 | PPSAL_3008 | BN5_3050 | Methylglyoxal synthase (MGS) (EC 4.2.3.3) | Cytoplasmic | DapA |
| W6R6Z7 | PPSAL_3542 | BN5_3594 | YbaK/prolyl-tRNA synthetase associated region (EC 6.1.1.15) | Cytoplasmic | DapA |
| W6QQD6 | PPSAL_0106 | BN5_0106 | Cytochrome c5-like protein | Periplasmic | DapA |
| W6QZ38 | PPSAL_3522 | BN5_3573 | Ribosomal RNA large subunit methyltransferase H (EC 2.1.1.177) (23S rRNA (pseudouridine1915-N3)-methyltransferase) (23S rRNA m3Psi1915 methyltransferase) (rRNA (pseudouridine-N3-)-methyltransferase RlmH) | Unknown | DapA |
| W6QZM2 | PPSAL_1026 | BN5_1029 | Uncharacterized protein | Cytoplasmic | DapA |
| W6R4D5 | PPSAL_2627 | BN5_2661 | Protein flaG | Unknown | DapA |
| W6R3U8 | PPSAL_4250 | BN5_4314 | Putative oxidoreductase subunit (EC 1.3.99.16) | Cytoplasmic | DapA |
| I7K197 | PPSAL_1722 | BN5_3159 | Uncharacterized protein | Cytoplasmic | DapA |
| W6QZ59 | PPSAL_0846 | BN5_0852 | Pyridoxamine 5'-phosphate oxidase-related, FMN-binding (EC 1.4.3.5) | Cytoplasmic | DapA |
| W6RKX0 | PPSAL_3923 | BN5_3982 | Uncharacterized protein | Unknown | DapA |
| W6R342 | PPSAL_4036 | BN5_4098 | Phosphate acetyl/butaryl transferase (EC 2.3.1.8) | Cytoplasmic | DapA |
| W6QPC3 | PPSAL_0169 | BN5_0171 | ATP-dependent DNA helicase Rep (EC 3.6.4.12) | Cytoplasmic | DapA |
| W6QWP3 | PPSAL_1811 | BN5_1832 | Uncharacterized protein | Unknown | DapA |
| W6QX99 | PPSAL_2891 | BN5_2930 | Multicomponent K+:H+ antiporter subunit C | Membrane | DapA |
| W6R7F3 | PPSAL_3691 | BN5_3748 | UPF0271 protein BN5_3748 | Cytoplasmic | DapA |
| W6RBU3 | PPSAL_0694 | BN5_0700 | Uncharacterized protein | Cytoplasmic | DapA |
| W6R238 | PPSAL_1864 | BN5_1886 | Response regulator in two-component regulatory system | Cytoplasmic | DapA |
| W6R636 | PPSAL_3214 | BN5_3260 | Dual-specificity RNA methyltransferase RlmN (EC 2.1.1.192) (23S rRNA (adenine(2503)-C(2))-methyltransferase) (23S rRNA m2A2503 methyltransferase) (Ribosomal RNA large subunit methyltransferase N) (tRNA (adenine(37)-C(2))-methyltransferase) (tRNA m2A37 methyltransferase) | Cytoplasmic | DapA |
| W6R1D7 | PPSAL_4272 | BN5_4336 | Uncharacterized protein | Cytoplasmic | DapA |
| W6QVW2 | PPSAL_1519 | BN5_1528 | UPF0176 protein BN5_1528 | Cytoplasmic | DapA |
| W6RJH8 | PPSAL_3377 | BN5_3427 | Peptidyl-prolyl cis-trans isomerase (EC 5.2.1.8) | Cytoplasmic | DapA |
| W6RI15 | PPSAL_2840 | BN5_2879 | MaoC-like domain protein | Unknown | DapA |
| W6QYC8 | PPSAL_2424 | BN5_2454 | Coproporphyrinogen-III oxidase (EC 1.3.98.3) | Cytoplasmic | DapA |
| W6QU47 | PPSAL_1828 | BN5_1849 | NADH dehydrogenase I chain E (EC 1.6.5.3) | Cytoplasmic | DapA |
| W6QRF6 | PPSAL_0890 | BN5_0896 | Nitroreductase (EC 1.-.-.-) | Cytoplasmic | DapA |
| W6QZR6 | PPSAL_3705 | BN5_3762 | Polyamine-transporting ATPase (EC 3.6.3.31) | Membrane | DapA |
| W6QYI2 | PPSAL_2938 | BN5_2977 | Endonuclease III (EC 4.2.99.18) (DNA-(apurinic or apyrimidinic site) lyase) | Cytoplasmic | DapA |
| W6QZV3 | PPSAL_2934 | BN5_2973 | Electron transport complex subunit C | Cytoplasmic | DapA |
| W6R1J4 | PPSAL_4000 | BN5_4060 | Glycyl-glycine endopeptidase ALE-1 (EC 3.4.24.75) | OuterMembrane | DapA |
| W6QXQ4 | PPSAL_3056 | BN5_3099 | Uncharacterized protein | Cytoplasmic | DapA |
| W6R0H3 | PPSAL_1315 | BN5_1320 | CAIB/BAIF family protein (EC 2.8.3.16) | Cytoplasmic | DapA |
| W6R3L7 | PPSAL_4166 | BN5_4229 | Uncharacterized protein | Unknown | DapA |
| W6R2Q1 | PPSAL_4379 | BN5_4443 | Nitrate reductase (EC 1.7.1.1) | Unknown | DapA |
| W6R4R5 | PPSAL_2762 | BN5_2801 | RNA polymerase sigma factor RpoS (Sigma S) (Sigma-38) | Cytoplasmic | DapA |
| W6R4S0 | PPSAL_2767 | BN5_2806 | tRNA pseudouridine synthase D (EC 5.4.99.27) (tRNA pseudouridine(13) synthase) (tRNA pseudouridylate synthase D) (tRNA-uridine isomerase D) | Cytoplasmic | DapA |
| W6R1C1 | PPSAL_3466 | BN5_3516 | Uncharacterized protein | Cytoplasmic | DapA |
| W6R2M9 | PPSAL_2044 | BN5_2069 | Uncharacterized protein | Cytoplasmic | DapA |
| W6R0Q8 | PPSAL_0134 | BN5_3719 | Uncharacterized protein | Cytoplasmic | DapA |
| W6RGJ7 | PPSAL_2430 | BN5_2460 | Uncharacterized protein | Cytoplasmic | DapA |
| W6REN3 | PPSAL_1717 | BN5_1738 | Uncharacterized protein | Cytoplasmic | DapA |
| W6QYK5 | PPSAL_3318 | BN5_3365 | Glutamyl-tRNA reductase (GluTR) (EC 1.2.1.70) | Cytoplasmic | DapA |
| W6R6I9 | PPSAL_3394 | BN5_3444 | Response regulator receiver modulated diguanylate cyclase (EC 3.1.1.61) | Cytoplasmic | DapA |
| W6QS32 | PPSAL_0697 | BN5_0703 | Uncharacterized protein | Unknown | DapA |
| W6QZK3 | PPSAL_1006 | BN5_1009 | Putative ATP-binding component of a transport system | Membrane | DapA |
| W6R536 | PPSAL_2897 | BN5_2936 | ProP effector | Unknown | DapA |
| W6RAP3 | PPSAL_0292 | BN5_0295 | Sulfurtransferase FdhD | Cytoplasmic | DapA |
| W6R728 | PPSAL_3567 | BN5_3619 | Acetaldehyde dehydrogenase (EC 1.2.1.10) (Acetaldehyde dehydrogenase [acetylating]) | Unknown | DapA |
| W6QXZ5 | PPSAL_2743 | BN5_2782 | Thiol-disulfide oxidoreductase resA | Periplasmic | DapA |
| W6RC07 | PPSAL_0779 | BN5_0785 | Uncharacterized protein | Unknown | DapA |
| W6QR97 | PPSAL_0420 | BN5_0423 | Nuclease (SNase-like) (EC 3.1.31.1) | Unknown | DapA |
| W6QTY5 | PPSAL_1758 | BN5_1779 | Adenosine monophosphate-protein transferase (EC 2.7.7.n1) (AMPylator) | Cytoplasmic | DapA |
| W6QX98 | PPSAL_0126 | BN5_0125 | GlcG protein | Unknown | DapA |
| W6R863 | #N/A | BN5_4059 | Myocilin | Unknown | DapA |
| W6QY76 | PPSAL_2364 | BN5_2394 | Thioesterase superfamily protein | Cytoplasmic | DapA |
| W6QZ04 | PPSAL_3112 | BN5_3156 | Uncharacterized protein | Unknown | DapA |
| W6R3R6 | PPSAL_4220 | BN5_4284 | Hydrolase, haloacid dehalogenase-like family (EC 3.1.3.18) | Cytoplasmic | DapA |
| W6QRP7 | PPSAL_0052 | BN5_0052 | Uncharacterized protein | Membrane | DapA |
| W6QYQ8 | PPSAL_3018 | BN5_3060 | LysR family transcriptional regulator | Cytoplasmic | DapA |
| W6RDG4 | PPSAL_1293 | BN5_1298 | Solute carrier family 10 member 6 | Membrane | DapA |
| W6QVC5 | PPSAL_1825 | BN5_1846 | NADH-quinone oxidoreductase subunit H (EC 1.6.5.11) (NADH dehydrogenase I subunit H) (NDH-1 subunit H) | Membrane | DapA |
| W6QZJ4 | PPSAL_2829 | BN5_2868 | Uncharacterized protein | Membrane | DapA |
| W6R490 | PPSAL_2587 | BN5_2621 | Uncharacterized protein | Unknown | DapA |
| W6R194 | PPSAL_3891 | BN5_3950 | Uncharacterized protein | Periplasmic | DapA |
| W6R844 | PPSAL_3975 | BN5_4034 | Uncharacterized protein | Membrane | DapA |
| W6QXB2 | PPSAL_2906 | BN5_2945 | Cation antiporter | Membrane | DapA |
| W6R3M9 | PPSAL_2402 | BN5_2432 | TetR family transcriptional regulator | Unknown | DapA |
| W6QY34 | PPSAL_2313 | BN5_2344 | Metallo-lactamase | Unknown | DapA |
| W6QW58 | PPSAL_1630 | BN5_1642 | YecA family protein | Cytoplasmic | DapA |
| W6RBI1 | PPSAL_0592 | BN5_0597 | Integral membrane sensor hybrid histidine kinase (EC 2.7.13.3) | Membrane | DapA |
| W6R3E6 | PPSAL_2306 | BN5_2337 | Coenzyme PQQ synthesis protein E (Pyrroloquinoline quinone biosynthesis protein E) | Cytoplasmic | DapA |
| W6R016 | PPSAL_3829 | BN5_3888 | Diguanylate cyclase | Membrane | DapA |
| W6QWN7 | PPSAL_2661 | BN5_2696 | HTH-type transcriptional regulator betI | Cytoplasmic | DapA |
| W6RJC3 | PPSAL_3307 | BN5_3354 | Short-chain dehydrogenase/reductase SDR (EC 1.1.1.100) | Unknown | DapA |
| W6QW36 | PPSAL_1605 | BN5_1617 | Putative glutamine amidotransferase yafJ (EC 2.4.2.-) | Cytoplasmic | DapA |
| W6R4P8 | PPSAL_2742 | BN5_2781 | Cytochrome c-type protein | Membrane | DapA |
| W6QY43 | PPSAL_2323 | BN5_2354 | ABC transporter periplasmic protein | Unknown | DapA |
| W6RCP9 | PPSAL_1058 | BN5_1062 | UPF0135 protein | Unknown | DapA |
| W6RH10 | PPSAL_2605 | BN5_2639 | Flagellar protein | Membrane | DapA |
| W6QUU4 | PPSAL_1142 | BN5_1146 | Uncharacterized protein | Membrane | DapA |
| W6R0H7 | PPSAL_1320 | BN5_1325 | Uncharacterized protein | Unknown | DapA |
| W6RH20 | PPSAL_2615 | BN5_2649 | Flagellar assembly protein H | Cytoplasmic | DapA |
| W6QYL3 | PPSAL_0619 | BN5_0624 | Lipoprotein, putative | Periplasmic | DapA |
| W6RJ69 | PPSAL_3237 | BN5_3284 | Permease YjgP/YjgQ family protein | Membrane | DapA |
| W6R4A2 | PPSAL_2597 | BN5_2631 | RNA polymerase sigma factor FliA (RNA polymerase sigma factor for flagellar operon) (Sigma F) (Sigma-28) | Cytoplasmic | DapA |
| W6QW16 | PPSAL_2095 | BN5_2121 | Putative nitrate transporter narT | Membrane | DapA |
| W6QY70 | PPSAL_0494 | BN5_0497 | 3-deoxy-D-manno-octulosonic-acid transferase (EC 2.-.-.-) | Unknown | DapA |
| W6QSL5 | PPSAL_0366 | BN5_0369 | ATP-dependent RNA helicase | Unknown | DapA |
| W6QXL9 | PPSAL_2633 | BN5_2667 | Flagellin B | Extracellular | DapA |
| W6QWK0 | PPSAL_2273 | BN5_2301 | Furoyl-CoA synthetase | Cytoplasmic | DapA |
| W6QXZ2 | PPSAL_3129 | BN5_3174 | Uncharacterized protein | Unknown | DapA |
| W6RG31 | PPSAL_2220 | BN5_2248 | Uncharacterized protein | Cytoplasmic | DapA |
| W6RA04 | PPSAL_0078 | BN5_0078 | LysR family transcriptional regulator | Cytoplasmic | DapA |
| W6QVB1 | PPSAL_1810 | BN5_1831 | Uncharacterized protein | OuterMembrane | DapA |
| W6R9F0 | PPSAL_4418 | BN5_4482 | Transposition protein TniB | Cytoplasmic | DapA |
| W6R1G3 | PPSAL_3511 | BN5_3561 | Transcriptional regulator | Unknown | DapA |
| W6QWE5 | PPSAL_2616 | BN5_2650 | Flagellar motor switch protein FliG | Cytoplasmic | DapA |
| W6QXC2 | PPSAL_2543 | BN5_2577 | Membrane protein, putative | Membrane | DapA |
| W6QWB0 | PPSAL_2178 | BN5_2206 | Acyl-CoA dehydrogenase family member 11 | Cytoplasmic | DapA |
| W6QSP3 | PPSAL_0907 | BN5_0913 | Uncharacterized protein | Cytoplasmic | DapA |
| W6R0P3 | PPSAL_4063 | BN5_4126 | Glycosyl transferase, group 1 family protein (EC 2.4.1.-) | Cytoplasmic | DapA |
| W6QRT0 | PPSAL_0092 | BN5_0092 | Cytochrome c oxidase subunit 2 (EC 1.9.3.1) | Membrane | DapA |
| W6QTY0 | PPSAL_1753 | BN5_1774 | Methyl-accepting chemotaxis protein tlpB | Membrane | DapA |
| W6QXF6 | PPSAL_2946 | BN5_2985 | Uncharacterized protein | Cytoplasmic | DapA |
| W6QS97 | PPSAL_1204 | BN5_1208 | Lipoprotein, putative | Unknown | DapA |
| W6QS95 | PPSAL_0762 | BN5_0768 | CRISPR-associated Cse1 family protein | Unknown | DapA |
| W6QS82 | PPSAL_1189 | BN5_1193 | Uncharacterized protein | Cytoplasmic | DapA |
| W6QTT9 | PPSAL_0763 | BN5_0769 | CRISPR-associated protein, Cse2 family | Unknown | DapA |
| W6QR92 | PPSAL_0415 | BN5_0418 | Uncharacterized protein | Cytoplasmic | DapA |
| W6R0Y2 | PPSAL_4133 | BN5_4196 | Uncharacterized protein | Cytoplasmic | DapA |
| W6QR33 | PPSAL_0765 | BN5_0771 | CRISPR-associated Cas5e family protein | Unknown | DapA |
| W6QWG1 | PPSAL_2626 | BN5_2660 | Flagellar hook-associated protein 2 (HAP2) (Flagellar cap protein) | Extracellular | DapA |
| W6RJJ1 | PPSAL_3392 | BN5_3442 | Mechanosensitive ion channel | Membrane | DapA |
| W6QXL0 | PPSAL_2135 | BN5_2162 | Putative ABC transporter permease ybbP | Membrane | DapA |
| W6R1I7 | PPSAL_3539 | BN5_3591 | Uncharacterized protein | Membrane | DapA |
| W6QQV6 | PPSAL_0285 | BN5_0288 | DNA helicase (EC 3.6.1.-) | Cytoplasmic | DapA |
| W6RGB7 | PPSAL_2324 | BN5_2355 | Beta-propeller repeat protein | Unknown | DapA |
| W6QXS3 | PPSAL_2189 | BN5_2217 | Ubiquinone/menaquinone biosynthesis methyltransferase ubiE (EC 2.1.1.-) | Unknown | DapA |
| W6RBT9 | PPSAL_0689 | BN5_0695 | PKHD-type hydroxylase BN5_0695 (EC 1.14.11.-) | Cytoplasmic | DapA |
| W6RBV6 | PPSAL_0714 | BN5_0720 | Uncharacterized protein | Unknown | DapA |
| W6R0T5 | PPSAL_3251 | BN5_3298 | Uncharacterized protein | Cytoplasmic | DapA |
| W6R410 | PPSAL_2517 | BN5_2551 | 1,2-dihydroxy-3-keto-5-methylthiopentene dioxygenase (EC 1.13.11.54) | Cytoplasmic | DapA |
| W6R835 | PPSAL_3965 | BN5_4024 | Uncharacterized membrane protein NMB1645 | Membrane | DapA |
| W6QPB4 | PPSAL_0159 | BN5_0161 | FAD dependent oxidoreductase (EC 1.-.-.-) | Cytoplasmic | DapA |
| W6R2I8 | PPSAL_2009 | BN5_2034 | DNA-binding transcriptional activator OsmE | Unknown | DapA |
| W6QY36 | PPSAL_2788 | BN5_2827 | Zinc metalloprotease (EC 3.4.24.-) | Membrane | DapA |
| W6QXF1 | PPSAL_2941 | BN5_2980 | OmpA/MotB domain-containing protein | OuterMembrane | DapA |
| W6QZ57 | PPSAL_3541 | BN5_3593 | Beta-lactamase domain-containing protein | Cytoplasmic | DapA |
| W6QVJ4 | PPSAL_1404 | BN5_1409 | Ferredoxin-2 | Cytoplasmic | DapA |
| W6RGZ5 | PPSAL_2590 | BN5_2624 | ParA family protein | Unknown | DapA |
| W6RG48 | PPSAL_2240 | BN5_2268 | 3-methylcrotonoyl-CoA carboxylase, alpha subunit (Putative) (EC 6.4.1.4) | Cytoplasmic | DapA |
| W6QVH5 | PPSAL_1880 | BN5_1903 | Cytochrome d ubiquinol oxidase, subunit II (EC 1.10.3.-) | Membrane | DapA |
| W6R243 | PPSAL_1869 | BN5_1892 | Uncharacterized HTH-type transcriptional regulator ywbI | Cytoplasmic | DapA |
| W6R3B2 | PPSAL_4101 | BN5_4164 | Putative NAD(P)H-dependent FMN reductase | Cytoplasmic | DapA |
| W6RA28 | PPSAL_0103 | BN5_0103 | Uncharacterized protein | Unknown | DapA |
| W6RKJ9 | PPSAL_3714 | BN5_3771 | 4-hydroxythreonine-4-phosphate dehydrogenase (EC 1.1.1.262) (4-(phosphohydroxy)-L-threonine dehydrogenase) | Cytoplasmic | DapA |
| W6QWH4 | PPSAL_2636 | BN5_2670 | Flagellar P-ring protein (Basal body P-ring protein) | Periplasmic | DapA |
| W6R5U2 | PPSAL_3125 | BN5_3170 | GTPase subunit of restriction endonucleas | Cytoplasmic | DapA |
| W6RA91 | PPSAL_0129 | BN5_0128 | Glycolate oxidase, subunit GlcD (EC 1.1.3.15) | Cytoplasmic | DapA |
| W6R252 | PPSAL_4175 | BN5_4238 | Probable chorismate pyruvate-lyase (CL) (CPL) (EC 4.1.3.40) | Cytoplasmic | DapA |
| W6R0E3 | PPSAL_3969 | BN5_4028 | Prolipoprotein diacylglyceryl transferase (EC 2.4.99.-) | Membrane | DapA |
| W6QY98 | PPSAL_2853 | BN5_2892 | Carboxy-S-adenosyl-L-methionine synthase (Cx-SAM synthase) (EC 2.1.3.-) | Cytoplasmic | DapA |
| W6QSG9 | PPSAL_1269 | BN5_1274 | Ecotin | Periplasmic | DapA |
| W6QY47 | PPSAL_2328 | BN5_2359 | DNA-binding response regulator | Cytoplasmic | DapA |
| W6R6E9 | PPSAL_3354 | BN5_3404 | Sulfate transporter CysZ | Membrane | DapA |
| W6QT03 | PPSAL_1456 | BN5_1461 | Thymidylate kinase (EC 2.7.4.9) (dTMP kinase) | Cytoplasmic | DapA |
| W6QVF7 | PPSAL_1367 | BN5_1372 | Chemotaxis protein CheV (EC 2.7.3.-) | Cytoplasmic | DapA |
| W6QXK8 | PPSAL_0269 | BN5_0272 | Phosphate transporter | Membrane | DapA |
| W6QXL4 | PPSAL_2628 | BN5_2662 | Flagellin | Extracellular | DapA |
| W6QYQ3 | PPSAL_3013 | BN5_3055 | Deaminase-reductase domain-containing protein | Cytoplasmic | DapA |
| W6R372 | PPSAL_4066 | BN5_4129 | Glycosyl transferase, group 1 family protein | Cytoplasmic | DapA |
| W6QY59 | PPSAL_3154 | BN5_3199 | Fatty acid metabolism regulator protein | Cytoplasmic | DapA |
| W6RK00 | PPSAL_3531 | BN5_3582 | Dehydratase | Unknown | DapA |
| W6R1M5 | PPSAL_4372 | BN5_4436 | Zinc transport protein zntB | Membrane | DapA |
| W6QPN8 | PPSAL_0293 | BN5_0296 | LysR family transcriptional regulator | Cytoplasmic | DapA |
| W6QZ01 | PPSAL_0766 | BN5_0772 | CRISPR-associated Cse3 family protein | Unknown | DapA |
| W6RBZ4 | PPSAL_0764 | BN5_0770 | Uncharacterized protein | Cytoplasmic | DapA |
| W6QU06 | PPSAL_1355 | BN5_1361 | Glycerol-3-phosphate acyltransferase (GPAT) (EC 2.3.1.15) | Membrane | DapA |
| W6RDB7 | PPSAL_1243 | BN5_1247 | Conserved virulence factor B | Cytoplasmic | DapA |
| W6R063 | PPSAL_3884 | BN5_3943 | Glutathione-independent formaldehyde dehydrogenase (EC 1.2.1.46) | Cytoplasmic | DapA |
| W6QRT1 | PPSAL_0595 | BN5_0600 | DNA-binding protein Fis | Cytoplasmic | DapA |
| W6QZI2 | PPSAL_3255 | BN5_3302 | Uncharacterized protein | Unknown | DapA |
| W6RGQ2 | PPSAL_2485 | BN5_2515 | Uncharacterized protein | Cytoplasmic | DapA |
| W6QZA1 | PPSAL_3166 | BN5_3211 | RecBCD enzyme subunit RecB (EC 3.1.11.5) (Exonuclease V subunit RecB) (ExoV subunit RecB) (Helicase/nuclease RecBCD subunit RecB) | Cytoplasmic | DapA |
| W6QU19 | PPSAL_1371 | BN5_1376 | Uncharacterized protein | Cytoplasmic | DapA |
| W6QXI4 | PPSAL_2976 | BN5_3016 | Glycine cleavage system H protein | Unknown | DapA |
| W6R0U0 | PPSAL_1432 | BN5_1437 | Lipoprotein-releasing system transmembrane protein lolC | Membrane | DapA |
| W6RI82 | PPSAL_2915 | BN5_2954 | Uncharacterized protein | Unknown | DapA |
| W6RM49 | PPSAL_4386 | BN5_4450 | AraC family transcriptional regulator | Cytoplasmic | DapA |
| W6QYQ6 | PPSAL_3383 | BN5_3433 | Uncharacterized protein | Unknown | DapA |
| W6QXA8 | PPSAL_2031 | BN5_2056 | Putative CheW protein (EC 2.7.13.3) | Cytoplasmic | DapA |
| W6QSX2 | PPSAL_1426 | BN5_1431 | Uncharacterized protein | Unknown | DapA |
| W6QYH6 | PPSAL_3283 | BN5_3330 | Arginine/ornithine antiporter | Membrane | DapA |
| W6RLL0 | PPSAL_4142 | BN5_4205 | DNA repair protein radC homolog | Cytoplasmic | DapA |
| W6RJD8 | PPSAL_3327 | BN5_3376 | Uncharacterized protein | Cytoplasmic | DapA |
| W6RL16 | PPSAL_3973 | BN5_4032 | RNA pyrophosphohydrolase (EC 3.6.1.-) ((Di)nucleoside polyphosphate hydrolase) | Cytoplasmic | DapA |
| W6R179 | PPSAL_3421 | BN5_3471 | Transcriptional regulator MraZ | Cytoplasmic | DapA |
| W6QXW1 | PPSAL_2229 | BN5_2257 | PilZ domain protein | Cytoplasmic | DapA |
| W6QY30 | PPSAL_2308 | BN5_2339 | Pyrroloquinoline-quinone synthase (EC 1.3.3.11) (Coenzyme PQQ synthesis protein C) (Pyrroloquinoline quinone biosynthesis protein C) | Cytoplasmic | DapA |
| W6R7E5 | PPSAL_3681 | BN5_3738 | ATPase central domain-containing protein (EC 3.6.4.6) | Membrane | DapA |
| W6QWY9 | PPSAL_2423 | BN5_2453 | Uncharacterized protein | Membrane | DapA |
| W6RJ57 | PPSAL_3217 | BN5_3263 | 2Fe-2S ferredoxin | Cytoplasmic | DapA |
| W6QU36 | PPSAL_1813 | BN5_1834 | Multidrug resistance protein mdtA | Membrane | DapA |
| W6R3W1 | PPSAL_2477 | BN5_2507 | Uncharacterized protein | Unknown | DapA |
| W6QQ96 | PPSAL_0508 | BN5_0511 | Chemotaxis protein | Membrane | DapA |
| W6QWL9 | PPSAL_2292 | BN5_2322 | MerR family transcriptional regulator | Cytoplasmic | DapA |
| W6QYE8 | PPSAL_2903 | BN5_2942 | Multicomponent Na+:H+ antiporter subunit A (EC 1.6.99.5) | Membrane | DapA |
| W6RAZ0 | PPSAL_0412 | BN5_0415 | Putative phosphoribosylformimino-5-aminoimidazole carboxamide ribotide isomerase (EC 5.3.1.16) | Unknown | DapA |
| W6RKX4 | PPSAL_3928 | BN5_3987 | Cell division protein FtsX | Membrane | DapA |
| W6QXH0 | PPSAL_2588 | BN5_2622 | Chemotaxis protein cheW | Cytoplasmic | DapA |
| W6R080 | PPSAL_3515 | BN5_3565 | Octanoyltransferase (EC 2.3.1.181) (Lipoate-protein ligase B) (Lipoyl/octanoyl transferase) (Octanoyl-[acyl-carrier-protein]-protein N-octanoyltransferase) | Cytoplasmic | DapA |
| W6RDS2 | PPSAL_1430 | BN5_1435 | Lipoprotein-releasing system transmembrane protein lolC | Membrane | DapA |
| W6QSS8 | PPSAL_0942 | BN5_0950 | Glutamyl-Q tRNA(Asp) synthetase (Glu-Q-RSs) (EC 6.1.1.-) | Cytoplasmic | DapA |
| W6R532 | PPSAL_2892 | BN5_2931 | Multicomponent K+:H+ antiporter subunit Amulticomponent K+:H+ antiporter subunit A (EC 1.6.99.5) | Membrane | DapA |
| W6R3L4 | PPSAL_2387 | BN5_2417 | TonB-dependent siderophore receptor | OuterMembrane | DapA |
| W6R8F1 | PPSAL_4079 | BN5_4142 | C4-dicarboxylate transporter, putative | Membrane | DapA |
| W6R268 | PPSAL_3738 | BN5_3796 | Uncharacterized protein | Unknown | DapA |
| W6RKU6 | PPSAL_3883 | BN5_3942 | Acyltransferase family protein (EC 2.7.7.19) | Membrane | DapA |
| W6RAV7 | PPSAL_0377 | BN5_0380 | Uncharacterized protein | Unknown | DapA |
| W6QZU3 | PPSAL_1090 | BN5_1094 | Lipoprotein, putative | Unknown | DapA |
| W6R6P1 | PPSAL_3449 | BN5_3499 | BolA family protein | Unknown | DapA |
| W6R1B4 | PPSAL_3911 | BN5_3970 | Uncharacterized protein | Membrane | DapA |
| W6R0P5 | PPSAL_1390 | BN5_1395 | Na(+)-translocating NADH-quinone reductase subunit B (Na(+)-NQR subunit B) (Na(+)-translocating NQR subunit B) (EC 1.6.5.8) (NQR complex subunit B) (NQR-1 subunit B) | Membrane | DapA |
| W6QZX0 | PPSAL_3770 | BN5_3828 | Uncharacterized protein | Unknown | DapA |
| W6QXY2 | PPSAL_2249 | BN5_2277 | Methyl-accepting chemotaxis sensory transducer | Membrane | DapA |
| W6QQE4 | PPSAL_0558 | BN5_0563 | Uncharacterized protein | Unknown | DapA |
| W6QRI7 | PPSAL_0920 | BN5_0928 | Ribosome maturation factor RimP | Cytoplasmic | DapA |
| W6RE32 | PPSAL_1541 | BN5_1552 | Uncharacterized protein | Unknown | DapA |
| W6RJB1 | PPSAL_3292 | BN5_3339 | Uncharacterized protein | OuterMembrane | DapA |
| W6QV93 | PPSAL_1790 | BN5_1811 | Uncharacterized protein | Unknown | DapA |
| W6RL10 | PPSAL_3968 | BN5_4027 | Thymidylate synthase (TS) (TSase) (EC 2.1.1.45) | Cytoplasmic | DapA |
| W6R199 | PPSAL_3896 | BN5_3955 | 23S rRNA methyltransferase (EC 2.1.1.-) | Unknown | DapA |
| W6RKT9 | PPSAL_3873 | BN5_3932 | Uncharacterized protein | Membrane | DapA |
| W6R213 | PPSAL_3683 | BN5_3740 | 2-nonaprenyl-3-methyl-6-methoxy-1,4-benzoquinol hydroxylase (EC 1.14.13.-) (5-demethoxyubiquinone hydroxylase) (DMQ hydroxylase) | Unknown | DapA |
| W6QYD7 | PPSAL_0549 | BN5_0554 | Urease accessory protein UreE | Cytoplasmic | DapA |
| W6R0C5 | PPSAL_3563 | BN5_3615 | Alpha/beta hydrolase fold (EC 3.7.1.-) | Extracellular | DapA |
| W6QSY7 | PPSAL_1441 | BN5_1446 | UDP-N-acetylenolpyruvoylglucosamine reductase (EC 1.3.1.98) (UDP-N-acetylmuramate dehydrogenase) | Cytoplasmic | DapA |
| W6R3V4 | PPSAL_4255 | BN5_4319 | High-affinity zinc uptake system protein znuA | Periplasmic | DapA |
| W6R9A9 | PPSAL_4378 | BN5_4442 | Uncharacterized protein | Unknown | DapA |
| W6RIW6 | PPSAL_3128 | BN5_3173 | Uncharacterized protein | Unknown | DapA |
| W6R427 | PPSAL_2532 | BN5_2566 | Uncharacterized HTH-type transcriptional regulator yncC | Cytoplasmic | DapA |
| W6R360 | PPSAL_2197 | BN5_2225 | Peptide ABC transporter,putative ATP-binding protein (EC 3.6.3.25) | Membrane | DapA |
| W6R277 | PPSAL_4204 | BN5_4268 | Breakpoint cluster region protein (EC 2.7.11.1) | Cytoplasmic | DapA |
| W6QX73 | PPSAL_2498 | BN5_2532 | Putative GAF sensor protein | Unknown | DapA |
| W6R122 | PPSAL_3361 | BN5_3411 | Pseudouridine synthase (EC 5.4.99.-) | Cytoplasmic | DapA |
| W6RLQ2 | PPSAL_4201 | BN5_4265 | Protein SFI1 homolog | Unknown | DapA |
| W6R517 | PPSAL_2877 | BN5_2916 | Two component transcriptional regulator | Cytoplasmic | DapA |
| W6R0A2 | PPSAL_3083 | BN5_3127 | Phosphoheptose isomerase (EC 5.3.1.28) (Sedoheptulose 7-phosphate isomerase) | Cytoplasmic | DapA |
| W6R1T1 | PPSAL_1739 | BN5_1760 | tRNA-hydroxylase (EC 1.-.-.-) | Cytoplasmic | DapA |
| W6QPJ5 | PPSAL_0248 | BN5_0251 | Glycine cleavage system H protein | Unknown | DapA |
| W6QVV7 | PPSAL_2411 | BN5_2441 | Cytochrome c oxidase subunit 1 homolog, bacteroid (EC 1.9.3.1) | Membrane | DapA |
| W6QZT3 | PPSAL_1080 | BN5_1084 | Uncharacterized protein | Cytoplasmic | DapA |
| W6QUS0 | PPSAL_1132 | BN5_1136 | Putative signaling protein CC_0091 | Membrane | DapA |
| W6R382 | PPSAL_2227 | BN5_2255 | Anti-sigma factor antagonist | Cytoplasmic | DapA |
| W6QSF2 | PPSAL_0301 | BN5_0304 | Uracil phosphoribosyltransferase (EC 2.4.2.9) | Cytoplasmic | DapA |
| W6RLL5 | PPSAL_4152 | BN5_4215 | Ribonuclease PH (RNase PH) (EC 2.7.7.56) (tRNA nucleotidyltransferase) | Cytoplasmic | DapA |
| W6QUL1 | PPSAL_2003 | BN5_2028 | Periplasmic binding protein/LacI transcriptional regulator | Unknown | DapA |
| W6R4B8 | PPSAL_2612 | BN5_2646 | STAS domain protein | Unknown | DapA |
| W6QPN0 | PPSAL_0283 | BN5_0286 | 33 kDa chaperonin (Heat shock protein 33 homolog) (HSP33) | Cytoplasmic | DapA |
| W6QP02 | PPSAL_0059 | BN5_0059 | Aldose 1-epimerase family protein (EC 4.2.1.9) | Cytoplasmic | DapA |
| W6QXR7 | PPSAL_0349 | BN5_0352 | Uncharacterized protein | Unknown | DapA |
| W6QZB2 | PPSAL_3176 | BN5_3221 | Uncharacterized protein | Unknown | DapA |
| W6RC78 | PPSAL_0874 | BN5_0880 | Regulatory protein, LuxR | Unknown | DapA |
| W6RB89 | PPSAL_0507 | BN5_0510 | Chemotaxis protein | Membrane | DapA |
| W6QTI6 | PPSAL_0636 | BN5_0641 | DNA repair protein RadA | Cytoplasmic | DapA |
| W6R2X1 | PPSAL_2124 | BN5_2150 | Cytosolic 5'-nucleotidase 1 (EC 3.1.3.5) | Cytoplasmic | DapA |
| W6RKS8 | PPSAL_3858 | BN5_3917 | Biotin synthase (EC 2.8.1.6) | Cytoplasmic | DapA |
| W6RI39 | PPSAL_2860 | BN5_2899 | Ribonuclease 3 (EC 3.1.26.3) (Ribonuclease III) (RNase III) | Cytoplasmic | DapA |
| W6R1N4 | PPSAL_4045 | BN5_4107 | Putative ABC transporter ATP-binding protein yhiH | Membrane | DapA |
| W6QZU5 | PPSAL_3380 | BN5_3430 | Riboflavin biosynthesis protein (EC 2.7.1.26) (EC 2.7.7.2) | Cytoplasmic | DapA |
| W6QRM5 | PPSAL_0032 | BN5_0032 | Oxygen-dependent coproporphyrinogen-III oxidase (CPO) (Coprogen oxidase) (Coproporphyrinogenase) (EC 1.3.3.3) | Cytoplasmic | DapA |
| W6R0Q3 | PPSAL_3216 | BN5_3262 | Uncharacterized protein | Unknown | DapA |
| W6R8U0 | PPSAL_4188 | BN5_4252 | NAD(P) transhydrogenase subunit beta (EC 1.6.1.2) (Nicotinamide nucleotide transhydrogenase subunit beta) | Membrane | DapA |
| W6QX57 | PPSAL_0110 | BN5_0110 | Diguanylate cyclase | Cytoplasmic | DapA |
| W6QYX6 | PPSAL_3453 | BN5_3503 | Uncharacterized protein | Membrane | DapA |
| W6R2Y2 | PPSAL_2133 | BN5_2160 | Arylesterase (EC 3.1.2.-) | Unknown | DapA |
| W6RKG4 | PPSAL_3660 | BN5_3716 | Type III pantothenate kinase (EC 2.7.1.33) (PanK-III) (Pantothenic acid kinase) | Cytoplasmic | DapA |
| W6QTH7 | PPSAL_1166 | BN5_1170 | D-erythro-7,8-dihydroneopterin tri P epimerase (EC 5.-.-.-) | Cytoplasmic | DapA |
| W6QZL6 | PPSAL_3300 | BN5_3347 | NAD-dependent epimerase/dehydratase | Cytoplasmic | DapA |
| W6RFY0 | PPSAL_2161 | BN5_2188 | UPF0345 protein BN5_2188 | Unknown | DapA |
| W6R205 | PPSAL_3673 | BN5_3730 | Uncharacterized protein | Cytoplasmic | DapA |
| W6QUW1 | PPSAL_2103 | BN5_2129 | TM helix repeat-containing protein | Membrane | DapA |
| W6R3Q4 | PPSAL_4210 | BN5_4274 | Sialic acid TRAP transporter permease protein siaT | Membrane | DapA |
| W6QZN6 | PPSAL_3320 | BN5_3367 | Outer-membrane lipoprotein LolB | Unknown | DapA |
| W6RGI6 | PPSAL_2415 | BN5_2445 | Cytochrome c oxidase, cbb3-type, subunit I (EC 1.9.3.1) | Membrane | DapA |
| W6R347 | PPSAL_2177 | BN5_2205 | Hydroxysteroid dehydrogenase-like protein 2 | Unknown | DapA |
| W6R9Y3 | PPSAL_0058 | BN5_0058 | TRAP dicarboxylate transporter, DctM subunit | Membrane | DapA |
| W6QR20 | PPSAL_0345 | BN5_0348 | Lipoprotein, putative | Unknown | DapA |
| W6R5K9 | PPSAL_3052 | BN5_3095 | Histidine triad (HIT) protein | Unknown | DapA |
| W6QSF5 | PPSAL_0822 | BN5_0828 | Adenine deaminase (ADE) (EC 3.5.4.2) (Adenine aminohydrolase) (AAH) | Cytoplasmic | DapA |
| W6QYC5 | PPSAL_2419 | BN5_2449 | Cytochrome c oxidase accessory protein, cbb3-type, CcoG | Membrane | DapA |
| W6RJQ9 | PPSAL_3452 | BN5_3502 | Uncharacterized protein | Unknown | DapA |
| W6QWD3 | PPSAL_1686 | BN5_1697 | Uncharacterized protein | Unknown | DapA |
| W6R1V8 | PPSAL_1774 | BN5_1795 | Glycerol-3-phosphate dehydrogenase [NAD(P)+] (EC 1.1.1.94) (NAD(P)H-dependent glycerol-3-phosphate dehydrogenase) | Cytoplasmic | DapA |
| W6RBJ3 | PPSAL_0602 | BN5_0607 | Thiol:disulfide interchange protein DsbD (EC 1.8.1.8) (Protein-disulfide reductase) (Disulfide reductase) | Membrane | DapA |
| W6R1Q8 | PPSAL_4070 | BN5_4133 | Mannose-1-phosphate guanylyltransferase/mannose-6-phosphate isomerase (EC 2.7.7.22) | Cytoplasmic | DapA |
| I7K7G1 | PPSAL_0639 | BN5_2103 | Transposase for insertion sequence element IS200 | Cytoplasmic | DapA |
| W6RLS9 | PPSAL_4231 | BN5_4295 | Uncharacterized protein | Unknown | DapA |
| W6QU30 | PPSAL_0863 | BN5_0869 | Methyl-accepting chemotaxis sensory transducer | Membrane | DapA |
| W6QR86 | PPSAL_0410 | BN5_0413 | Poly(3-hydroxyalkanoate) depolymerase (EC 3.1.1.-) | Unknown | DapA |
| W6R7H3 | PPSAL_3716 | BN5_3773 | Protein ApaG | Unknown | DapA |
| W6RJ65 | PPSAL_3227 | BN5_3273 | Uncharacterized protein | Unknown | DapA |
| W6R094 | PPSAL_1215 | BN5_1219 | Periplasmic beta-glucosidase (EC 3.2.1.21) | Periplasmic | DapA |
| W6QR22 | PPSAL_0755 | BN5_0761 | Acyl-CoA dehydrogenase domain-containing protein | Cytoplasmic | DapA |
| W6RI74 | PPSAL_2905 | BN5_2944 | Putative multicomponent Na+:H+ antiporter subunit D (EC 1.6.99.5) | Membrane | DapA |
| W6QUI6 | PPSAL_1544 | BN5_1555 | Histidine kinase (EC 2.7.13.3) | Membrane | DapA |
| W6QVH1 | PPSAL_1875 | BN5_1898 | 2-dehydro-3-deoxyphosphogluconate aldolase/4-hydroxy-2-oxoglutarate aldolase | Cytoplasmic | DapA |
| W6R7H6 | PPSAL_3721 | BN5_3778 | SpoVR family protein | Cytoplasmic | DapA |
| W6QSZ2 | PPSAL_0491 | BN5_0494 | ADP-ribose pyrophosphatase (EC 3.6.1.13) | Cytoplasmic | DapA |
| W6QR81 | PPSAL_0405 | BN5_0408 | Putative polyhydroxyalkanoic acid system protein | Cytoplasmic | DapA |
| W6R1Z9 | PPSAL_1824 | BN5_1845 | NADH-quinone oxidoreductase subunit I (EC 1.6.5.11) (NADH dehydrogenase I subunit I) (NDH-1 subunit I) | Unknown | DapA |
| W6QXW7 | PPSAL_2713 | BN5_2749 | 2,4-dienoyl-coa reductase FADH1, putative (EC 1.3.1.34) | Cytoplasmic | DapA |
| W6RKJ0 | PPSAL_3694 | BN5_3751 | Biotin carboxylase/biotin carboxyl carrier protein (EC 6.3.4.14) | Cytoplasmic | DapA |
| W6R0A5 | PPSAL_3929 | BN5_3988 | Methionine import ATP-binding protein metN (EC 3.6.3.-) | Membrane | DapA |
| W6QYD0 | PPSAL_3228 | BN5_3274 | Protein-export membrane protein SecF | Membrane | DapA |
| W6QTE5 | PPSAL_1602 | BN5_1614 | Oligopeptidase B (EC 3.4.21.83) | Periplasmic | DapA |
| W6QY04 | PPSAL_2274 | BN5_2302 | 2-oxoglutaroyl-CoA hydrolase | Cytoplasmic | DapA |
| W6R151 | PPSAL_4168 | BN5_4231 | Rubredoxin reductase (EC 1.18.1.1) | Cytoplasmic | DapA |
| W6QPD6 | PPSAL_0189 | BN5_0191 | Protein CyaY | Cytoplasmic | DapA |
| W6R1F7 | PPSAL_4292 | BN5_4356 | Antitoxin | Unknown | DapA |
| W6RGY5 | PPSAL_2580 | BN5_2614 | Cytochrome c-type biogenesis protein CcmE (Cytochrome c maturation protein E) (Heme chaperone CcmE) | Unknown | DapA |
| W6RF01 | PPSAL_1857 | BN5_1878 | Putative glutathione S-transferase (EC 2.5.1.18) | Cytoplasmic | DapA |
| W6QT60 | PPSAL_1071 | BN5_1075 | tRNA (guanine-N(1)-)-methyltransferase (EC 2.1.1.228) (M1G-methyltransferase) (tRNA [GM37] methyltransferase) | Cytoplasmic | DapA |
| W6RFE4 | PPSAL_2012 | BN5_2037 | Fe/S biogenesis protein NfuA | Cytoplasmic | DapA |
| W6R2G2 | PPSAL_4299 | BN5_4363 | Uncharacterized protein | Unknown | DapA |
| W6QXZ6 | PPSAL_2264 | BN5_2292 | TetR family transcriptional regulator | Cytoplasmic | DapA |
| W6QZE1 | PPSAL_3611 | BN5_3663 | NAD-dependent epimerase/dehydratase (EC 5.1.3.2) | Cytoplasmic | DapA |
| W6QVF9 | PPSAL_2256 | BN5_2284 | 6-carboxy-5,6,7,8-tetrahydropterin synthase (EC 4.-.-.-) | Cytoplasmic | DapA |
| W6R8E4 | PPSAL_4074 | BN5_4137 | dTDP-4-dehydrorhamnose 3,5-epimerase (EC 5.1.3.13) | Unknown | DapA |
| W6RJM3 | PPSAL_3422 | BN5_3472 | Ribosomal RNA small subunit methyltransferase I (EC 2.1.1.198) (16S rRNA 2'-O-ribose C1402 methyltransferase) (rRNA (cytidine-2'-O-)-methyltransferase RsmI) | Cytoplasmic | DapA |
| W6RAY1 | PPSAL_0402 | BN5_0405 | Probable protein kinase UbiB (EC 2.7.-.-) (Ubiquinone biosynthesis protein UbiB) | Membrane | DapA |
| W6QZD5 | PPSAL_2769 | BN5_2808 | S-formylglutathione hydrolase (EC 3.1.2.12) | Unknown | DapA |
| W6RDV0 | PPSAL_1460 | BN5_1465 | Putative cysteine desulfurase (EC 2.8.1.7) | Cytoplasmic | DapA |
| W6RE84 | PPSAL_1591 | BN5_1603 | Glutamate dehydrogenase | Unknown | DapA |
| W6QTI1 | PPSAL_0631 | BN5_0636 | Ferredoxin-NADP reductase (EC 1.18.1.2) | Cytoplasmic | DapA |
| W6QQV2 | PPSAL_0685 | BN5_0691 | Peptide chain release factor 3 (RF-3) | Cytoplasmic | DapA |
| W6R1B7 | PPSAL_3461 | BN5_3511 | SSU ribosomal protein S30P / sigma 54 modulation protein | Cytoplasmic | DapA |
| W6R1C0 | PPSAL_4252 | BN5_4316 | Bifunctional protein glk (EC 2.7.1.2) | Cytoplasmic | DapA |
| W6RGN1 | PPSAL_2465 | BN5_2495 | ATP-dependent DNA helicase RecQ (EC 3.6.1.-) | Cytoplasmic | DapA |
| W6QZ64 | PPSAL_3546 | BN5_3598 | GCN5-related N-acetyltransferase (EC 2.3.1.48) | Cytoplasmic | DapA |
| W6R2N2 | PPSAL_3892 | BN5_3951 | L-threonine aldolase (EC 4.1.2.48) | Unknown | DapA |
| W6QZ82 | PPSAL_3146 | BN5_3191 | Pyrimidine biosynthesis enzyme THI13 | Unknown | DapA |
| W6QZ32 | #N/A | BN5_3568 | Uncharacterized protein | Unknown | DapA |
| W6R6G6 | PPSAL_3374 | BN5_3424 | 4-hydroxy-3-methylbut-2-enyl diphosphate reductase (EC 1.17.7.4) | Cytoplasmic | DapA |
| W6QRA9 | PPSAL_0845 | BN5_0851 | FxsA | Membrane | DapA |
| W6QXR2 | PPSAL_0344 | BN5_0347 | Uncharacterized protein | Unknown | DapA |
| W6QZ98 | PPSAL_2729 | BN5_2768 | Alpha-methylacyl-CoA racemase (EC 5.1.99.4) | Cytoplasmic | DapA |
| W6QY17 | PPSAL_2768 | BN5_2807 | 2-C-methyl-D-erythritol 2,4-cyclodiphosphate synthase (MECDP-synthase) (MECPP-synthase) (MECPS) (EC 4.6.1.12) | Cytoplasmic | DapA |
| W6QS68 | PPSAL_0737 | BN5_0743 | CBS domain-containing protein | Membrane | DapA |
| W6R1Q7 | PPSAL_3609 | BN5_3661 | Uncharacterized protein | Unknown | DapA |
| W6RB67 | PPSAL_0487 | BN5_0490 | DNA topoisomerase 4 subunit B (EC 5.99.1.3) (Topoisomerase IV subunit B) | Cytoplasmic | DapA |
| W6QZN0 | PPSAL_3315 | BN5_3362 | Molybdopterin-synthase adenylyltransferase (EC 2.7.7.80) | Cytoplasmic | DapA |
| W6QYS5 | PPSAL_3033 | BN5_3076 | 7-cyano-7-deazaguanine synthase (EC 6.3.4.20) (7-cyano-7-carbaguanine synthase) (PreQ(0) synthase) (Queuosine biosynthesis protein QueC) | Unknown | DapA |
| W6R2K1 | PPSAL_4334 | BN5_4398 | COBW domain-containing protein 1 | Cytoplasmic | DapA |
| W6R0M3 | PPSAL_1370 | BN5_1375 | Putative ABC transporter ATP-binding protein MA_1747 (EC 3.6.3.-) | Cytoplasmic | DapA |
| W6R2P7 | PPSAL_2059 | BN5_2084 | Copper-exporting P-type ATPase A | Unknown | DapA |
| W6RCA9 | PPSAL_0914 | BN5_0920 | Ribosomal RNA large subunit methyltransferase E (EC 2.1.1.166) (23S rRNA Um2552 methyltransferase) (rRNA (uridine-2'-O-)-methyltransferase) | Cytoplasmic | DapA |
| W6R0V2 | PPSAL_3722 | BN5_3779 | Multifunctional CCA protein [Includes: CCA-adding enzyme (EC 2.7.7.72) (tRNA CCA-pyrophosphorylase) (tRNA nucleotidyltransferase) (tRNA-NT) (tRNA adenylyl-/cytidylyl-transferase) (CCA tRNA nucleotidyltransferase); 2',3'-cyclic phosphodiesterase (EC 3.1.4.-); Phosphatase (EC 3.1.3.-); 2'-nucleotidase] | Cytoplasmic | DapA |
| W6RJJ6 | PPSAL_3397 | BN5_3447 | ATP:cob(I)alamin adenosyltransferase (EC 2.5.1.17) | Cytoplasmic | DapA |
| W6R1B2 | PPSAL_4242 | BN5_4306 | Membrane associated protein slr1513 | Cytoplasmic | DapA |
| W6QYE7 | PPSAL_2444 | BN5_2474 | Two component transcriptional regulator | Cytoplasmic | DapA |
| W6QSN9 | PPSAL_0902 | BN5_0908 | DNA repair protein RecN (Recombination protein N) | Cytoplasmic | DapA |
| W6R745 | PPSAL_3582 | BN5_3634 | B12 family TonB-dependent receptor | OuterMembrane | DapA |
| W6R2L0 | PPSAL_2029 | BN5_2054 | LysR family transcriptional regulator | Membrane | DapA |
| W6QWP5 | PPSAL_2322 | BN5_2353 | Uncharacterized protein | Unknown | DapA |
| W6QUW3 | PPSAL_1167 | BN5_1171 | GTP cyclohydrolase 1 (EC 3.5.4.16) (GTP cyclohydrolase I) (GTP-CH-I) | Cytoplasmic | DapA |
| W6REL6 | PPSAL_1697 | BN5_1709 | UvrABC system protein B (Protein UvrB) (Excinuclease ABC subunit B) | Cytoplasmic | DapA |
| W6QPJ1 | PPSAL_0243 | BN5_0246 | 2-octaprenyl-6-methoxyphenyl hydroxylase (EC 1.14.13.-) | Membrane | DapA |

^1^Protein references correspond to UniProt accession numbers. Gene references correspond to GenBanK accession numbers HG916826 accession number^2^ (Wibberg *et al.*, 2014) and LK391695 accession number^3^ (Wibberg *et al.*, 2016). ^4^Subcellular location according with PSOTb v3.0.2. ^5^Fold change calculated as the ratio protein expression in the wild-type strain and the DapA1 ^─^ mutant (using the wild-type strain as reference).
