# Supplementary material for: Role of the Dihydrodipicolinate Synthase DapA1 on Iron Homeostasis During Cyanide Assimilation by the Alkaliphilic Bacterium Pseudomonas pseudoalcaligenes CECT5344
Source: Front Microbiol. 2020 Jan 23;11:28. doi: 10.3389/fmicb.2020.00028 (PMC6989483; doi:10.3389/fmicb.2020.00028)
Supplement: TABLE S2 — Quantitative proteomic analysis of the wild-type (reference) and DapA1– mutant strains of P. pseudoalcaligenes in ammonium. [file Table_2.DOCX]

**Table S2**. Quantitative proteomic analysis of the wild-type (reference) and DapA1ˉ mutant strains of *P. pseudoalcaligenes* in ammonium.

| **Protein ID^1^** | **Gene code^3^** | **Gene code^2^** | **Protein name** | **Location^4^** | **FC^5^** |
| --- | --- | --- | --- | --- | --- |
| W6RDQ8 | PPSAL_1415 | BN5_1420 | Uncharacterized protein yggL | Cytoplasmic | 31.39 |
| W6RDY8 | PPSAL_1495 | BN5_1500 | Carbon storage regulator homolog | Unknown | 7.25 |
| W6RAE8 | PPSAL_0178 | BN5_0180 | Ammonium transporter | Membrane | 5.79 |
| W6R8A0 | PPSAL_4034 | BN5_4096 | Phasin-like protein | Cytoplasmic | 4.21 |
| W6QXK4 | PPSAL_2130 | BN5_2157 | Outer membrane lipoprotein OprI | OuterMembrane | 3.91 |
| W6QWP3 | PPSAL_1811 | BN5_1832 | Uncharacterized protein | Unknown | 3.68 |
| W6RJI2 | PPSAL_3382 | BN5_3432 | 30S ribosomal protein S20 | Cytoplasmic | 3.45 |
| W6QSP8 | PPSAL_0401 | BN5_0404 | Phosphoribosyl-AMP cyclohydrolase (PRA-CH) (EC 3.5.4.19) | Cytoplasmic | 3.43 |
| W6QS66 | PPSAL_0207 | BN5_0209 | Alginate regulatory protein AlgP | Unknown | 3.13 |
| W6R3S3 | PPSAL_2442 | BN5_2472 | YciI like Protein | Unknown | 3.03 |
| W6R0R8 | PPSAL_4083 | BN5_4146 | Uncharacterized protein | Unknown | 2.82 |
| W6QZ30 | PPSAL_2654 | BN5_2689 | Indolepyruvate oxidoreductase subunit iorA (EC 1.2.7.8) | Cytoplasmic | 2.77 |
| W6QTP6 | PPSAL_0713 | BN5_0719 | Extracellular solute-binding protein | Periplasmic | 2.75 |
| W6RDZ3 | PPSAL_1500 | BN5_1509 | Arc | Cytoplasmic | 2.58 |
| W6QXC5 | PPSAL_0150 | BN5_0151 | Flavin monoamine oxidase-related protein (EC 1.4.3.4) | Unknown | 2.55 |
| W6QYF6 | PPSAL_2913 | BN5_2952 | Outer membrane porin | OuterMembrane | 2.50 |
| W6RBM7 | PPSAL_0622 | BN5_0627 | Cytochrome d ubiquinol oxidase, subunit I (EC 1.10.3.-) | Membrane | 2.45 |
| W6QXM0 | PPSAL_3016 | BN5_3058 | FMN-dependent NADH-azoreductase (EC 1.7.-.-) | Cytoplasmic | 2.41 |
| W6R9C0 | PPSAL_4388 | BN5_4452 | Alkyl hydroperoxide reductase AhpD (EC 1.11.1.15) | Unknown | 2.36 |
| W6RJ56 | PPSAL_3212 | BN5_3258 | Uncharacterized protein | Unknown | 2.21 |
| W6R218 | PPSAL_4145 | BN5_4208 | Deoxyuridine 5'-triphosphate nucleotidohydrolase (EC 3.6.1.23) | Cytoplasmic | 2.21 |
| W6QUZ0 | PPSAL_1197 | BN5_1201 | Probable D-serine dehydratase (EC 4.3.1.18) | Cytoplasmic | 2.19 |
| W6QXA7 | PPSAL_2901 | BN5_2940 | OmpA/MotB domain-containing protein | Membrane | 2.16 |
| W6R1B1 | PPSAL_3456 | BN5_3506 | 3-deoxy-D-manno-octulosonate 8-phosphate phosphatase (EC 3.1.3.45) | Cytoplasmic | 2.14 |
| W6QY01 | PPSAL_2748 | BN5_2787 | NAD(P)H dehydrogenase (quinone) (EC 1.6.5.2) | Unknown | 2.13 |
| W6R002 | PPSAL_2984 | BN5_3024 | Peptidase M14, carboxypeptidase A | Cytoplasmic | 2.12 |
| W6RJB1 | PPSAL_3292 | BN5_3339 | Uncharacterized protein | OuterMembrane | 2.10 |
| W6RGK3 | PPSAL_2435 | BN5_2465 | Pseudouridine synthase (EC 5.4.99.-) | Cytoplasmic | 2.09 |
| W6R342 | PPSAL_4036 | BN5_4098 | Phosphate acetyl/butaryl transferase (EC 2.3.1.8) | Cytoplasmic | 2.05 |
| W6R715 | PPSAL_3557 | BN5_3609 | Glyoxalase/bleomycin resistance protein/dioxygenase | Cytoplasmic | 2.02 |
| W6RGP7 | PPSAL_2480 | BN5_2510 | RND family efflux transporter MFP subunit | Membrane | 2.01 |
| W6QTJ2 | PPSAL_0648 | BN5_0654 | NADH dehydrogenase (EC 1.6.99.3) | Membrane | 2.00 |
| W6QZ36 | PPSAL_2659 | BN5_2694 | Cobalamin-independent methionine synthase (EC 2.1.1.14) | Cytoplasmic | 2.00 |
| W6QYG6 | PPSAL_3268 | BN5_3315 | Beta-lactamase domain-containing protein | Cytoplasmic | -2.01 |
| W6QTC3 | PPSAL_0611 | BN5_0616 | Alpha-2-macroglobulin domain-containing protein | Unknown | -2.02 |
| W6QZZ6 | PPSAL_3430 | BN5_3480 | Cytochrome b | Membrane | -2.03 |
| W6R8U0 | PPSAL_4188 | BN5_4252 | NAD(P) transhydrogenase subunit beta (EC 1.6.1.2) | Membrane | -2.05 |
| W6R541 | PPSAL_2902 | BN5_2941 | Beta-lactamase | Periplasmic | -2.09 |
| W6QQA5 | PPSAL_0066 | BN5_0066 | Alcohol dehydrogenase, zinc-containing (EC 1.1.1.1) | Cytoplasmic | -2.12 |
| W6QW89 | PPSAL_2154 | BN5_2181 | Succinate dehydrogenase, cytochrome b556 subunit (EC 1.3.99.1) | Membrane | -2.12 |
| W6QU17 | PPSAL_0848 | BN5_0854 | Polyprenyl synthetase (EC 2.5.1.-) | Cytoplasmic | -2.12 |
| W6R1B7 | PPSAL_3461 | BN5_3511 | SSU ribosomal protein S30P / sigma 54 modulation protein | Cytoplasmic | -2.12 |
| W6R8N2 | PPSAL_4139 | BN5_4202 | 50S ribosomal protein L33 | Cytoplasmic | -2.13 |
| W6QXI1 | PPSAL_2598 | BN5_2632 | Site-determining protein | Membrane | -2.14 |
| W6QSQ8 | PPSAL_0411 | BN5_0414 | Poly(3-hydroxyalkanoate) polymerase (EC 2.3.1.-) | Cytoplasmic | -2.19 |
| W6QYX6 | PPSAL_3453 | BN5_3503 | Uncharacterized protein | Membrane | -2.20 |
| W6RL89 | PPSAL_4042 | BN5_4104 | Acetyl-CoA acetyltransferase (EC 2.3.1.9) | Cytoplasmic | -2.21 |
| W6QSI1 | PPSAL_0331 | BN5_0334 | Methyl-accepting chemotaxis protein I | Membrane | -2.21 |
| W6R347 | PPSAL_2177 | BN5_2205 | Hydroxysteroid dehydrogenase-like protein 2 | Unknown | -2.23 |
| W6R782 | PPSAL_3617 | BN5_3669 | Catalase-peroxidase (CP) (EC 1.11.1.21) (Peroxidase/catalase) | Cytoplasmic | -2.23 |
| W6RHN5 | PPSAL_2740 | BN5_2779 | Nitrate reductase (EC 1.7.99.4) | Periplasmic | -2.24 |
| W6R9A9 | PPSAL_4378 | BN5_4442 | Uncharacterized protein | Unknown | -2.27 |
| W6RIL9 | PPSAL_3030 | BN5_3072 | Metallo-beta-lactamase family protein | Cytoplasmic | -2.28 |
| W6R0M7 | PPSAL_4048 | BN5_4110 | Uncharacterized lipoprotein ygdI | Unknown | -2.30 |
| W6RAM8 | PPSAL_0282 | BN5_0285 | Phosphoesterase (EC 3.1.3.4) | Membrane | -2.32 |
| W6R4S6 | PPSAL_2772 | BN5_2811 | Uncharacterized protein | Unknown | -2.37 |
| W6R8B1 | PPSAL_4044 | BN5_4106 | Secretion protein HlyD family protein | Membrane | -2.39 |
| W6QWB8 | PPSAL_2596 | BN5_2630 | Chemotaxis protein CheY | Cytoplasmic | -2.39 |
| W6QUH0 | PPSAL_1528 | BN5_1537 | Laminin subunit alphmethyl-accepting chemotaxis sensory transducer | Membrane | -2.39 |
| W6R013 | PPSAL_1135 | BN5_1139 | Superoxide dismutase (EC 1.15.1.1) | Periplasmic | -2.39 |
| W6QXI2 | PPSAL_0234 | BN5_0237 | Membrane-fusion protein | Membrane | -2.40 |
| W6QPJ9 | PPSAL_0253 | BN5_0256 | Cytochrome c551 peroxidase (EC 1.11.1.5) | Periplasmic | -2.45 |
| W6QVV2 | PPSAL_2020 | BN5_2045 | Methyl-accepting chemotaxis sensory transducer | Membrane | -2.45 |
| W6R176 | PPSAL_4202 | BN5_4266 | Putative virulence factor | OuterMembrane | -2.50 |
| W6QRM5 | PPSAL_0032 | BN5_0032 | Oxygen-dependent coproporphyrinogen-III oxidase (Coprogen oxidase) (EC 1.3.3.3) | Cytoplasmic | -2.56 |
| W6QU88 | PPSAL_1448 | BN5_1453 | 50S ribosomal protein L32 | Cytoplasmic | -2.58 |
| W6QSQ4 | PPSAL_0406 | BN5_0409 | Poly(Hydroxyalkanoate) granule-associated protein (Phasin) | Cytoplasmic | -2.60 |
| W6R020 | PPSAL_3004 | BN5_3044 | Peptidyl-prolyl cis-trans isomerase (EC 5.2.1.8) | Cytoplasmic | -2.64 |
| W6R3N9 | PPSAL_2412 | BN5_2442 | Cytochrome c oxidase, cbb3-type, subunit II (EC 1.9.3.1) | Unknown | -2.64 |
| W6RIW6 | PPSAL_3128 | BN5_3173 | Uncharacterized protein | Unknown | -2.65 |
| W6QYG8 | PPSAL_3273 | BN5_3320 | ATPase, P-type (Transporting), HAD superfamily, subfamily IC (EC 3.6.3.-) | Membrane | -2.65 |
| W6QZB3 | PPSAL_0901 | BN5_0907 | Ferric uptake regulation protein | Cytoplasmic | -2.69 |
| W6QYC5 | PPSAL_2419 | BN5_2449 | Cytochrome c oxidase accessory protein, cbb3-type, CcoG | Membrane | -2.73 |
| W6QRJ2 | PPSAL_0925 | BN5_0933 | 30S ribosomal protein S15 | Cytoplasmic | -2.76 |
| W6R0B7 | PPSAL_1250 | BN5_1255 | Uncharacterized protein | Unknown | -2.79 |
| W6QRG4 | PPSAL_0900 | BN5_0906 | Outer membrane protein assembly factor BamE | OuterMembrane | -2.79 |
| W6QZT0 | PPSAL_2914 | BN5_2953 | Outer membrane porin F | OuterMembrane | -2.82 |
| W6QYI9 | PPSAL_2484 | BN5_2514 | Acyl-CoA dehydrogenase (EC 1.3.99.-) | Membrane | -2.85 |
| W6QSI2 | PPSAL_0847 | BN5_0853 | 3-ketoacyl-(Acyl-carrier-protein) reductase (EC 1.1.1.100) | Cytoplasmic | -2.89 |
| W6QPR3 | PPSAL_0318 | BN5_0321 | UPF0312 protein BN5_0321 | Unknown | -2.94 |
| W6QRB3 | PPSAL_0850 | BN5_0856 | 50S ribosomal protein L27 | Cytoplasmic | -2.96 |
| W6QUA9 | PPSAL_0943 | BN5_0951 | RNA polymerase-binding transcription factor DksA | Cytoplasmic | -2.97 |
| W6R1X9 | PPSAL_1799 | BN5_1820 | Methyl-accepting chemotaxis protein mcpB | Membrane | -3.09 |
| W6RAQ0 | PPSAL_0307 | BN5_0310 | Response regulator receiver protein | Cytoplasmic | -3.12 |
| W6QQU8 | PPSAL_0680 | BN5_0686 | Uncharacterized protein | Membrane | -3.14 |
| W6QWH2 | PPSAL_1731 | BN5_1752 | DNA-binding protein HU | Cytoplasmic | -3.19 |
| W6RC58 | PPSAL_0844 | BN5_0850 | 10 kDa chaperonin (GroES protein) (Protein Cpn10) | Cytoplasmic | -3.23 |
| W6R1Q8 | PPSAL_4070 | BN5_4133 | Mannose-1-phosphate guanylyltransferase/mannose-6-phosphate isomerase (EC 2.7.7.22) | Cytoplasmic | -3.25 |
| W6QVV7 | PPSAL_2411 | BN5_2441 | Cytochrome c oxidase subunit 1 homolog, bacteroid (EC 1.9.3.1) | Membrane | -3.26 |
| W6QTW8 | PPSAL_1738 | BN5_1759 | Universal stress protein E homolog | Cytoplasmic | -3.30 |
| W6QWV3 | PPSAL_2741 | BN5_2780 | Periplasmic nitrate reductase, electron transfer subunit (Diheme cytochrome c NapB) | Periplasmic | -3.30 |
| H9N5D8 | PPSAL_1625 | BN5_1637 | FAD dependent oxidoreductase | Cytoplasmic | -3.43 |
| W6RHP8 | PPSAL_2755 | BN5_2794 | Uncharacterized protein | Periplasmic | -3.49 |
| W6R8V0 | PPSAL_4203 | BN5_4267 | Putative virulence effector protein | Unknown | -3.53 |
| W6R0Q0 | PPSAL_4068 | BN5_4131 | Putative capsule polysaccharide export protein | Unknown | -3.53 |
| W6R0E8 | PPSAL_1285 | BN5_1290 | Ribonucleoside-diphosphate reductase subunit beta (EC 1.17.4.1) | Cytoplasmic | -3.56 |
| W6REQ8 | PPSAL_1747 | BN5_1768 | Uncharacterized protein | Periplasmic | -3.59 |
| W6RLB1 | PPSAL_4067 | BN5_4130 | Putative tyrosine-protein kinase epsB (EC 2.7.10.-) | Membrane | -3.66 |
| W6RDK1 | PPSAL_1336 | BN5_1343 | Putative Ubiquinone biosynthesis hydroxylase (EC 1.-.-.-) | Cytoplasmic | -3.69 |
| W6R2R0 | PPSAL_2069 | BN5_2094 | Uncharacterized protein | Unknown | -3.79 |
| W6QYC8 | PPSAL_2424 | BN5_2454 | Coproporphyrinogen-III oxidase (EC 1.3.98.3) | Cytoplasmic | -3.89 |
| W6R3P5 | PPSAL_4200 | BN5_4264 | PpkA-related protein | Unknown | -3.92 |
| W6QVX1 | PPSAL_2426 | BN5_2456 | CRP/FNR family transcriptional regulator | Cytoplasmic | -3.96 |
| W6R0W1 | PPSAL_1452 | BN5_1457 | Acyl carrier protein (ACP) | Cytoplasmic | -4.00 |
| W6R686 | PPSAL_3284 | BN5_3331 | Arginine deiminase (ADI) (EC 3.5.3.6) | Cytoplasmic | -4.03 |
| W6R8X4 | PPSAL_4228 | BN5_4292 | Uncharacterized protein | Cytoplasmic | -4.12 |
| W6QXX0 | PPSAL_0399 | BN5_0402 | Sec-independent protein translocase protein TatA | Membrane | -4.16 |
| W6QYU3 | PPSAL_3048 | BN5_3091 | Ferritin, Dps family protein | Cytoplasmic | -4.26 |
| W6RFY0 | PPSAL_2161 | BN5_2188 | UPF0345 protein BN5_2188 | Unknown | -4.44 |
| W6QQK7 | PPSAL_0166 | BN5_0168 | Cytochrome c5-like protein | Unknown | -4.84 |
| W6R346 | PPSAL_4041 | BN5_4103 | Acetoacetyl-CoA reductase (EC 1.1.1.36) | Cytoplasmic | -4.93 |
| W6R3S5 | PPSAL_4230 | BN5_4294 | Uncharacterized protein | Unknown | -5.09 |
| W6QWD3 | PPSAL_1686 | BN5_1697 | Uncharacterized protein | Unknown | -5.13 |
| W6QZK4 | PPSAL_3285 | BN5_3332 | Ornithine carbamoyltransferase (OTCase) (EC 2.1.3.3) | Cytoplasmic | -5.28 |
| W6QYC1 | PPSAL_2414 | BN5_2444 | Cbb3-type cytochrome c oxidase subunit | Unknown | -6.06 |
| W6R1X7 | PPSAL_4135 | BN5_4198 | Laminin subunit gamma-1 | Unknown | -8.35 |
| W6RFI0 | PPSAL_2052 | BN5_2077 | Uncharacterized protein | Unknown | -8.62 |
| W6R3W1 | PPSAL_2477 | BN5_2507 | Uncharacterized protein | Unknown | -8.83 |
| W6QUJ8 | PPSAL_1554 | BN5_1565 | Putative orphan protein | OuterMembrane | -8.83 |
| W6R265 | PPSAL_1889 | BN5_1912 | Nitrilase (EC 3.5.5.1) | Cytoplasmic | -10.40 |
| W6QYL6 | PPSAL_3328 | BN5_3377 | Uncharacterized protein | Unknown | -12.75 |
| W6QUA1 | PPSAL_1888 | BN5_1911 | NADP-dependent malic enzyme (EC 1.1.1.40) | Cytoplasmic | -23.40 |
| W6RBZ4 | PPSAL_0764 | BN5_0770 | Uncharacterized protein | Cytoplasmic | -28.49 |
| W6RK11 | PPSAL_3540 | BN5_3592 | Acyl-CoA dehydrogenase family protein | Cytoplasmic | -43.51 |
| W6REH9 | PPSAL_1646 | BN5_1658 | Helix-hairpin-helix repeat-containing competence protein ComEA | Membrane | -95.38 |
| W6R0A3 | PPSAL_3538 | BN5_3590 | Fatty-acyl-CoA synthase (EC 2.3.1.86) | Cytoplasmic | -145.11 |
| W6R0D1 | PPSAL_3113 | BN5_3157 | Histidine kinase (EC 2.7.13.3) | Membrane | WT |
| W6R0R1 | PPSAL_3672 | BN5_3729 | Iron-sulfur cluster insertion protein ErpA | Cytoplasmic | WT |
| W6QPB8 | PPSAL_0164 | BN5_0166 | Alanine racemase (EC 5.1.1.1) | Cytoplasmic | WT |
| W6QW69 | PPSAL_2546 | BN5_2580 | Twitching motility protein | Cytoplasmic | WT |
| W6RI49 | PPSAL_2875 | BN5_2914 | Putative polar amino acid transport system substrate-binding proetin | Unknown | WT |
| W6QRS4 | PPSAL_0590 | BN5_0595 | Allophanate hydrolase (EC 3.5.1.4) | Cytoplasmic | WT |
| W6R1J2 | PPSAL_3544 | BN5_3596 | Flavin prenyltransferase UbiX (EC 2.5.1.129) | Cytoplasmic | WT |
| W6R1D6 | PPSAL_3936 | BN5_3995 | Abhydrolase domain-containing protein 3 | Unknown | WT |
| W6R5M0 | PPSAL_3062 | BN5_3105 | Uncharacterized protein | Unknown | WT |
| W6R6F7 | PPSAL_3364 | BN5_3414 | Sensor protein PilS (EC 2.7.13.3) | Membrane | WT |
| W6QSI3 | PPSAL_1284 | BN5_1289 | Uncharacterized protein | Cytoplasmic | DapA |
| W6QS74 | PPSAL_0742 | BN5_0748 | Uncharacterized protein | Cytoplasmic | DapA |
| W6R8V9 | PPSAL_4213 | BN5_4277 | Uncharacterized protein | Cytoplasmic | DapA |
| W6R414 | PPSAL_2522 | BN5_2556 | 50S ribosomal protein L3 glutamine methyltransferase (L3 MTase) (EC 2.1.1.298) (N5-glutamine methyltransferase PrmB) | Cytoplasmic | DapA |
| W6R3Z8 | PPSAL_4300 | BN5_4364 | Sialic acid TRAP transporter permease protein siaT | Membrane | DapA |
| W6QXG1 | PPSAL_2951 | BN5_2990 | Branched-chain amino acid transport system ATP-binding protein (EC 3.6.3.27) | Membrane | DapA |
| W6RGM6 | PPSAL_2460 | BN5_2490 | Putative selenoprotein | Cytoplasmic | DapA |
| W6RCR6 | PPSAL_1073 | BN5_1077 | Putative acyl-CoA thioester hydrolase TC_0822 (EC 3.1.2.-) | Unknown | DapA |
| W6RD43 | PPSAL_1153 | BN5_1157 | Uncharacterized protein | Unknown | DapA |
| W6RBQ5 | PPSAL_0642 | BN5_0647 | Diguanylate cyclase/phosphodiesterase with PAS/PAC sensor(S) | Membrane | DapA |
| W6RDF9 | PPSAL_1288 | BN5_1293 | Putative transcriptional regulator ycf27 | Cytoplasmic | DapA |
| W6QTK4 | PPSAL_0663 | BN5_0669 | Uncharacterized protein | Unknown | DapA |
| W6R263 | PPSAL_3733 | BN5_3791 | Uncharacterized protein | Unknown | DapA |
| W6R2X4 | PPSAL_3967 | BN5_4026 | Uncharacterized protein HI0217 | Unknown | DapA |
| W6RIR6 | PPSAL_3074 | BN5_3118 | VacJ family lipoprotein | OuterMembrane | DapA |
| W6QXM2 | PPSAL_2638 | BN5_2672 | Flagellar basal-body rod protein FlgG (Distal rod protein) | Extracellular | DapA |
| W6QUG0 | PPSAL_1518 | BN5_1527 | BolA-like protein 1 | Unknown | DapA |
| W6R1N2 | PPSAL_3584 | BN5_3636 | Uncharacterized protein | Membrane | DapA |
| W6RBA0 | PPSAL_0517 | BN5_0520 | tRNA dimethylallyltransferase (EC 2.5.1.75) | Cytoplasmic | DapA |
| W6RD68 | PPSAL_1183 | BN5_1187 | Response regulator arlR | Cytoplasmic | DapA |
| W6RC07 | PPSAL_0779 | BN5_0785 | Uncharacterized protein | Unknown | DapA |
| W6QX98 | PPSAL_0126 | BN5_0125 | GlcG protein | Unknown | DapA |
| W6RDT1 | PPSAL_1440 | BN5_1445 | Low molecular weight phosphotyrosine protein phosphatase (EC 3.1.3.48) | Cytoplasmic | DapA |
| W6QS06 | PPSAL_0662 | BN5_0668 | Uncharacterized protein | Unknown | DapA |
| W6QYQ8 | PPSAL_3018 | BN5_3060 | LysR family transcriptional regulator | Cytoplasmic | DapA |
| W6QVQ6 | PPSAL_1464 | BN5_1469 | Lipoprotein, putative | Unknown | DapA |
| W6R3M9 | PPSAL_2402 | BN5_2432 | TetR family transcriptional regulator | Unknown | DapA |
| W6R0B5 | PPSAL_3553 | BN5_3605 | Aminocarboxymuconate-semialdehyde decarboxylase (EC 4.1.1.45) | Cytoplasmic | DapA |
| W6QW58 | PPSAL_1630 | BN5_1642 | YecA family protein | Cytoplasmic | DapA |
| W6RBI1 | PPSAL_0592 | BN5_0597 | Integral membrane sensor hybrid histidine kinase (EC 2.7.13.3) | Membrane | DapA |
| W6R016 | PPSAL_3829 | BN5_3888 | Diguanylate cyclase | Membrane | DapA |
| W6RJC3 | PPSAL_3307 | BN5_3354 | Short-chain dehydrogenase/reductase SDR (EC 1.1.1.100) | Unknown | DapA |
| W6R9W6 | PPSAL_0043 | BN5_0043 | Glucose 1-dehydrogenase (EC 1.1.1.47) | Cytoplasmic | DapA |
| W6QV85 | PPSAL_2181 | BN5_2209 | Putative membrane protein | Membrane | DapA |
| W6QYY2 | PPSAL_2634 | BN5_2668 | Flagellar hook-associated protein 1 | Extracellular | DapA |
| W6QY70 | PPSAL_0494 | BN5_0497 | 3-deoxy-D-manno-octulosonic-acid transferase (EC 2.-.-.-) | Unknown | DapA |
| W6QQY1 | PPSAL_0715 | BN5_0721 | Sensor histidine kinase | Membrane | DapA |
| W6QSL5 | PPSAL_0366 | BN5_0369 | ATP-dependent RNA helicase | Unknown | DapA |
| W6RG31 | PPSAL_2220 | BN5_2248 | Uncharacterized protein | Cytoplasmic | DapA |
| W6RA04 | PPSAL_0078 | BN5_0078 | LysR family transcriptional regulator | Cytoplasmic | DapA |
| W6QRT0 | PPSAL_0092 | BN5_0092 | Cytochrome c oxidase subunit 2 (EC 1.9.3.1) | Membrane | DapA |
| W6QSU8 | PPSAL_1406 | BN5_1411 | Putative competence-damage inducible protein | Unknown | DapA |
| W6R051 | PPSAL_3034 | BN5_3077 | 7-carboxy-7-deazaguanine synthase (EC 4.3.99.3) | Cytoplasmic | DapA |
| W6R0B9 | PPSAL_3944 | BN5_4003 | Uncharacterized protein yjbB | Membrane | DapA |
| W6R6E9 | PPSAL_3354 | BN5_3404 | Sulfate transporter CysZ | Membrane | DapA |
| W6QZS2 | PPSAL_1070 | BN5_1074 | Ribosome maturation factor RimM | Cytoplasmic | DapA |
| W6QYT6 | PPSAL_3043 | BN5_3086 | Holliday junction ATP-dependent DNA helicase RuvA (EC 3.6.4.12) | Cytoplasmic | DapA |
| W6QZI2 | PPSAL_3255 | BN5_3302 | Uncharacterized protein | Unknown | DapA |
| W6QZ59 | PPSAL_0846 | BN5_0852 | Pyridoxamine 5'-phosphate oxidase-related, FMN-binding (EC 1.4.3.5) | Cytoplasmic | DapA |
| W6QSS8 | PPSAL_0942 | BN5_0950 | Glutamyl-Q tRNA(Asp) synthetase (Glu-Q-RSs) (EC 6.1.1.-) | Cytoplasmic | DapA |
| W6RGJ0 | PPSAL_2420 | BN5_2450 | Cytochrome c oxidase accessory protein CcoH | Membrane | DapA |
| W6R0C8 | PPSAL_1265 | BN5_1270 | Putative peptidase (EC 3.4.-.-) | Cytoplasmic | DapA |
| W6QU30 | PPSAL_0863 | BN5_0869 | Methyl-accepting chemotaxis sensory transducer | Membrane | DapA |
| W6RJ65 | PPSAL_3227 | BN5_3273 | Uncharacterized protein | Unknown | DapA |
| W6QZS5 | PPSAL_3715 | BN5_3772 | Ribosomal RNA small subunit methyltransferase A (EC 2.1.1.182) | Cytoplasmic | DapA |
| W6QXW7 | PPSAL_2713 | BN5_2749 | 2,4-dienoyl-coa reductase FADH1, putative (EC 1.3.1.34) | Cytoplasmic | DapA |
| W6RHH5 | PPSAL_2695 | BN5_2731 | Uncharacterized protein | Unknown | DapA |
| W6QSS0 | PPSAL_0421 | BN5_0424 | Peptidase (EC 3.4.24.-) | Cytoplasmic | DapA |
| W6R2Q1 | PPSAL_4379 | BN5_4443 | Nitrate reductase (EC 1.7.1.1) | Unknown | DapA |
| W6QV90 | PPSAL_2186 | BN5_2214 | Putative Orn/Lys/Arg decarboxylase (EC 4.1.1.19) | Cytoplasmic | DapA |
| W6QRA9 | PPSAL_0845 | BN5_0851 | FxsA | Membrane | DapA |
| W6R9E0 | PPSAL_4408 | BN5_4472 | Uncharacterized protein | Unknown | DapA |
| W6QXR2 | PPSAL_0344 | BN5_0347 | Uncharacterized protein | Unknown | DapA |
| W6QV53 | PPSAL_1262 | BN5_1267 | Uncharacterized protein | Periplasmic | DapA |
| W6QRS6 | PPSAL_0087 | BN5_0087 | L-asparaginase I (EC 3.5.1.1) | Cytoplasmic | DapA |
| W6R4J6 | PPSAL_2662 | BN5_2697 | Solvent efflux pump periplasmic linker srpA | Membrane | DapA |
| W6RCL0 | PPSAL_1019 | BN5_1022 | TRAP-type uncharacterized transport system periplasmic component-like protein | Unknown | DapA |
| W6QXN1 | PPSAL_0304 | BN5_0307 | TonB domain protein | Unknown | DapA |
| W6R146 | PPSAL_4163 | BN5_4226 | ATP-dependent DNA helicase RecG (EC 3.6.4.12) | Cytoplasmic | DapA |
| W6QYJ6 | PPSAL_3308 | BN5_3355 | Amine oxidase (EC 1.4.3.2) | Unknown | DapA |
| W6RJJ6 | PPSAL_3397 | BN5_3447 | ATP:cob(I)alamin adenosyltransferase (EC 2.5.1.17) | Cytoplasmic | DapA |
| W6R1B2 | PPSAL_4242 | BN5_4306 | Membrane associated protein slr1513 | Cytoplasmic | DapA |
| W6QU22 | PPSAL_0853 | BN5_0859 | CreA family protein | Membrane | DapA |
| W6R0X5 | PPSAL_3301 | BN5_3348 | FAD dependent oxidoreductase | Unknown | DapA |
| W6QZ88 | PPSAL_3151 | BN5_3196 | Cytokinin riboside 5'-monophosphate phosphoribohydrolase (EC 3.2.2.n1) | Unknown | DapA |
| W6REL6 | PPSAL_1697 | BN5_1709 | UvrABC system protein B (Protein UvrB) | Cytoplasmic | DapA |
| W6RG11 | PPSAL_2200 | BN5_2228 | NasT | Cytoplasmic | DapA |
| W6R0R6 | PPSAL_1412 | BN5_1417 | Ribosome modulation factor (RMF) | Unknown | DapA |
| W6RL93 | PPSAL_4047 | BN5_4109 | Alkaline protease secretion protein aprF | OuterMembrane | DapA |
| W6QR06 | PPSAL_0740 | BN5_0746 | tRNA-2-methylthio-N(6)-dimethylallyladenosine synthase (EC 2.8.4.3) | Cytoplasmic | DapA |
| W6QWG0 | PPSAL_1716 | BN5_1737 | Uncharacterized protein | Unknown | DapA |
| W6R0X0 | PPSAL_3296 | BN5_3343 | Uncharacterized protein | Unknown | DapA |
| W6R0M2 | PPSAL_4043 | BN5_4105 | Poly(R)-hydroxyalkanoic acid synthase, class I (EC 2.3.1.-) | Cytoplasmic | DapA |
| W6QZY9 | PPSAL_2969 | BN5_3008 | 3-hydroxyacyl-CoA-acyl carrier protein transferase (EC 2.4.1.-) | Cytoplasmic | DapA |
| W6QVC1 | PPSAL_1330 | BN5_1337 | Putative signal transduction protein | Unknown | DapA |
| W6R8D9 | PPSAL_4069 | BN5_4132 | Glycosyl transferase, group 2 family protein (EC 2.4.1.-) | Cytoplasmic | DapA |
| W6QXG8 | PPSAL_0214 | BN5_0217 | UPF0178 protein BN5_0217 | Cytoplasmic | DapA |
| W6RH97 | PPSAL_2640 | BN5_2674 | Flagellar hook protein FlgE | Extracellular | DapA |
| W6RHX3 | PPSAL_2815 | BN5_2854 | Uncharacterized protein | Unknown | DapA |
| W6QWS4 | PPSAL_1836 | BN5_1857 | Uncharacterized protein | Cytoplasmic | DapA |
| W6R2T8 | PPSAL_4409 | BN5_4473 | Mercuric resistance operon regulatory protein | Cytoplasmic | DapA |
| W6RC50 | PPSAL_0834 | BN5_0840 | TetR family transcriptional regulator | Cytoplasmic | DapA |
| W6QVC0 | PPSAL_1820 | BN5_1841 | NADH dehydrogenase I chain M (EC 1.6.5.3) | Membrane | DapA |
| W6QV05 | PPSAL_1212 | BN5_1216 | Protein psiE homolog | Membrane | DapA |
| W6QZH9 | PPSAL_2814 | BN5_2853 | Uncharacterized protein | Unknown | DapA |
| W6R287 | PPSAL_4214 | BN5_4278 | Uncharacterized protein | Membrane | DapA |
| W6QXT3 | PPSAL_0364 | BN5_0367 | Extracellular solute-binding protein | Unknown | DapA |
| W6RKH6 | PPSAL_3679 | BN5_3736 | Inner membrane protein YhaI | Membrane | DapA |
| W6QQN7 | PPSAL_0206 | BN5_0208 | Peptidyl-prolyl cis-trans isomerase (EC 5.2.1.8) | Unknown | DapA |
| W6RLA6 | PPSAL_4062 | BN5_4125 | Membrane protein, putative | Membrane | DapA |
| W6QXX9 | PPSAL_0409 | BN5_0412 | Polyhydroxyalkanoate synthase, class II (EC 2.3.1.-) | Cytoplasmic | DapA |
| W6R0Y8 | PPSAL_3316 | BN5_3363 | Release factor glutamine methyltransferase (EC 2.1.1.297) | Cytoplasmic | DapA |
| W6R3M2 | PPSAL_4171 | BN5_4234 | Signal transduction histidine kinase (EC 2.7.13.3) | Membrane | DapA |
| W6R863 | #N/A | BN5_4059 | Myocilin | Unknown | DapA |
| W6QY76 | PPSAL_2364 | BN5_2394 | Thioesterase superfamily protein | Cytoplasmic | DapA |
| W6QZ04 | PPSAL_3112 | BN5_3156 | Uncharacterized protein | Unknown | DapA |
| W6R129 | PPSAL_3371 | BN5_3421 | Type IV pilus assembly protein PilX | Unknown | DapA |
| W6R3R6 | PPSAL_4220 | BN5_4284 | Hydrolase, haloacid dehalogenase-like family (EC 3.1.3.18) | Cytoplasmic | DapA |
| W6QRP7 | PPSAL_0052 | BN5_0052 | Uncharacterized protein | Membrane | DapA |
| W6QXW2 | PPSAL_3105 | BN5_3149 | UPF0114 protein BN5_3149 | Membrane | DapA |
| W6RDG4 | PPSAL_1293 | BN5_1298 | Solute carrier family 10 member 6 | Membrane | DapA |
| W6QVC5 | PPSAL_1825 | BN5_1846 | NADH-quinone oxidoreductase subunit H (EC 1.6.5.11) | Membrane | DapA |
| W6QZJ4 | PPSAL_2829 | BN5_2868 | Uncharacterized protein | Membrane | DapA |
| W6R490 | PPSAL_2587 | BN5_2621 | Uncharacterized protein | Unknown | DapA |
| W6R194 | PPSAL_3891 | BN5_3950 | Uncharacterized protein | Periplasmic | DapA |
| W6QPC7 | PPSAL_0174 | BN5_0176 | Mg chelatase, subunit ChlI | Cytoplasmic | DapA |
| W6R110 | PPSAL_1497 | BN5_1506 | Uncharacterized protein | Unknown | DapA |
| W6QV54 | PPSAL_1750 | BN5_1771 | Putative membrane protein | Membrane | DapA |
| W6QXB2 | PPSAL_2906 | BN5_2945 | Cation antiporter | Membrane | DapA |
| W6QRG1 | PPSAL_0895 | BN5_0901 | GntR family transcriptional regulator | Cytoplasmic | DapA |
| W6R2Z5 | PPSAL_3987 | BN5_4046 | Uncharacterized protein ybbK | Cytoplasmic | DapA |
| W6QU43 | PPSAL_1396 | BN5_1401 | Uncharacterized protein HI0173 | Unknown | DapA |
| W6QYX4 | PPSAL_3077 | BN5_3121 | Lipid A biosynthesis lauroyltransferase (EC 2.3.1.241) | Membrane | DapA |
| W6R3E6 | PPSAL_2306 | BN5_2337 | Coenzyme PQQ synthesis protein E | Cytoplasmic | DapA |
| W6QWN7 | PPSAL_2661 | BN5_2696 | HTH-type transcriptional regulator betI | Cytoplasmic | DapA |
| W6R4P8 | PPSAL_2742 | BN5_2781 | Cytochrome c-type protein | Membrane | DapA |
| W6RCP9 | PPSAL_1058 | BN5_1062 | UPF0135 protein | Unknown | DapA |
| W6RH10 | PPSAL_2605 | BN5_2639 | Flagellar protein | Membrane | DapA |
| W6QUU4 | PPSAL_1142 | BN5_1146 | Uncharacterized protein | Membrane | DapA |
| W6R0H7 | PPSAL_1320 | BN5_1325 | Uncharacterized protein | Unknown | DapA |
| W6RH20 | PPSAL_2615 | BN5_2649 | Flagellar assembly protein H | Cytoplasmic | DapA |
| W6QYL3 | PPSAL_0619 | BN5_0624 | Lipoprotein, putative | Periplasmic | DapA |
| W6RJ69 | PPSAL_3237 | BN5_3284 | Permease YjgP/YjgQ family protein | Membrane | DapA |
| W6R4A2 | PPSAL_2597 | BN5_2631 | RNA polymerase sigma factor FliA (Sigma-28) | Cytoplasmic | DapA |
| W6R217 | PPSAL_1844 | BN5_1865 | Cold shock-like protein cspG | Cytoplasmic | DapA |
| W6QXL9 | PPSAL_2633 | BN5_2667 | Flagellin B | Extracellular | DapA |
| W6QXZ2 | PPSAL_3129 | BN5_3174 | Uncharacterized protein | Unknown | DapA |
| W6R9F0 | PPSAL_4418 | BN5_4482 | Transposition protein TniB | Cytoplasmic | DapA |
| W6R1G3 | PPSAL_3511 | BN5_3561 | Transcriptional regulator | Unknown | DapA |
| W6QWE5 | PPSAL_2616 | BN5_2650 | Flagellar motor switch protein FliG | Cytoplasmic | DapA |
| W6QXC2 | PPSAL_2543 | BN5_2577 | Membrane protein, putative | Membrane | DapA |
| W6QWB0 | PPSAL_2178 | BN5_2206 | Acyl-CoA dehydrogenase family member 11 | Cytoplasmic | DapA |
| W6QSP3 | PPSAL_0907 | BN5_0913 | Uncharacterized protein | Cytoplasmic | DapA |
| W6R0P3 | PPSAL_4063 | BN5_4126 | Glycosyl transferase, group 1 family protein (EC 2.4.1.-) | Cytoplasmic | DapA |
| W6QTY0 | PPSAL_1753 | BN5_1774 | Methyl-accepting chemotaxis protein tlpB | Membrane | DapA |
| W6QXF6 | PPSAL_2946 | BN5_2985 | Uncharacterized protein | Cytoplasmic | DapA |
| W6QS97 | PPSAL_1204 | BN5_1208 | Lipoprotein, putative | Unknown | DapA |
| W6QS95 | PPSAL_0762 | BN5_0768 | CRISPR-associated Cse1 family protein | Unknown | DapA |
| W6QS82 | PPSAL_1189 | BN5_1193 | Uncharacterized protein | Cytoplasmic | DapA |
| W6QTT9 | PPSAL_0763 | BN5_0769 | CRISPR-associated protein, Cse2 family | Unknown | DapA |
| W6QR92 | PPSAL_0415 | BN5_0418 | Uncharacterized protein | Cytoplasmic | DapA |
| W6R0Y2 | PPSAL_4133 | BN5_4196 | Uncharacterized protein | Cytoplasmic | DapA |
| W6QR33 | PPSAL_0765 | BN5_0771 | CRISPR-associated Cas5e family protein | Unknown | DapA |
| W6QWG1 | PPSAL_2626 | BN5_2660 | Flagellar hook-associated protein 2 | Extracellular | DapA |
| W6RJJ1 | PPSAL_3392 | BN5_3442 | Mechanosensitive ion channel | Membrane | DapA |
| W6QXL0 | PPSAL_2135 | BN5_2162 | Putative ABC transporter permease ybbP | Membrane | DapA |
| W6R1I7 | PPSAL_3539 | BN5_3591 | Uncharacterized protein | Membrane | DapA |
| W6QQV6 | PPSAL_0285 | BN5_0288 | DNA helicase (EC 3.6.1.-) | Cytoplasmic | DapA |
| W6RGB7 | PPSAL_2324 | BN5_2355 | Beta-propeller repeat protein | Unknown | DapA |
| W6RBV6 | PPSAL_0714 | BN5_0720 | Uncharacterized protein | Unknown | DapA |
| W6R0T5 | PPSAL_3251 | BN5_3298 | Uncharacterized protein | Cytoplasmic | DapA |
| W6QSC5 | PPSAL_1239 | BN5_1243 | Nuclear receptor binding factor related protein (EC 1.3.1.38) | Cytoplasmic | DapA |
| W6R835 | PPSAL_3965 | BN5_4024 | Uncharacterized membrane protein NMB1645 | Membrane | DapA |
| W6R2I8 | PPSAL_2009 | BN5_2034 | DNA-binding transcriptional activator OsmE | Unknown | DapA |
| W6RKZ1 | PPSAL_3948 | BN5_4007 | Uncharacterized protein | Cytoplasmic | DapA |
| W6QY36 | PPSAL_2788 | BN5_2827 | Zinc metalloprotease (EC 3.4.24.-) | Membrane | DapA |
| W6QXF1 | PPSAL_2941 | BN5_2980 | OmpA/MotB domain-containing protein | OuterMembrane | DapA |
| W6QZ57 | PPSAL_3541 | BN5_3593 | Beta-lactamase domain-containing protein | Cytoplasmic | DapA |
| W6QVJ4 | PPSAL_1404 | BN5_1409 | Ferredoxin-2 | Cytoplasmic | DapA |
| W6RGZ5 | PPSAL_2590 | BN5_2624 | ParA family protein | Unknown | DapA |
| W6QWH4 | PPSAL_2636 | BN5_2670 | Flagellar P-ring protein (Basal body P-ring protein) | Periplasmic | DapA |
| W6QY98 | PPSAL_2853 | BN5_2892 | Carboxy-S-adenosyl-L-methionine synthase (EC 2.1.3.-) | Cytoplasmic | DapA |
| W6QSG9 | PPSAL_1269 | BN5_1274 | Ecotin | Periplasmic | DapA |
| W6QPI0 | PPSAL_0233 | BN5_0236 | Putative ABC transporter ATP-binding protein yhiH | Membrane | DapA |
| W6R372 | PPSAL_4066 | BN5_4129 | Glycosyl transferase, group 1 family protein | Cytoplasmic | DapA |
| W6QUH1 | PPSAL_1028 | BN5_1031 | Adenine-specific DNA-methyltransferase (EC 2.1.1.72) | Cytoplasmic | DapA |
| W6R0U0 | PPSAL_1432 | BN5_1437 | Lipoprotein-releasing system transmembrane protein lolC | Membrane | DapA |
| W6R3U8 | PPSAL_4250 | BN5_4314 | Putative oxidoreductase subunit (EC 1.3.99.16) | Cytoplasmic | DapA |
| W6RI82 | PPSAL_2915 | BN5_2954 | Uncharacterized protein | Unknown | DapA |
| W6QXA8 | PPSAL_2031 | BN5_2056 | Putative CheW protein (EC 2.7.13.3) | Cytoplasmic | DapA |
| W6R179 | PPSAL_3421 | BN5_3471 | Transcriptional regulator MraZ | Cytoplasmic | DapA |
| W6QWY1 | PPSAL_1886 | BN5_1909 | Methylenetetrahydrofolate reductase (EC 1.5.1.20) | Cytoplasmic | DapA |
| W6QWV7 | PPSAL_0025 | BN5_0025 | Ribosomal RNA small subunit methyltransferase B (EC 2.1.1.-) | Cytoplasmic | DapA |
| W6QZU3 | PPSAL_1090 | BN5_1094 | Lipoprotein, putative | Unknown | DapA |
| W6QXY2 | PPSAL_2249 | BN5_2277 | Methyl-accepting chemotaxis sensory transducer | Membrane | DapA |
| W6QRI7 | PPSAL_0920 | BN5_0928 | Ribosome maturation factor RimP | Cytoplasmic | DapA |
| W6R360 | PPSAL_2197 | BN5_2225 | Peptide ABC transporter,putative ATP-binding protein (EC 3.6.3.25) | Membrane | DapA |
| W6RB89 | PPSAL_0507 | BN5_0510 | Chemotaxis protein | Membrane | DapA |
| W6RDV6 | PPSAL_1465 | BN5_1470 | Uncharacterized protein | Cytoplasmic | DapA |
| W6QU47 | PPSAL_1828 | BN5_1849 | NADH dehydrogenase I chain E (EC 1.6.5.3) | Cytoplasmic | DapA |
| W6RLS9 | PPSAL_4231 | BN5_4295 | Uncharacterized protein | Unknown | DapA |
| W6QR86 | PPSAL_0410 | BN5_0413 | Poly(3-hydroxyalkanoate) depolymerase (EC 3.1.1.-) | Unknown | DapA |
| W6QR22 | PPSAL_0755 | BN5_0761 | Acyl-CoA dehydrogenase domain-containing protein | Cytoplasmic | DapA |
| W6RI74 | PPSAL_2905 | BN5_2944 | Putative multicomponent Na+:H+ antiporter subunit D (EC 1.6.99.5) | Membrane | DapA |
| W6QT87 | PPSAL_1091 | BN5_1095 | Uncharacterized protein | Cytoplasmic | DapA |
| W6QX72 | PPSAL_0120 | BN5_0120 | Response regulator receiver protein | Cytoplasmic | DapA |
| W6QUI6 | PPSAL_1544 | BN5_1555 | Histidine kinase (EC 2.7.13.3) | Membrane | DapA |
| W6R4F2 | PPSAL_4445 | BN5_4509 | tRNA modification GTPase MnmE (EC 3.6.-.-) | Cytoplasmic | DapA |
| W6R7H6 | PPSAL_3721 | BN5_3778 | SpoVR family protein | Cytoplasmic | DapA |
| W6QSZ2 | PPSAL_0491 | BN5_0494 | ADP-ribose pyrophosphatase (EC 3.6.1.13) | Cytoplasmic | DapA |
| W6RBU5 | PPSAL_0699 | BN5_0705 | TetR family transcriptional regulator | Cytoplasmic | DapA |
| W6QR81 | PPSAL_0405 | BN5_0408 | Putative polyhydroxyalkanoic acid system protein | Cytoplasmic | DapA |
| W6QUD4 | PPSAL_0983 | BN5_0986 | Putative Zinc metalloprotease (EC 3.4.24.-) | Cytoplasmic | DapA |
| W6R1Z9 | PPSAL_1824 | BN5_1845 | NADH-quinone oxidoreductase subunit I (EC 1.6.5.11) | Unknown | DapA |
| W6R1F7 | PPSAL_4292 | BN5_4356 | Antitoxin | Unknown | DapA |
| W6R739 | PPSAL_3577 | BN5_3629 | Exodeoxyribonuclease 7 small subunit (EC 3.1.11.6) | Cytoplasmic | DapA |
| W6R1J4 | PPSAL_4000 | BN5_4060 | Glycyl-glycine endopeptidase ALE-1 (EC 3.4.24.75) | OuterMembrane | DapA |
| W6R2G2 | PPSAL_4299 | BN5_4363 | Uncharacterized protein | Unknown | DapA |
| W6QZE1 | PPSAL_3611 | BN5_3663 | NAD-dependent epimerase/dehydratase (EC 5.1.3.2) | Cytoplasmic | DapA |
| W6R090 | PPSAL_3914 | BN5_3973 | Homoserine O-acetyltransferase (EC 2.3.1.31) | Cytoplasmic | DapA |
| W6QVF9 | PPSAL_2256 | BN5_2284 | 6-carboxy-5,6,7,8-tetrahydropterin synthase (EC 4.-.-.-) | Cytoplasmic | DapA |
| W6QXQ4 | PPSAL_3056 | BN5_3099 | Uncharacterized protein | Cytoplasmic | DapA |
| W6QTI1 | PPSAL_0631 | BN5_0636 | Ferredoxin-NADP reductase (EC 1.18.1.2) | Cytoplasmic | DapA |
| W6QT22 | PPSAL_1036 | BN5_1040 | Glutathione S-transferase (EC 2.5.1.18) | Cytoplasmic | DapA |
| W6QTB0 | PPSAL_0601 | BN5_0606 | 3-dehydroquinate dehydratase (EC 4.2.1.10) | Cytoplasmic | DapA |
| W6R0F7 | PPSAL_1295 | BN5_1300 | Glucose-6-phosphate 1-dehydrogenase (G6PD) (EC 1.1.1.49) | Cytoplasmic | DapA |
| W6R092 | PPSAL_3073 | BN5_3117 | Putative serine protein kinase | Unknown | DapA |
| W6QVV8 | PPSAL_1514 | BN5_1523 | FMN-dependent NADH-azoreductase (EC 1.7.-.-) | Cytoplasmic | DapA |
| W6RB28 | PPSAL_0447 | BN5_0450 | Citrate transporter | Membrane | DapA |
| W6QQR6 | PPSAL_0240 | BN5_0243 | Uncharacterized protein | Unknown | DapA |
| W6RHJ2 | PPSAL_2705 | BN5_2741 | Uncharacterized protein | Cytoplasmic | DapA |
| W6R1K1 | PPSAL_4347 | BN5_4411 | Heavy metal sensor histidine kinase (EC 2.7.13.3) | Membrane | DapA |
| W6RGN1 | PPSAL_2465 | BN5_2495 | ATP-dependent DNA helicase RecQ (EC 3.6.1.-) | Cytoplasmic | DapA |
| W6R4R5 | PPSAL_2762 | BN5_2801 | RNA polymerase sigma factor RpoS (Sigma S) (Sigma-38) | Cytoplasmic | DapA |
| W6QY39 | PPSAL_2318 | BN5_2349 | Pentapeptide repeat-containing protein | Extracellular | DapA |
| W6R0D9 | PPSAL_3964 | BN5_4023 | Glycerophosphoryl diester phosphodiesterase (EC 3.1.4.46) | Periplasmic | DapA |
| W6QW66 | PPSAL_2541 | BN5_2575 | 2-oxo-4-hydroxy-4-carboxy-5-ureidoimidazoline decarboxylase (EC 4.-.-.-) | Unknown | DapA |
| W6QWV6 | PPSAL_1861 | BN5_1882 | Protein tusC | Unknown | DapA |
| W6QQ91 | PPSAL_0498 | BN5_0501 | Aldo/keto reductase (EC 1.1.1.-) | Cytoplasmic | DapA |
| W6QWU7 | PPSAL_1851 | BN5_1872 | DNA translocase ftsK | Membrane | DapA |
| W6R6I9 | PPSAL_3394 | BN5_3444 | Response regulator receiver modulated diguanylate cyclase (EC 3.1.1.61) | Cytoplasmic | DapA |
| W6QRX5 | PPSAL_1084 | BN5_1088 | Oxidoreductase, GMC family (EC 1.1.99.1) | Membrane | DapA |
| W6QYH5 | PPSAL_0584 | BN5_0589 | ABC transporter related | Membrane | DapA |
| W6R1R3 | PPSAL_4075 | BN5_4138 | dTDP-4-dehydrorhamnose reductase (EC 1.1.1.133) | Cytoplasmic | DapA |
| W6R1K6 | PPSAL_4015 | BN5_4076 | Uncharacterized adenine-specific methylase MJECS02 (EC 2.1.1.72) | Cytoplasmic | DapA |
| W6QRF0 | PPSAL_0885 | BN5_0891 | Phospholipid/glycerol acyltransferase (EC 2.3.1.-) | Membrane | DapA |
| W6R267 | PPSAL_4194 | BN5_4258 | Uncharacterized protein | Unknown | DapA |
| W6RKJ4 | PPSAL_3704 | BN5_3761 | Extracellular solute-binding protein | Periplasmic | DapA |
| W6QVK9 | PPSAL_1419 | BN5_1424 | Deoxyguanosinetriphosphate triphosphohydrolase (EC 3.1.5.1) | Cytoplasmic | DapA |
| W6QY13 | PPSAL_2763 | BN5_2802 | Putative membrane protein | OuterMembrane | DapA |
| W6QYE7 | PPSAL_2444 | BN5_2474 | Two component transcriptional regulator | Cytoplasmic | DapA |
| W6QSN9 | PPSAL_0902 | BN5_0908 | DNA repair protein RecN (Recombination protein N) | Cytoplasmic | DapA |
| W6QY51 | PPSAL_2333 | BN5_2364 | GntR family transcriptional regulator | Cytoplasmic | DapA |
| W6RHK2 | PPSAL_2710 | BN5_2746 | Agglutinin receptor | Cytoplasmic | DapA |
| W6R104 | PPSAL_1492 | BN5_1497 | Threonine aldolase, low-specificity (EC 4.1.2.5) | Cytoplasmic | DapA |
| W6QVQ7 | PPSAL_1970 | BN5_1993 | Arsenate reductase (EC 1.20.4.1) | Cytoplasmic | DapA |
| W6QSB7 | PPSAL_1229 | BN5_1233 | Uncharacterized protein | Cytoplasmic | DapA |
| W6RC67 | PPSAL_0859 | BN5_0865 | Phospholipid/glycerol acyltransferase (EC 2.3.1.15) | Cytoplasmic | DapA |
| W6R2X6 | PPSAL_4449 | BN5_4513 | 50S ribosomal protein L34 | Cytoplasmic | DapA |
| W6R2U7 | PPSAL_4419 | BN5_4483 | Transposase for transposon Tn552 | Cytoplasmic | DapA |
| W6R0K8 | PPSAL_3157 | BN5_3202 | Simple sugar transport system ATP-binding protein (EC 3.6.3.17) | Membrane | DapA |
| W6RFQ3 | PPSAL_2117 | BN5_2143 | Uncharacterized protein | Cytoplasmic | DapA |
| W6RF39 | PPSAL_1902 | BN5_1925 | Aliphatic nitrilase (EC 3.5.5.7) | Cytoplasmic | DapA |
| W6RF25 | PPSAL_1887 | BN5_1910 | Cysteine synthase (EC 2.5.1.47) | Cytoplasmic | DapA |
| W6R1G7 | PPSAL_4302 | BN5_4366 | Transcriptional regulatory protein zraR | Cytoplasmic | DapA |

^1^Protein references correspond to UniProt accession numbers. Gene references correspond to GenBanK accession numbers HG916826 accession number^2^ (Wibberg *et al.*, 2014) and LK391695 accession number^3^ (Wibberg *et al.*, 2016). ^4^Subcellular location according with PSOTb v3.0.2. ^5^Fold change calculated as the ratio protein expression in the wild-type strain and the DapA1 ^─^ mutant (using the wild-type strain as reference).
