# Supplementary material for: Role of the Dihydrodipicolinate Synthase DapA1 on Iron Homeostasis During Cyanide Assimilation by the Alkaliphilic Bacterium Pseudomonas pseudoalcaligenes CECT5344
Source: Front Microbiol. 2020 Jan 23;11:28. doi: 10.3389/fmicb.2020.00028 (PMC6989483; doi:10.3389/fmicb.2020.00028)
Supplement: TABLE S3 — Bacterial strains and plasmids used in this work. [file Table_3.DOCX]

**Table S3.** Bacterial strains and plasmids used in this work.

| **Strain/plasmid** | **Genotype/characteristics** | **Reference** |
| --- | --- | --- |
| *Pseudomonas pseudoalcaligenes* CECT5344 | | |
| Wild type | Isolated by its ability to use cyanide as the sole nitrogen source. Nx^R^ | Luque-Almagro *et* *al*., 2005 |
| DapA1^─^ | Defective mutant in the *dapA* gene of the CECT5344. Nx^R^, Gm^R^ | This work |
| *Escherichia coli* | | |
| DH5α | Lac^─^, host for plasmids with *lacZ* gene | Sambrook *et* *al*., 1989 |
| S17-1 | Tra^+^; host for mobilizable plasmids with *mob* genes | Sambrook *et* *al*., 1989 |
| Plasmids | | |
| pGEM-T | Vector for routinary cloning of DNA fragments Ap^R^ | Promega |
| pK18mob | Mobilizable suicide vector in *Pseudomonas* derived from pK18. Km^R^ | Schafer *et al*., 1994 |
| pDapA1-1 | pGEM-T vector with a fragment of *dapA* gene. Ap^R^ | This work |
| pDapA1-2 | pGEM-T vector with a fragment of *dapA* gene disrupted by a gentamycin resistant cassete. Ap^R^, Gm^R^ | This work |
| pKDapA1-3 | pK18mob vector with a fragment of *dapA* gene. Km^R^, Gm^R^ | This work |

Nx^R^, nalidixic acid resistant; Gm^R^, gentamycin resistant; Ap^R^, ampicillin resistant; Km^R^, kanamycin resistant.
