# Supplementary material for: Role of the Dihydrodipicolinate Synthase DapA1 on Iron Homeostasis During Cyanide Assimilation by the Alkaliphilic Bacterium Pseudomonas pseudoalcaligenes CECT5344
Source: Front Microbiol. 2020 Jan 23;11:28. doi: 10.3389/fmicb.2020.00028 (PMC6989483; doi:10.3389/fmicb.2020.00028)
Supplement: TABLE S4 — Primers used in this work. [file Table_4.DOCX]

**Table S4.** Primers used in this work.

| **Methodology/Primer** | **Sequence (5' → 3')*** |
| --- | --- |
| DapA1-F | CGGGATCCGTCCGCTGGCATGTGCAGTAGGCGAGA (*Bam*HI) |
| DapA1-R | CCAAGCTTGCCATGGCACCGAAGATCCATCCCAGG (*Hin*dIII) |
| T7-F | TAATACGACTCACTATAGGG |
| BN5_0768-F | GATGGGCAGGTGCGTGAGCTGGGC |
| BN5_0768-R | GCGCGGTGGGTGATCGCCAGCAAC |
| BN5_0907-F | TCGACAACGCCGAGCACCGCCACA |
| BN5_0907-R | CAGGCCAACGTCCTCACCCGCCTCC |
| BN5_1139-F | CCGCACATTTCCGCCGAGACCCT |
| BN5_1139-R | TGCCCGGCACCAGGTTGTTCAGG |
| BN5_1910-F | CGGAAACACGCCACTGATCGCACT |
| BN5_1910-R | ATCCTTGACTGAGCCTCCGGGGTT |
| BN5_1911-F | CCTGCTGGTCTGCCCCAACGTGGA |
| BN5_1911-R | ACCCAACAGGAACGGCCCGACT |
| BN5_2037-F | ACAGTCCGCTCAACGAACGCATCA |
| BN5_2037-R | CTGCAGCACGGCAATGCCCTCT |
| BN5_2125-F | CCCTGCACCTGACCGGCGACCT |
| BN5_2125-R | GCGCTGTCCCAGTCCGTGCGTGA |
| BN5_2417-F | GAACCGCGCCCTGCCACTGATCCC |
| BN5_2417-R | GCCCGCCAGCACACCCTGACTCC |
| BN5_2718-F | TTCCGCCACATCGCCGAAGCCGTC |
| BN5_2718-R | CGCTCGACAGTCTCCGGCAGCA |
| BN5_3669-F | CACCTGGACGCAAACGCCGACCC |
| BN5_3669-R | CCGGCGACCTGACTTGCTCCCATT |
| BN5_4197-F | GCTGCTGCACCGCGAGGAACTGCT |
| BN5_4197-R | ACCTCCCAGACCAGGCCGGAGT |

(*) The enzyme restriction sites are underlined
